# Metabolomic-guided discovery of cyclic nonribosomal peptides from *Xylaria ellisii* sp. nov., a leaf and stem endophyte of *Vaccinium angustifolium*

**DOI:** 10.1038/s41598-020-61088-x

**Published:** 2020-03-12

**Authors:** Ashraf Ibrahim, Joey B. Tanney, Fan Fei, Keith A. Seifert, G. Christopher Cutler, Alfredo Capretta, J. David Miller, Mark W. Sumarah

**Affiliations:** 1 Department of Chemistry and Chemical Biology, McMaster University, Hamilton, Ontario L8S 4M1 Canada; 2 Department of Chemistry, Carleton University, Ottawa, Ontario K1S 5B6 Canada; 3 Pacific Forestry Centre, Canadian Forest Service, Natural Resources Canada, Victoria, British Columbia V8Z 1M5 Canada; 4 Ottawa Research and Development Centre, Agriculture and Agri-Food Canada, Ottawa, Ontario K1A 0C6 Canada; 5 Department of Plant, Food, and Environmental Sciences, Faculty of Agriculture, Dalhousie University, Truro, NS B2N 5E3 Canada; 6 London Research and Development Centre, Agriculture and Agri-Food Canada, London, Ontario N5V 4T3 Canada; 7 Present Address: LifeMine Therapeutics, Cambridge, Massachusetts 02140 USA

**Keywords:** Mass spectrometry, Fungal ecology, Chemical ecology

## Abstract

Fungal endophytes are sources of novel bioactive compounds but relatively few agriculturally important fruiting plants harboring endophytes have been carefully studied. Previously, we identified a griseofulvin-producing *Xylaria* species isolated from *Vaccinium angustifolium, V. corymbosum*, and *Pinus strobus*. Morphological and genomic analysis determined that it was a new species, described here as *Xylaria ellisii*. Untargeted high-resolution LC-MS metabolomic analysis of the extracted filtrates and mycelium from 15 blueberry isolates of this endophyte revealed differences in their metabolite profiles. Toxicity screening of the extracts showed that bioactivity was not linked to production of griseofulvin, indicating this species was making additional bioactive compounds. Multivariate statistical analysis of LC-MS data was used to identify key outlier features in the spectra. This allowed potentially new compounds to be targeted for isolation and characterization. This approach resulted in the discovery of eight new proline-containing cyclic nonribosomal peptides, which we have given the trivial names ellisiiamides A-H. Three of these peptides were purified and their structures elucidated by one and two-dimensional nuclear magnetic resonance spectroscopy (1D and 2D NMR) and high-resolution tandem mass spectrometry (HRMS/MS) analysis. The remaining five new compounds were identified and annotated by high-resolution mass spectrometry. Ellisiiamide A demonstrated Gram-negative activity against *Escherichia coli* BW25113, which is the first reported for this scaffold. Additionally, several known natural products including griseofulvin, dechlorogriseofulvin, epoxy/cytochalasin D, zygosporin E, hirsutatin A, cyclic pentapeptides #1–2 and xylariotide A were also characterized from this species.

## Introduction

*Vaccinium angustifolium* (wild lowbush blueberries or commonly wild blueberries) were consumed fresh and preserved for the winter by the Indigenous peoples of northeastern North America and rapidly incorporated into the diets of European settlers in Canada from the early 17^th^ century^1,2^. Today, blueberries comprise more than half of all fruit production in Canada. Wild blueberries often grow in forests where *Pinus strobus* (eastern white pine) is the dominant tree species. Eastern white pine is an economically, ecologically, and culturally important keystone tree species in eastern N. American forests, especially for bird species^3,4^.

Endophytes are an ecological category of phylogenetically diverse fungi that can asymptomatically colonize healthy plant tissues. Ascomycetous endophytes of various species of *Vaccinium* have been reported over the past three decades. This includes from surface-sterilized tissues of *Vaccinium vitis-idaea* (lingonberry, European blueberry) and *V. myrtillus* (bilberry, whortleberry) in Europe^5^, *V. dunalianum* var. *urophyllum* (South China blueberry) in China^6^, as well as from stems of *V. macrocarpon* (cranberries) and *V. corymbosum* (northern highbush blueberry) in New Jersey^7,8^. There is some evidence of the same endophyte species occurring in both conifer and *Vaccinium* species, e.g. *Nemania diffusa* (Xylariaceae)^6,9,10^ and Phacidiaceae species such as *Allantophomopsis lycopodina*, *Phacidium lacerum*, and *Strasseria geniculata*^11–15^. Indirect evidence of a conifer-*Vaccinium* shared endophyte includes the aquatic hyphomycete *Dwayaangam colodena*, a common needle endophyte of *Picea* spp., which was reported from rainwater collected from foliage of *Picea abies, Pinus sylvestris*, and *Vaccinium myrtillus* in Europe^16–19^. Discovery of an aquatic hyphomycete conifer endophyte and reports of hardwood saprotrophs as conifer endophytes (e.g. *Phialocephala piceae*^20^) are evocative of more complex interactions between endophytes and their environment.

Endophytes belonging to the family Xylariaceae (Xylariales, Sordariomycetes) are ubiquitous and detected in varying abundance in most studies involving woody plants, regardless of geographic location or host, whether by isolation of cultures or by studies of DNA, often exhibiting little host preference and including known saprotrophs^21–24^. Xylariaceae endophytes are common but difficult to identify to species because of a lack of reference sequences and the limited taxonomic resolution of the asexual states (usually the only morphological characters produced *in vitro*). However, careful field observations can provide connections between the often conspicuous Xylariaceae stromata found in nature and the corresponding endophytes isolated in culture or detected by DNA sequences from the same forests^23–25^. Taxonomically, Xylariaceae comprises at least 37 genera with likely more than 1,000 species^26^. Many endophyte studies based on morphological identification of cultures report geniculosporium-like morphs attributable to *Anthostomella, Rosellinia* and *Xylaria* species^27–31^. The classical nature of most taxonomic studies of Xylariaceae is reflected by the need for the sexual state to confirm identification, with a relative paucity of species-specific DNA barcodes and phylogenetic markers compared to many other ascomycete groups. Thus, xylariaceous endophytes may include species and genera known to classical taxonomy but not included in sequence databases (i.e.: named-but-unsequenced species).

Species of Xylariaceae are a rich source of secondary metabolites, and chemotaxonomy is often part of taxonomic studies. Species in this family can produce diverse metabolites from multiple biosynthetic families including dihydroisocoumarins, punctaporonins, cytochalasins, butyrolactones, and succinic acid derivatives^32,33^. Exploration of *Xylaria* metabolites using newer chemical methods led to discovery of a broad array of metabolites from both tissues of stromata and culture extracts^34^.

Although there have been many studies of metabolites from fungal endophytes^35,36^, there are few reports from endophytes of *Vaccinium*^37,38^. We previously described production of the antifungal compounds griseofulvin and piliformic acid from an unknown *Xylaria* species isolated as a foliar endophyte from wild blueberry in natural and commercial sites, and from white pine. After the Richardson *et al*. (2014) study, we continued to isolate the same unidentified species of *Xylaria* as an endophyte of white pine needles and as an endophyte of leaves and stems of both wild and highbush blueberry at three different locations in Nova Scotia, New Brunswick, and Ontario, Canada^38^. We also conducted field sampling to discover the putative sexual state of this unknown *Xylaria* species. This would provide information on morphological characters of sexual structures, permitting its identification. This previously unknown endophyte is described here as *Xylaria ellisii* based on morphological and genomic evidence. Representative sequences in NCBI GenBank from other studies indicate that *X. ellisii* has been isolated many times as an unidentified endophyte from a wide variety of plant hosts, allowing us to infer additional information about its distribution, biology, and chemistry.

**Figure 1:**
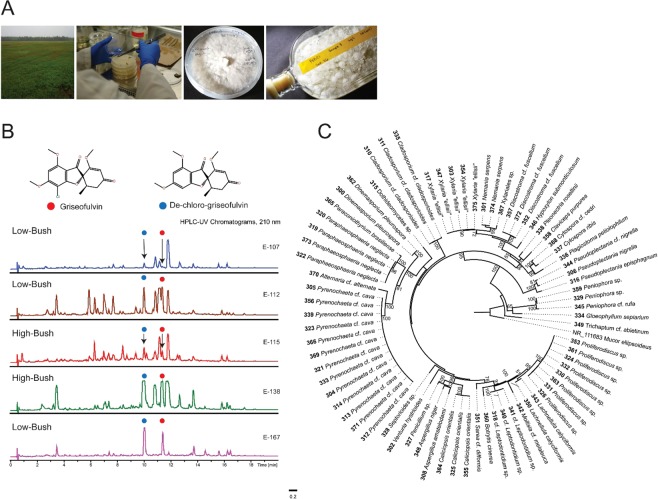
Discovery of new griseofulvin-producing fungal endophyte species *Xylaria ellisii* isolated from highbush and wild blueberry leaves and stems. (**A**) Isolation and culturing of fungal endophyte, (**B**) LC-UV comparative profile analysis of crude filtrate extracts at λ 210 nm, revealing differences in metabolite production, (**C**) Most likely tree from a RAxML analysis of ITS dataset containing representative endophytes. Culture numbers precede the species name and RAxML bootstrap support percentages ≥50 from a summary of 1000 replicates are presented at the branch nodes. This tree was rooted with *Mucor ellipsoideus* (ATCC MYA-4767; NR_111683) and the scale bar represents the number of substitutions per site.

In our effort to discover novel natural products, we applied a LC-MS metabolomic-guided discovery approach to these *Xylaria* strains from wild and highbush blueberry plants (Fig. 1). This approach allows for a global survey of small molecule metabolites from an extract and visual representation of metabolite variances between groupings or extracts. Thus, discriminating between like and different features allows extracts to be prioritized for further investigation^39,40^. Fifteen strains were grown on two media and the resulting ethyl acetate extracted filtrates and mycelium were screened using standardized LC-UV/MS conditions. Multivariate statistical analysis was used to organize resulting analytical data to reveal extracts that appeared to have differences in their major secondary metabolites. This approach led to the discovery of a family of eight new proline-containing cyclic nonribosomal pentapeptides named ellisiiamides A–H. Ellisiiamide A is an alanine (Ala) substituted variant, a first report for this scaffold, and demonstrated modest activity against *Escherichia coli*.

## Results

### Identification, biology and ecology of *Xylaria* sp

Approximately 30 strains of *Xylaria* sp. were isolated from surface-sterilized blueberry tissues collected from highbush and wild blueberry fields within a ~300 × 100 km triangular area. All fields were surrounded by forested lands. Preliminary phylogenetic analysis using the internal transcribed spacer (ITS) barcode combined with morphological features confirmed conspecificity of isolated endophytic *Xylaria* sp. strains. However, identification of the strains to species was not possible using molecular or *in vitro* morphological data. Based on a BLAST query of the *Xylaria* sp. ITS and *RPB2* sequences with available GenBank sequences, the endophyte strains were closest related to sequences identified as *Xylaria berteri*, *X. castorea*, *X. cubensis*, *X. laevis*, and *X. longipes*, species that form conspicuous sexual reproductive structures (stromata) from decaying hardwood. Given the close phylogenetic relationship of the unknown *Xylaria* endophyte to these species and evidence of prevalent endophytic-saprotrophic life histories within Xylariaceae^23–25,41,42^, we inferred that the unknown *Xylaria* endophyte likely produces stromata from decaying hardwood in mixedwood stands in the Acadian forest. Thus, *Xylaria* stromata were selectively sampled during ongoing field surveys to collect the putative sexual state of the endophyte. This would provide material for identification and insight into its life history^20,43^.

**Figure 2:**
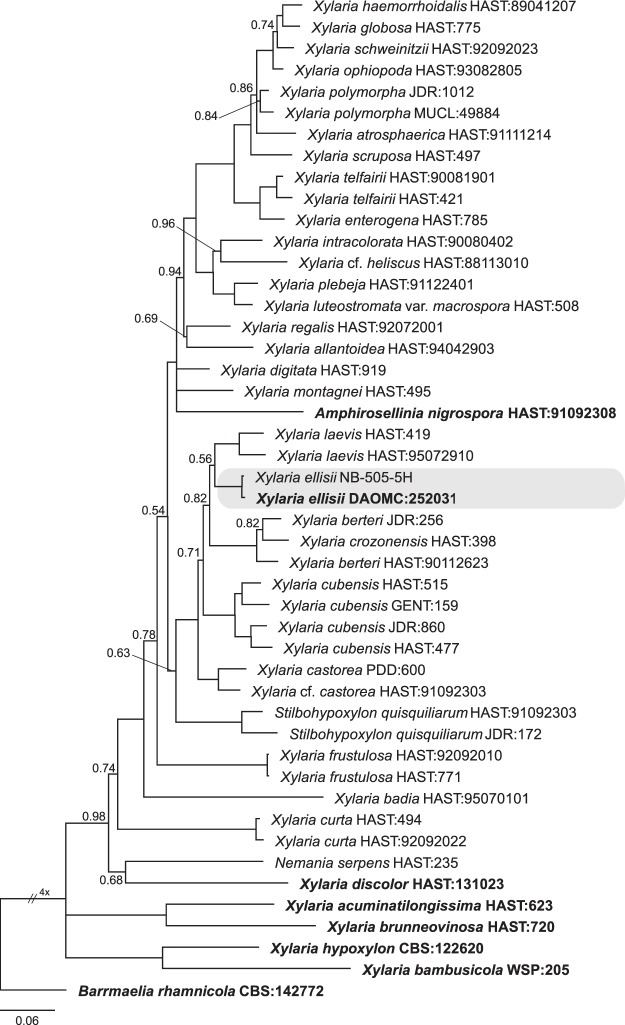
Bayesian 50% majority rule *RPB2* consensus tree containing *Xylaria ellisii* and related species. All unlabeled branches have Bayesian posterior probability values of 1.0; values lower than 1.0 are presented at nodes. The tree was rooted to *Barrmaelia rhamnicola* (CBS:142772) and the scale bar indicates the expected number of changes per site. Strain numbers follow species names and type specimens are indicated in bold.

A *Xylaria* sp. producing stroma reminiscent of *X. corniformis* and *X. curta* was collected from decaying, often partially buried, *Acer saccharum* branches or logs in late summer and autumn. Sequences (ITS, SSU, LSU, *BenA*, *EF1-α, RPB2*) obtained from stromatal tissue and ascospore cultures were identical to those obtained from the *Xylaria* sp. endophyte cultures, indicating they are conspecific and evincing a saprotrophic-endophytic life history. Based on morphological study of the stromata, this species is equivalent to *X*. *corniformis* var. *obovata* Sacc., *Xylaria corniformis sensu* Laessøe^44^, and *Xylaria curta sensu* Rogers^45^. From the *RPB2* phylogeny, *X*. *corniformis* var. *obovata* is weakly supported (posterior probability value (PP) = 0.56) sister to *X. laevis* and other species within the strongly-supported (PP = 1.0) *X. cubensis* aggregate clade. *Xylaria* is polyphyletic, including *Amphirosellinia nigrospora*, *Stilbohypoxylon quisquiliarum*, and *Nemania serpens*, and the type species (*X. hypoxylon*) occurs in a basal clade sister to *X. bambusicola*. Additional *RPB2* sequences for related *Xylaria* species are needed to generate a more comprehensive phylogeny (Fig. 2).

**Figure 3:**
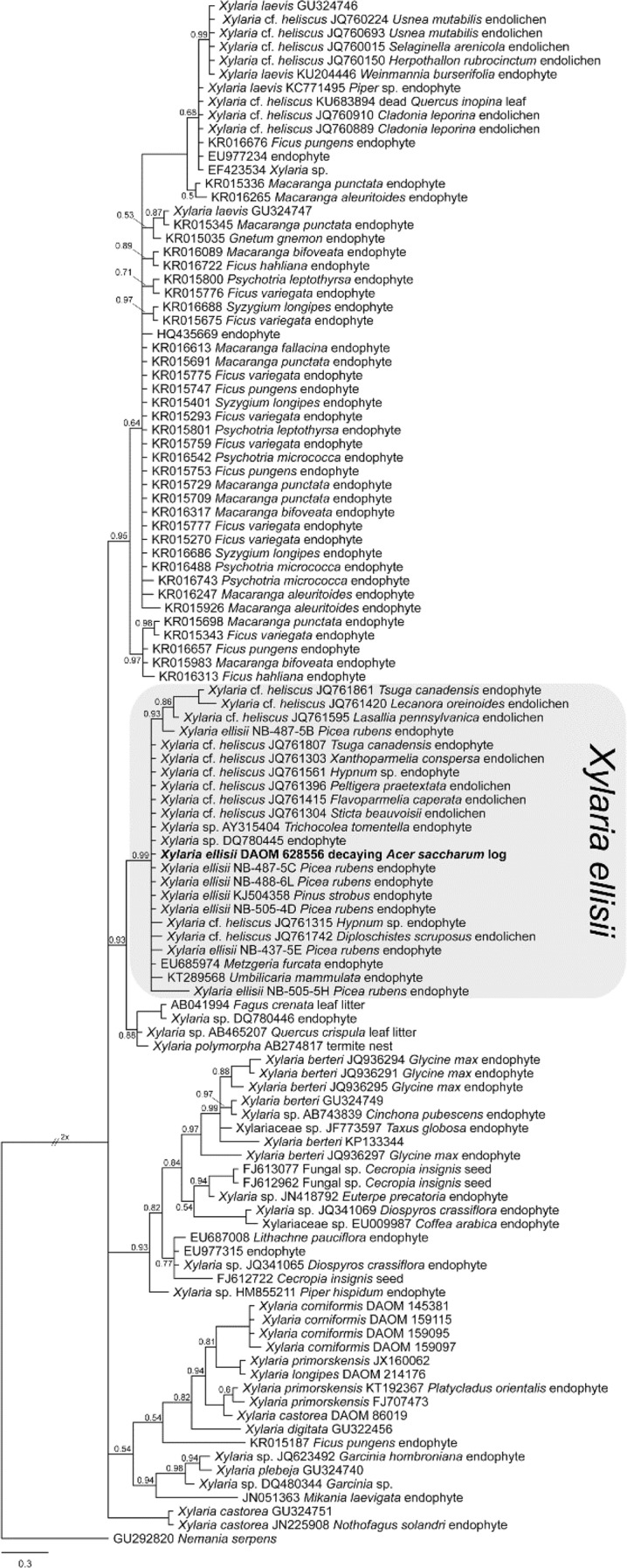
Bayesian 50% majority rule ITS consensus tree containing *Xylaria ellisii* and related species. All unlabeled branches have Bayesian posterior probability values of 1.0; values lower than 1.0 are presented at nodes. The tree was rooted to *Nemania serpens* and the scale bar indicates the expected number of changes per site. GenBank accession numbers and host information follow species names (when applicable). Type specimens are indicated in bold.

Several DAOMC herbarium specimens identified as *X. corniformis* from *Acer* spp. wood in Ontario and Quebec were morphologically similar to *X. laevis*. The resulting ITS sequences from these specimens showed that they formed a clade sister to *X. longipes* and *X. primorskensis* and were distinct from the griseofulvin-producing *X*. *corniformis* var. *obovata* (Fig. 3). We support the distinction of *X. corniformis* var. *obovata* from *X. corniformis*, and thus describe a new species, *Xylaria ellisii*, to accommodate its novelty and fulfill the need to delineate boundaries in species complexes with robust species concepts connected to authenticated reference sequences and specimens.

### LC-MS analysis of culture extracts and multivariate data analysis

Fifteen strains of *X. ellisii* were subject to further study: four from cultivated highbush blueberry plants and 11 from wild blueberry plants. Ethyl acetate extracts of the culture filtrate and associated mycelium were screened using standardized LC-UV/MS conditions.

**Figure 4:**
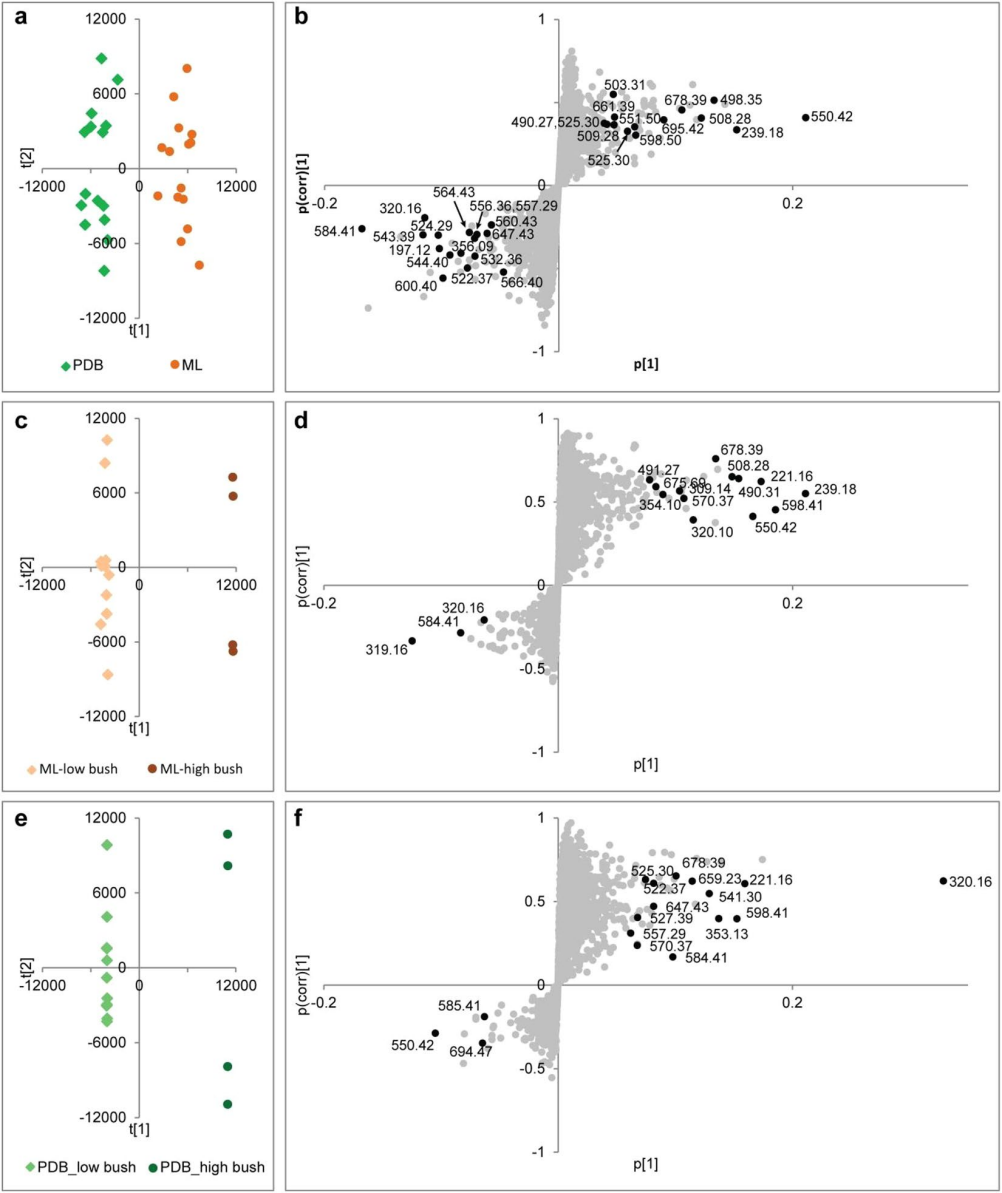
Supervised multivariate analyses of extracted filtrates from blueberry isolates of *X. ellisii* endophytes. The OPLS-DA score plot (**a**) and S-plot (**b**) for comparison between *X. ellisii* endophytes cultured in ML or PDB media. The OPLS-DA score plots and S-plots compared the *X. ellisii* endophytes isolates from highbush or wild blueberries cultured in ML (**c**,**d**) or PDB (**e**,**f**) medium, respectively.

In order to identify unique secondary metabolite differences between extracts of *Xylaria* isolates of highbush and wild blueberry plants we compared the extracted filtrates and mycelium with three different pair-wise comparisons. These comparisons included: ethyl acetate extracts of *Xylaria* strains grown on 2% malt extract broth (ML) versus those grown in potato dextrose broth (PDB) cultures; ML media cultures of highbush versus wild varieties; and, PDB medium cultures of highbush versus wild isolates (Fig. 1S). A supervised multivariant analysis method, Orthogonal Partial Least Squares Discriminant Analysis (OPLS-DA), was used to identify outlier metabolites biosynthesized under the different culture conditions tested. OPLS-DA correlates differences in secondary metabolite feature abundances (X variables) to various treatment groups (Y variables) by identifying principle components that describe differences. R^2^X, R^2^Y, and Q^2^ parameters are important validation parameters used for OPLS-DA, where R^2^X and R^2^Y describes the percentage of X and Y variables described by the model (Fig. 4 and Supplementary Fig. 2S). A valid model is defined as having a prediction statistic of Q^2^ > 0.4, with values above 0.7 being highly significant^46^. Metabolite features with a high Variable Importance in Projection (VIP) scores (>0.7) are responsible for driving the differences between treatment groups, and these values are considered significant^47^. Their metabolic features can be viewed at both ends of the OPLS-DA S-plot.

Fractions with VIP scores above 0.7 were selected for further study and compounds were identified where possible. OPLS-DA validation parameters for each of the extracted filtrates and mycelium metabolite models tested are summarized in Table 1 and Supplementary Table 1S. In total, 3856 metabolite features were identified from the extracted filtrates, with a Q^2^ value of 0.615 for ML versus PDB, and Q^2^ values of 0.778 for ML and PDB, as well as highbush versus wild varieties.

**Table 1:** A summary of validation parameters (R^2^X, R^2^Y, Q^2^) of all calculated OPLS-DA models for extracted filtrates of *X. ellisii* endophytes isolates from wild and highbush blueberries cultured in ML and PDB media. ML-H, endophyte isolates from highbush blueberries cultured in ML medium; ML-L, endophyte isolated from wild blueberries cultured in ML medium; PDB-H, endophyte isolates from highbush blueberries cultured in PDB medium; PDB-L, endophyte isolates from wild blueberries cultured in PDB medium.

Model	Variables*	R^2^X(cum)	R^2^Y(cum)	Q^2^(cum)	Conditions
1a	3856	0.15	0.939	0.615	ML, PDB
1b	100	0.313	0.935	0.737	ML, PDB (including top 100 VIP)
1c	3756	0.299	0.982	0.434	ML, PDB (excluding top 100 VIP)
1d	3556	0.207	0.954	0.394	ML, PDB (excluding top 300 VIP)
2a	3856	0.477	0.998	0.778	ML-H, ML-L
2b	30	0.861	0.984	0.864	ML-H, ML-L (including top 30 VIP)
2c	3826	0.544	1	0.718	ML-H, ML-L (excluding top 30 VIP)
2d	3156	0.121	0.73	-0.187	ML-H, ML-L (excluding top 700 VIP)
3a	3856	0.648	1	0.778	PDB-H, PDB-L
3b	50	0.589	0.995	0.885	PDB-H, PDB-L, (including top 50 VIP)
3c	3806	0.651	1	0.668	PDB-H, PDB-L (excluding top 50 VIP)
3d	2956	0.0851	0.894	-0.175	PDB-H, PDB-L (excluding top 900 VIP)

*Number of metabolomic features included in the OPLS-DA analysis.

### Metabolomic-guided discovery and metabolite identification of knowns 1–11

We first evaluated metabolites with the top VIP (30, 50, 100) scores for ethyl acetate extracts of the filtrates and methanol/acetone (1:) extracted mycelia from *Xylaria*. The initial focus was on metabolites that displayed UV absorption maxima at ~210, 254, 275 or 350 nm (Table 2 and Supplementary Tables S1–8). Compounds (100–2000 μg) were purified by reverse phase semi-preparative HPLC and characterized by NMR (Bruker Advance III 700 MHz NMR with cryoprobe) (Supplementary Fig. 1S). Metabolites were dereplicated against natural product databases including Antibase (https://www.wiley.com/en-us/AntiBase%3A+The+Natural+Compound+Identifier-p-9783527343591), Dictionary of Natural Products (http://dnp.chemnetbase.com/faces/chemical/ChemicalSearch.xhtml) and NORINE (https://bioinfo.lifl.fr/norine/) using molecular formulas dictated by HRMS data. In addition, a comparative analysis was conducted against known fungal metabolites^48–51^. Using this dereplication approach, the previously reported compounds **1**–**11** were identified; (**1**) griseofulvin, (**2**) dechlorogriseofulvin, (**3**) cytochalasin D, (**4**) zygosporin E, (**5**) epoxycytochalasin D, (**6**) hirsutain A, (**7**) pilformic acid, (**8**) 2, 3-dihydro-2,4-dimethylbenzofuran-7-carboxylic acid, (**9**) cyclic pentapeptide 1, (**10**) xylarotide A, and (**11**) cyclic pentapeptide 2 (Table 2). LC-HRMS, NMR, and spectroscopic data for compounds **1**–**11** confirming their structures can be found in the Supplementary Methods, Figs. 2 and 3S, 32–49S and Tables 1–9S).

**Table 2:** Identification of known and new secondary metabolites from *X. ellisii* via LC- UV/HRMS and LC-HRMS/MS analysis.

#	Compound	Class	Rt	Molecular Formula	Measure and Calculated [M+H]^+^		ppm error
**Known**							
1	Griseofulvin	PKS	11.42	C_17_H_18_ClO_6_	353.0793	353.0786	−1.98
2	Dechlorogriseofulvin*	PKS	10.01	C_17_H_19_O_6_	319.1173	319.1176	0.94
3	Cytochalasin D*	PKS-NRPS	11.81	C_30_H_38_NO_6_	508.2687	508.2694	1.38
4	Zygosporin E*	PKS-NRPS	13.94	C_30_H_38_NO_5_	492.2742	492.2744	0.41
5	Epoxycytochalasin D	PKS-NRPS	10.87	C_30_H_38_N0_7_	524.2651	524.2661	1.91
6	Hirsutatin A*	NRPS	15.85	C_34_H_53_N_4_O_10_	677.3741	677.3756	2.21
7	Piliformic acid	PKS	10.84	C_11_H_18_O_4_Na	237.1094	237.1097	1.27
8	2,3-dihydro,2,4- dimethylbenzofuran -7- carboxylic acid	PKS	11.15	C_11_H_13_O_3_	193.0857	193.0859	1.04
9	Cyclic pentapeptide 1*	NRPS	16.20	C_32_H_50_N_5_O_5_	584.3816	584.3806	−1.71
10	Xylarotide A	NRPS	15.97	C_29_H_52_N_5_O_5_	550.3973	550.3963	−1.82
11	Cyclic pentapeptide 2	NRPS	14.28	C_28_H_50_N_5_O_5_	536.3819	536.3806	−2.42
**New**							
12	Ellisiiamide A*	NRPS	14.76	C_30_H_46_N_5_O_5_	556.3501	556.3493	−1.44
13	Ellisiiamide B*	NRPS	15.19	C_31_H_48_N_5_O_5_	570.3656	570.3650	−1.05
14	Ellisiiamide C*	NRPS	17.04	C_33_H_52_N_5_O_5_	598.3968	598.3963	−0.84
15	Ellisiiamide D	NRPS	14.47	C_27_H_48_N_5_O_5_	522.3662	522.3650	−2.30
16	Ellisiiamide E	NRPS	16.89	C_30_H_54_N_5_O_5_	564.4132	564.4119	−2.30
17	Ellisiiamide F	NRPS	14.11	C_31_H_48_N_5_O_6_	586.3616	586.3599	−1.72
18	Ellisiiamide G	NRPS	14.00	C_32_H_50_N_5_O_6_	600.3768	600.3756	−2.00
19	Ellisiiamide H	NRPS	14.89	C_33_H_52_N_5_O_6_	614.3936	614.3912	−2.41

Select metabolites have been further isolated and characterized by 1D and 2D NMR.

*Structures elucidated by 1D and 2D NMR, HRMS and MS/MS analysis.

### Structure elucidation of ellisiiamides A-C (12–14)

**Figure 5:**
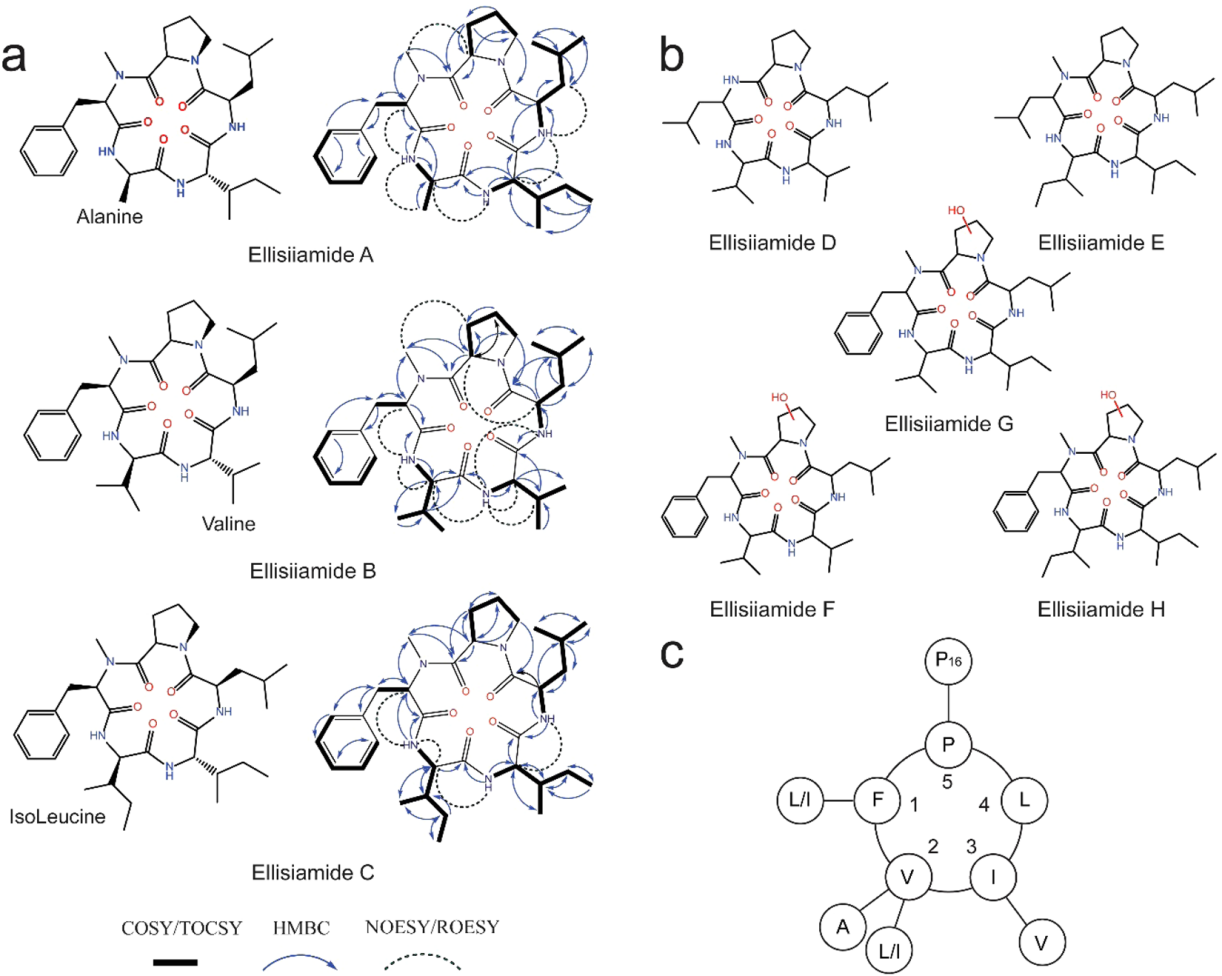
Ellisiiamides A–H (**12–19**), new cyclic nonribosomal peptides from *Xylaria ellisii*. (**a**) ellisiiamides A–C (**12–14**) isolated and characterized by 1D and 2D NMR, LC-HRHMS and LC-HRMS/MS analysis with new amino acid substituent highlighted. Corresponding COSY/TOCSY (^1^H -^1^H), HMBC (^1^H-^13^C) and long-range through-space NOESY/ROESY correlations are shown. (**b)** Structures of ellisiiamides D–H (**15–19**) based on LC-MS/MS, comparative LC-MS/MS analysis of cyclic pentapeptides **9**, **11** and **12–14** (**c)** Amino acid scaffold of the cyclic pentapeptide family of compounds. Cyclic pentapeptide 1 (**9**) shown with established amino acid substituents.

Ellisiiamides A–C (**12**–**14)** were identified by metabolomic analysis of the extracted filtrates and mycelium with high VIP scores (2.6–11.59; Fig. 4, Supplementary Tables 1–8S). These new cyclic pentapeptides are structurally similar to cyclic pentapeptide 1 (**9**), with amino acid differences at positions 2 (Ala/IsoLeu *vs*. Val) and 3 (Val *vs*. IsoLeu) within the peptide scaffold (Fig. 5 and Supplementary Fig 5S, Tables 9–12S).

Ellisiiamide A (**12**) was isolated as a white powder and afforded a protonated molecular ion at *m/z* 556 (C30H45N5O5 with 11 double bond equivalents). Examination of the ^1^H and ^13^C NMR data revealed the presence of five α protons (*δ* 5.08/55.8, 4.49/46.0, 4.17/55.7, 4.74/46.5, 5.10/58.9 ppm), and three key amide N-H protons for Ala (δ 8.52), ΙsoLeu (δ 6.94) and Leu (δ 8.49) and the N-Methyl group at (δ 3.04/30.2 ppm). Examination of the multiplicity edited ^1^H-^13^C HSQC, ^1^H-^13^C HMBC, and ^1^H-^1^H COSY NMR data revealed the individual amino acid spin systems within the peptide scaffold based on α proton correlations to the individual carbonyl carbon, including amide protons to neighboring amino acid carbonyls, and α, *β* and γ proton correlations (Fig. 5, Supplementary Figs. 4–13S and Tables 9 and 10S). These correlations supported the amino acid sequence of cyclo-(NMePhe-Ala-IsoLeu-Leu-Pro). NOESY through-space correlations of αΗ(Ν−MePhe)/NH (Ala), NH(Ala)/*β* Η (Ala), NH(IsoLeu)/αΗ (Ala), NH(Leu)/αΗ (IsoLeu) and H3-NMe (N-MePhe)/*β* Η (Pro) further supported the amino acid sequence and relative stereochemistry. Analysis of the LC-MS/MS spectra of ellisiiamide A revealed key diagnostic b-ion fragments of *m/z* 459.3 (-Pro), 346.2 (-Leu), 233.1 (-IsoLeu), 162.1 (-Ala) and the presence of two fragmentation pathways as seen in cyclic pentapeptide 1 with ring-opening cleavage events at the N-MePhe-Pro and Pro-Leu sites^52^.

Ellisiiamide B (**13**) was isolated as a white powder with a protonated molecular ion at *m/z* 570 affording a molecular formula of C31 H47N5O5 with 11 double bond equivalents. Examination of ^1^H and ^13^C NMR data revealed presence of five α protons (δ 5.10/56.0, 3.95/57.6, 4.10/56.8, 4.72/46.6, 5.08/58.7 ppm), key amide N-H protons for Val (δ 8.18), Val2 (δ 6.98), and Leu (δ 8.43), and the N-Methyl group at (δ 3.03/30.2 ppm). Ellisiiamide B (**13**) differs from (**9**) with Val substituted for IsoLeuc at position # 3 (Fig. 4, Supplementary Fig. 4S and 14–21S, Table 9 and 11S). Examination of the MS/MS spectra revealed a similar fragmentation pattern as in (**9**) and (**12**), with key diagnostic b-ion fragment ions at *m/z* 471.3 (-Pro), 360.2 (-Leu), 261.2 (-Val), and 162.1 (-Val). The cyclo-(NMePhe-Val1-Val2-Leu-Pro) amino acid sequence was confirmed with key HMBC correlations of αΗ(Ν−MePhe)/CO (N-MePhe), H3-NMe (N- MePhe)/CO (Pro), αΗ(Val1)/CO(Val1), αΗ(Val2)/CO(Val2), αΗ (Leu)/CO (Leu), and αΗ (Pro)/CO (Pro). Key NOESY correlations of αΗ(Ν−MePhe)/NH(Val1), NH(Val1)/αΗ (Val1), NH(Val2)/αΗ (Val1), NH(Val2)/αΗ (Val2), NH(Val2)/*β* Η (Val2), NH(Leu)/ΝΗ (Val2), and H3-NMe (N-MePhe)/*β* Η (Pro) further supported the assignments.

Ellisiiamide C (**14**) was isolated as a white powder with a protonated molecular ion at *m/z* 598 affording a molecular formula of C_33_H_51_N_5_O_5_ with 11 double bond equivalents. Examination of ^1^H and ^13^C NMR data revealed the presence of five α protons (δ 5.10/56.0, 4.05/55.8, 4.25/55.1, 4.72/46.5, 5.09/58.7 ppm), key amide N-H protons for IsoLeu1 (δ 8.12), IsoLeu2 (δ 6.94) and Leu (δ 8.45), and the N-Methyl group at (δ 3.04/30.2 ppm). Ellisiiamide C (**14**) differs from (**9**) with IsoLeu substituted for Val at position # 2 (Fig. 4, Supplementary Fig. 4S and 22–31S, and Tables 9 and 11S). Examination of the ^1^H-^1^H COSY, multiplicity edited ^1^H-^13^C HSQC and HMBC NMR data revealed the individual spin system for the new IsoLeuc group with correlations of *β*−3Η(IsoLeu)/αΗ (IsoLeu) and δΗ(IsoLeu)/βΗ (IsoLeu). Correlations of the remaining α protons to the individual carbonyl carbons, amide protons to neighboring amino acid carbonyl, and HMBC α, *β* and γ proton correlations for Leu, Pro and N-MePhe is consistent with the cyclic peptide scaffold (Supplementary Table 9S.). NOESY through-space correlations of αΗ(Ν−MePhe)/NH (IsoLeu1), NH (IsoLeu1)/NΗ (IsoLeu2), NH (Leu)/αΗ (IsoLeu2)) further supported the amino acid sequence. Analysis of the MS/MS spectra of (**14**) revealed key diagnostic b-ion fragments of *m/z* 501.3 (-Pro), 388.3 (-Leu), 275.2 (-IsoLeu2), and 162.1 (-IsoLeu1) further confirming the amino acid sequence of cyclo-(N- MePhe-IsoLeu1-IsoLeu2-Leu-Pro).

The optical rotation for ellisiiamides A–C were measured at [α]^21^ -86.1 (0.06, MeOH), [α]^21^ -43.1 (0.04, MeOH), and [α]^20^ -47.8 (0.06, MeOH) respectively, and were consistent with **9** at [α]^21^ -63.4 (0.18, MeOH) (Supplementary Table 9S).

### LC-MS/MS analysis and putative identification of new cyclic pentapeptides

Ellisiiamide D–H (**15–19)** was identified by metabolomic analysis of the extracted filtrates and mycelium models as unique outliers with high VIP scores (1.92–6.46). Evaluation of the HRESIMS derived molecular formulas and MS/MS fragmentation patterns of (**15**–**19**) indicated that the fragmentation sequence and ring-opening events were consistent with ellisiiamide A–C and cyclic pentapeptide 1. We have therefor assigned putative identification and annotated structures for ellisiiamides D–H. LC-HRMS/MS characterization data can be found in the Supporting Methods and Figs. 4S and Table 1S and 9S.

### Bioactivity activity screening

Compounds **9** and **12**–**14** were screened for biological activity against three species of microorganisms in accordance with the Clinical Laboratory Standards Institute (CLSI) protocols (National Committee for Clinical Laboratory Standards, 2000, 1997). The microorganism included *E. coli* BW25113 ΔbamBΔtolC, *Saccharomyces cerevisiae* B4741, and *Candida albicans* ATCC# 90028.

Ellisiiamide A (**12**) showed modest activity against *E. coli* with a minimum inhibitory concentration (MIC) of 100 μg/mL. Such activity against *E. coli* is a first report for the cyclic pentapeptide scaffold. Compound **9** showed no antifungal activity against *S. cerevisiae* or *C. albicans* at 100 μg/mL, which is consistent with reported data^52^. Similarly, compounds **13**–**14** showed no activity against any test microorganisms at concentrations between 50–200 μg/mL.

### Taxonomy of *Xylaria ellisii*

#### *Xylaria ellisii*

**Figure 6:**
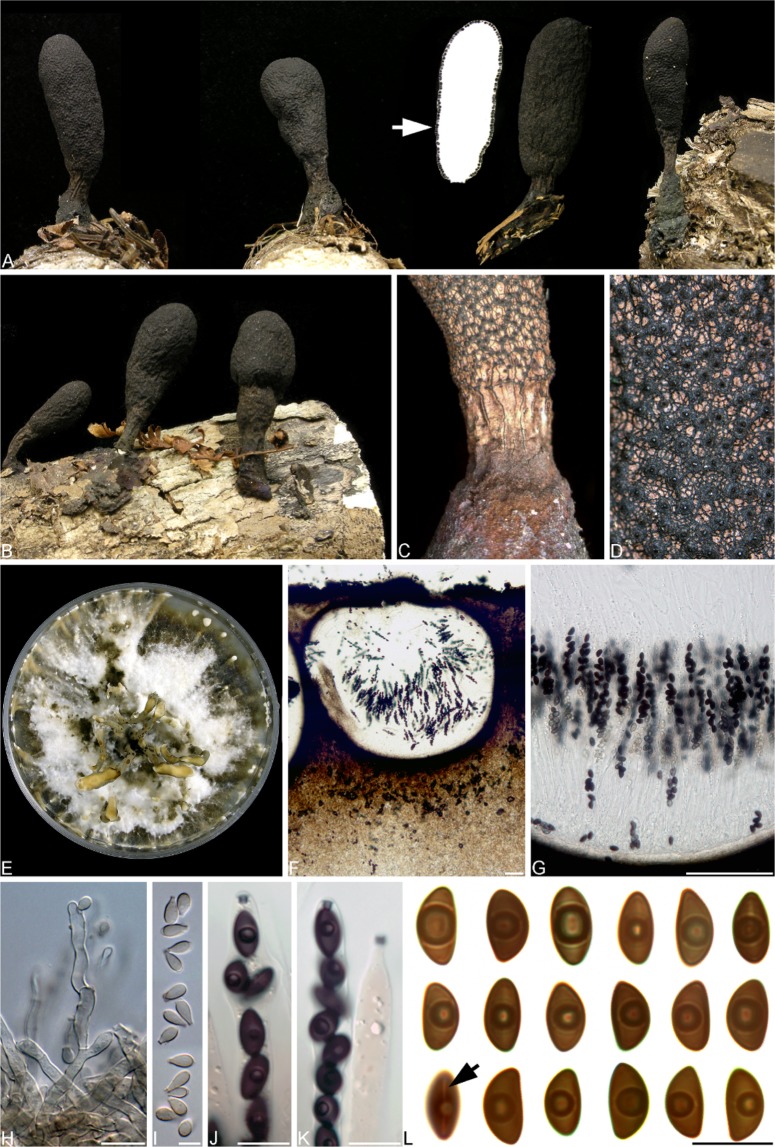
*Xylaria ellisii* morphology. (**A**,**B**) Stromata on partially buried, decaying *Acer saccharum* branches, arrow pointing to longitudinal section of stroma with perithecia lining outer surface. (**C**) Base of stroma showing ostioles and reticulations. (**D**) Ostioles on stroma surface. (**E**) Eight-week-old colony on oatmeal agar. (**F**) Longitudinal section of perithecium. (**G**) Asci and paraphyses. (**H**) Conidiogenous cells. (**I**) Conidia. (**J**,**K**) Asci with amyloid, inverted hat-shaped apical apparatuses. (**L**) Ascospores, arrow denoting germ slit. Scale bars: (**F**,**G**) = 100 µm, (**H**,**J**–**L**) = 10 µm, I = 5 µm.

J.B. Tanney, Seifert & Y.M. Ju, sp. nov. MycoBank MB832257 (Fig. 6)

= *Xylaria corniformis* (Fr.: Fr.) Fr. var. *obovata* M.C. Cooke & J.B. Ellis, Grevillea 6: 92. 1878.

#### Etymology

Named for the prolific mycologist Job Bicknell Ellis who, with Mordecai Cubitt Cooke, described *Xylaria corniformis* var. *obovata* Sacc., a synonym of *X. ellisii*.

Typus. Canada: New Brunswick, Alma, Fundy National Park, East Branch Trail, 45.6433 -65.1156, stromata on partially buried, mostly decorticated *Acer saccharum* branch, 28 Sep 2014, J.B. Tanney NB-623 (holotype DAOM 628556). Ex-type culture DAOMC 252031.

Colonies 32–38 mm diam after 14 d in the dark at 20 °C on MEA; white, velvety, appressed, sometimes sectored; margin diffuse, hyaline; surface and reverse white. Exudates and soluble pigments absent. Mycelium consisting of hyaline, smooth, septate, branched, hyphae 1.5–3 µm diam.

Conidiophores on MEA macronematous, arising vertically from mycelium, hyaline to pale brown, smooth, cylindrical, thin-walled, dichotomously branched several times, septate, 30–60 × 3–4 µm, or occurring in synnemata, grey to olive brown (4D2–4E3). Synnemata cylindrical to clavate, occurring singly, gregariously, or in clusters joined at base, up to 10 mm high by 1–3 mm diam, surface appearing powdery due to conidia. Conidiogenous cells intercalary and terminal, cylindrical, straight or undulating to geniculate, 7–16(−20) × 3–4 µm, hyaline to pale brown, producing one or more conidia holoblastically from lateral or apical regions, crater-shaped protruding secession scars 1–1.5 × 1–1.5 µm. Conidia pyriform to obovoid, subhyaline to pale brown, (5−)5.5–7(−7.5) × (2.5−)3(−3.5) µm, flattened basal scar indicating former site of attachment to conidiogenous cell.

Stromata upright, solitary, unbranched or occasionally branched once, cylindrical to spathulate or clavate, apices broadly rounded, divided into fertile head and sterile stipe, (2−)2.5−4(−5) × 0.8–1.2 cm including stipes (0.4–1.5 cm high); surface even to irregularly flattened or wrinkled, frequently cracked into a network of light brown to brownish orange (6D4–6D5) angular plates above black basal layer; stromatal interior white; stipes brownish orange to light brown (6D4–6D5) frequently with black longitudinal cracks extending from fertile head; arising from brown (7D7) to black pannose bases, basal mycelia often appearing iridescent. Perithecia immersed, subglobose to globose, 0.3–1 mm diam, lining the perimeter of the stromata. Ostioles conspicuous, papillate, 100–300 µm diam. Asci 95–130 × 6–7 µm, partis sporiferae 50–80 µm, eight-spored, cylindrical, with ascospores arranged uniseriately; apical apparatus inverted hat-shaped, amyloid, 1.5–2 µm long. Ascospores (8−)9–9.5(−10) × (4.5−)5–5.5(−6) µm, dark brown, smooth, unicellular, ellipsoid-inequilateral, narrowly or broadly rounded ends, 1–2 guttules frequently observed, inconspicuous long, straight germ slits which are more or less the spore length, occurring on convex side; small ephemeral cellular appendage 1.5–2 × 1.5 µm, visible on less pigmented immature ascospores and disappearing as spores reach maturity.

Cardinal temperatures: Range 5–30 °C, optimum 20 °C, minimum slightly <5 °C, maximum slightly >30 °C.

Host range: Stromata on decaying hardwood including *Acer*, *Betula*, *Fagus*, and other hardwood trees. Foliar endophyte of *Abies balsamea*, *Picea glauca*, *P. mariana*, *P. rubens*, and *Pinus strobus*. Foliar and stem endophyte of *Vaccinium angustifolium* and *V. corymbosum*. Closely related ITS sequences in GenBank suggest a broad endophytic and endolichenic host range.

Distribution: Eastern Canada and U.S.A.

Additional specimens and cultures examined: DAOM 696463, DAOM 696464, DAOM 696466, DAOM 696480, DAOM 696488, DAOM 696489, DAOM 696492, DAOM 696493, DAOM 696503, NB-699, NB-701, NB-702, NB-703, NB-708, NB-721, NB-722, NB-723, NB-727, NB-746, CH-12, CH-15, CH-16, CH-37, CH-38, CH-4, CH-5, DT-181, DT-6, NB-236-1F, NB-236-2F, NB-236-2I, NB-285-10A, NB-285-10D, NB-285-1A, NB-285-3A, NB-285-6B, NB-285-7A, NB-285-7B, NB-285-7C, NB-285-7D, NB-365-4E, NB-365-71G, NB-365-8A, NB-366-1F, NB-366-2E, NB-366-3L, NB-366-4C, NB-382-1C, NB-382-3A, NB-382-3C, NB-382-3D, NB-382-4B, NB-391-1E, NB-391-2C, NB-391-4C, NB-406-2A, NB-406-2B, NB-406-5A, NB-421-1B, NB-437-5E, NB-464-10A, NB-487-5B, NB-487-5C, NB-487-6A, NB-487-6H, NB-488-6L, NB-505-4D, NB-746, RS9-10E, RS9-12C, T1-3B-2, T1-4B-1, T2-4A-2, T3-2A-2, T3-2B-1, T3-3A-3, T4-3A-1, T5-1A-1, T5-3B-1-1, T6-4B-1, T6-5A-1-2.

Notes: *Xylaria ellisii* is equivalent to *X. corniformis* var. *obovata*, e.g.: Ju *et al*. (2016) recorded blackish-brown ascospores, 8–10.5 × 4.5–5.5(−6) µm from the *X. corniformis* var. *obovata* holotype^53^. The *X. corniformis* species concept is unresolved and consequently the name has been misapplied to various species within the *X. corniformis* and *X. polymorpha* aggregates^45^. The *Xylaria corniformis* aggregate is a polyphyletic morphotaxonomic concept comprising species characterized by stromata with a wrinkled surface and a thin outer layer that gradually cracks into fine scales with maturation, including *X. bipindensis, X. cuneata, X. curta*, *X*. *divisa*, *X. feejeensis*, *X. humosa, X. luteostromata, X. maumeei, X. montagnei*, *X. plebeja*, and *X. rhytidophloea*^45,53–55^. Rogers (1983) noted the taxonomic confusion surrounding *X. corniformis* and its misapplication to *X. bulbosa*, *X. castorea*, *X. curta*, and other morphologically similar species, and recommended that *Xylaria* taxonomy would be best served if the name *X. corniformis* were no longer used^45^. *Xylaria corniformis* s.s. is possibly a rare species known only from Swedish and Polish collections and is characterized by delicate, horn-like stromata with attenuated or sterile apices versus the robust stromata of *X. ellisii*, which also have darker coloured ascospores^44,55,56^. Ju *et al*. (2009) concluded that *X. corniformis* var. *obovata* was an equivalent of *X. corniformis sensu* Læssøe (1987)^55^. Læssøe (1987) noted that *X. corniformis* var. *obovata* was probably the most frequently encountered member of the *X. corniformis* complex in northern temperate regions^44^. Ju *et al*. (2009) considered *X. corniformis* and *X. corniformis* var. *obovata* as distinct species but refrained from making a formal taxonomic decision pending additional evidence^55^. *Xylaria ellisii* is common on decaying fallen *Acer saccharum* branches in New Brunswick during late summer and autumn and is a frequently isolated endophyte of *Picea, Pinus strobus*, and *Vaccinium angustifolium* in Eastern Canada^38^. Conspecific ITS sequences in GenBank suggest that *X. ellisii* is capable of endophytically infecting a wide range of hosts.

## Discussion

*Xylaria ellisii* was the most commonly isolated Xylariaceae endophyte from *Picea* and *Pinus* in Eastern Canada^57^. Stromata of *X. ellisii* were commonly found on decaying *Acer saccharum* branches or stems in the same forest stands where it was isolated as a *Picea* endophyte. Endophyte ITS sequences in GenBank corresponding to *X. ellisii* originate from an exceptional diversity of hosts, including *Tsuga canadensis*, bryophytes (e.g.: *Hypnum* sp.), liverworts (e.g.: *Metzgeria furcata*, *Trichocolea tomentella*), and lichens (e.g.: *Flavoparmelia caperata*, *Sticta beauvoisii*, *Xanthoparmelia conspersa*) (Fig. 3). In New Brunswick, corresponding *X. ellisii* stromata were commonly found in late summer and early fall only on decaying *Acer saccharum* wood; however, the stromatal host range is likely broad. For example, Læssøe (1987) examined European specimens of *Xylaria corniformis* (probably *X. ellisii*) from *Carpinus* and *Fagus*^44^ and Rogers (1983) examined North American collections from *Betula, Fagus*, *Malus*, and *Tsuga*^45^.

*Xylaria ellisii* is a common *Picea* and *Pinus* endophyte even in conifer-dominated stands lacking *Acer saccharum* or any other hardwood hosts possibly suitable for the production of stromata. This indicates that the fungus is capable of persisting in the environment in the prolonged absence of a suitable primary host. The method of transmission between foliage is currently unknown. It is conceivable that the dry, powdery masses of conidia produced from conidiomata *in vitro* are also produced on dead foliage and capable of infecting new foliage by means of air currents or insect vectors^58,59^. Abscised foliage infected with *X. ellisii* is probably capable of saprotrophically colonizing hosts by means of direct contact (viaphytism), as demonstrated in other *Xylaria* species^25^. The known range of hosts that *X. ellisii* can endophytically infect includes lichens and various understory and overstory plant species with different successional statuses, allowing for its persistence across forest succession pathways and disturbances (e.g.: as an endophyte of the fire-adapted seral species *Vaccinium angustifolium*). A proposed endophytic-saprotrophic life history is described and illustrated for *Xylaria ellisii* (as *Xylaria* sp.) by Tanney *et al*.^60^.

The production of the potently antifungal compound griseofulvin by *X. ellisii*, an apparently ubiquitous endophyte with a broad host range, is significant. Griseofulvin is toxic to a wide variety of plant pathogens^61–64^ and is systemically translocated within plants^65^, suggesting that *X. ellisii* endophyte infections could increase host resistance to plant pathogens. For example, Park *et al*. (2005) described griseofulvin production in an unidentified *Xylaria* endophyte of *Abies holophylla* and showed its ability to control the development of plant diseases such as barley powdery mildew (*Blumeria graminis* f. sp. *hordei*), rice sheath blight (*Corticium sasaki*), wheat leaf rust (*Puccinia recondita*), and rice blast (*Magnaporthe grisea*)^64^. Griseofulvin and related compounds are reported from *Xylaria* endophytes of *Asimina triloba*, *Chrysobalanus icaco*, and *Garcinia hombroniana*^66–68^. Richardson *et al*. (2014) reported the production of the antifungal compound griseofulvin by *Xylaria ellisii* (as *Xylaria* sp.) isolated as a foliar endophyte of *Pinus strobus* and *Vaccinium angustifolium*^38^. These isolates produced griseofulvin and its de-halogenated analogue (Fig. 1), along with piliformic acid^38^. Subsequent investigations of white pine seedlings infected with this *Xylaria* species found griseofulvin at biologically effective concentrations in the needles^69^.

Nonribosomal peptides (NRPS) are of great interest as they represent a unique class of natural products with diverse therapeutic applications such as antimicrobial agents (caspofungin, penicillin, vancomycin), anticancer compounds (bleomycin, daptomycin), immunosuppressants (cyclosporine, rapamycin) and as insect toxins (beauvercin, enniatin)^70–73^. This complex structural diversity of linear, cyclic, and cyclic branched architectures is synthesized through a modular enzymatic assembly line process^70,73^. In principle, this enzyme complex is capable of incorporating >500 proteinogenic and nonproteinogenic building blocks, including polyketide and terpene hybrid moieties.

In this study, we have applied a LC-MS metabolomic guided discovery approach to profile the chemical space of a novel endophytic species described here as *Xylaria ellisii*. Our collections of isolates have identical ITS DNA sequences yet differ in their LC-MS metabolite profiles and bioactivity. OPLS-DA and S-plot analysis identified features separated by a statistical toll, Variable Importance in Projection (VIP) scores. VIP scores from the extracted filtrates and mycelium extracts were calculated and extracts differentiated by this method were targeted for compound isolation and structural characterization. This approach resulted in the discovery of three new cyclic pentapeptides given the trivial names ellisiiamides A–C (12–14) and the putative identification and annotation of ellisiiamides D–H by LC-HRMS and LC-HRMS/MS analysis. Additionally, 11 known compounds are reported to be produced by these strains. Ellisiiamide A (**12**) was active against Gram-negative bacteria and is a first report for this scaffold. These findings are of interest as the isolates were also reported from eastern white pine needles in a pine-blueberry forest ecotype. Endophytes from wild *Vaccinium* species may be an interesting source of novel bioactive compounds. This information provides a better understanding of the chemical ecology of plant-fungi microbiomes. In the long term, opportunities may present to employ this information for integrated pest management crop protection strategies.

## Methods

### Sampling, isolation, and culturing

Plant material, including leaves and stems from highbush and wild blueberries, were collected from three different locations within the Acadian forest region of Nova Scotia, Canada. Highbush blueberry endophyte isolates were obtained from a commercial field in Rawdon, Nova Scotia and wild blueberry endophytes isolates were collected from commercial fields in Mount Thom, Debert, and Portapique, Nova Scotia. Specimens were collected in labelled bags and stored at -20 °C prior to fungal isolation. Plant tissues were first washed with sterile deionized water to remove any loose debris and surface contaminants, followed by a chemical surface-sterilization process using sodium hypochlorite bleach (6%) and ethanol (70%). Small segments were then cut and/or incised and placed in Petri plates containing 2% malt extract agar (MEA; 20 g Bacto malt extract, Difco Laboratories, Sparks, USA; 15 g agar, EMD Chemicals Inc., Gibbstown, USA; 1 L deionized H_2_O). Inoculated plates were incubated at 25 °C for 4–8 weeks, depending on the presence of filamentous hyphae. Endophytic fungi that grew from cut ends were then transferred to potato dextrose agar (PDA, Sigma-Aldrich, Canada) plates and incubated at 25 °C.

Field specimens of stromata were collected and stored in paper bags. Single-ascospore isolates were made by affixing with petroleum jelly a small (ca. 5 mm^2^) piece of stroma containing mature perithecia to the lid of a Petri dish containing water agar (WA; 15 g agar, EMD Chemicals Inc., Gibbstown, USA; 1 L deionized H_2_O). Germination of ejected ascospores on the agar surface was confirmed by stereo microscope (Olympus SZX12, Olympus, Tokyo, Japan) and germinating ascospores were transferred to individual Petri plates containing 2% MEA and incubated at 20 °C. Dried specimens were accessioned in the Canadian National Mycological Herbarium (Ottawa, Ont.; DAOM). Living cultures were deposited in the Canadian Collection of Fungal Cultures (Ottawa, Ont.; DAOMC). Additional specimens used for morphological comparison and phylogenetic analyses were also obtained from DAOM, DAOMC, and the personal culture collection of J.B. Tanney.

*Xylaria* strains from highbush blueberry and wild blueberry were cultured in PDB (24 g/L potato dextrose broth) and ML (30 g/L malt) fermentation media. Each strain was grown in 1 L Roux bottles containing 200 mL of media and grown statically for 4–6 weeks at 25 °C. The culture broth was then separated from the mycelium by vacuum filtration using a Whatman #4 filter paper. The filtrate was extracted with equal volumes of ethyl acetate, while the mycelium was first lyophilized for 24 h and then extracted with equivalent volumes of methanol and acetone (1:1). Organic fractions were then dried under reduced pressure by rotary vacuum. Extracts were then re-suspended in 600 μL of HPLC grade acetonitrile with minimal amounts of DMSO added for solubility. The filtrates were then centrifuged at 13,000 rpm for 15 min and Acro-disk (13 mm, 0.45 μm GHP) filtered prior to LC-MS analysis.

### Morphological study

Sections of stromata were cut by hand using a safety razor blade or with a freezing microtome (ca. 15–30 µm thick) and mounted in either water, 5% KOH, 85% lactic acid, or Lugol’s solution with or without 5% KOH pretreatment to test amyloid reactions^74^. Stromata and colony colours were described using alphanumeric codes^75^. Observations of the asexual morph were made from living cultures grown on oatmeal agar (OA)^76^. Microscopic measurements were taken from living material mounted in deionized water and are presented as ranges calculated from the mean ± standard deviation of each measured value with outliers in brackets. Observations were made using an Olympus BX50F4 light microscope and an Olympus SZX12 stereo microscope (Olympus, Tokyo, Japan). Images were captured with an InfinityX-32 camera (Lumenera Corp., Ottawa, Canada) using Infinity Analyze v. 6.5.2 (Lumenera Corp.) software. Photographic plates were assembled using Adobe Photoshop CC 2017.1.1 (Adobe Systems, San Jose, California, USA). Cardinal temperatures were assessed for the type strain (DAOMC 252031) by incubating single-point inoculated Petri dishes containing MEA at 5 °C intervals from 5–40 °C. Each treatment was conducted in triplicate and colony diameters were measured two weeks after inoculation.

### DNA extraction, sequencing, and phylogenetic analyses

DNA was extracted from cultures and stromata using the Ultraclean Microbial DNA Isolation Kit (Mo Bio, Carlsbad, CA) or NucleoSpin Plant II Kit (Macherey-Nagel, Düren, Germany). Stromatal tissue from fresh collections and herbarium specimens underwent an initial grinding stage in liquid nitrogen using an Axygen polypropylene pestle (PES-15-B-SI, Union City, CA, USA).

Loci chosen for sequencing included the internal transcribed spacer rDNA region (ITS), β-tubulin (*BenA*), translation elongation factor 1-alpha (*EF1-α*), the second largest subunit of RNA polymerase II (*RPB2*), 18 s nuc rDNA (SSU), and 28 S nuc rDNA (LSU). Primer pairs used for PCR amplification and sequencing included: ITS1 and ITS4^77^ or ITS4A and ITS5^78^ for ITS; Bt2a and Bt2b for *BenA*^79^; RPB2-5f2 and RPB2-7CR^80^ for *RPB2*; and EF1-728F and EF1-986R^81^ for *EF1-α*. LSU was amplified using LR0R and LR5 and sequenced using the primers LR0R, LR3, LR3R, and LR5^82^. SSU was amplified using the primers NS1 and NS4, and sequenced using the primers NS1, NS2, NS3, and NS4^77^. PCR and sequencing were performed as described by Tanney and Seifert (2017)^57^. To improve ITS amplification in herbarium specimens, 0.5 μm of 20 mg/ml bovine serum albumin (BSA) was added per reaction.

For all analyses, sequences were aligned using MAFFT v7^83^ and visually inspected and manually aligned when necessary in Geneious R8 v8.1.5 (Biomatters, Auckland, New Zealand). The most suitable sequence evolution model was determined based on the optimal Akaike information criterion scores in MrModeltest v2.2.6^84^. Consensus trees were visualized in FigTree 1.4.2 (available at http://tree.bio.ed.ac.uk/software/figtree/) and exported as SVG vector graphics for assembly in Adobe Illustrator v10 (Adobe Systems, San Jose, CA, USA).

Three separate phylogenetic analyses were performed. The first phylogeny included ITS sequences of diverse representative endophytes isolated from highbush and wild blueberry leaves and stem. The ex-type of *Mucor ellipsoideus* (ATCC MYA-4767; NR_111683) was selected as outgroup because of its basal position (Mucoromycotina). Maximum likelihood (ML) analysis was performed using RAxML v8.2.4 in PAUP v4.0b10 starting from a random starting tree with 1000 bootstrap replicates^85,86^.

The second phylogenetic analysis included *RPB2* sequences from related *Xylaria* species. The resulting alignment was 1058 bp long and consisted of 47 taxa, including the *outgroup Barrmaelia rhamnicola* (CBS 142772). Bayesian analysis was performed using MrBayes v3.2.6^87^. Three independent Markov Chain Monte Carlo (MCMC) samplings were performed with 12 chains (11 heated and one cold) with sampling every 500 generations until the standard deviation of split frequencies was <0.01. The first 25% of trees were discarded as burn-in and the remaining trees were kept and combined into one consensus tree with 50% majority rule consensus. Convergence was assessed from the three independent runs using Tracer v1.6^88^. The third phylogenetic analysis included ITS sequences from related endophytic *Xylaria* isolates. The alignment was 593 bp long and included sequences from 107 isolates or samples. The resulting phylogenetic analysis was performed in the same manner as described above, with *Nemania serpens* (GU292820) as the outgroup.

All novel sequences used in this study were accessioned in GenBank (Supplementary Table 13S) and taxonomic novelties and associated metadata were deposited in MycoBank (www.MycoBank.org).

### LC-UV/HRMS and LC-UV/HRMS/MS screening

Extracts of endophytic cultures were screened using a Dionex Ultimate 3000 HPLC-UV system coupled to a Bruker maXis 4 G ultra-high-resolution-qTOF mass spectrometer operated in positive electrospray ionization (ESI) with calibrations done using sodium formate ion clusters. LC-MS data were collected using a scan range of 150–1100 *m/z*, with the nebulizer gas (nitrogen) at 3 bar, dry gas flow at 8 L/min, dry gas temperature at 240 °C, and capillary voltage at 4500 V. Chromatographic separations were performed using a standardized HPLC-UV method with a Supelco Ascentis Express C18 reverse-phase core-shell column (150 × 4.6 mm, 2.7 μm, Sigma Aldrich, USA) operating at 750 μL/min and at 40 °C. UV/vis data were acquired from 190–600 nm and monitored at four wavelengths (210, 254, 275 and 350 nm). Mobile phase composition was linear with a gradient of 5% organic from 0 to 1 min, 5–95% from 1 to 24 min, 95–100% from 24 to 25 min, and 100% from 25 to 31 min. Solvent A was H_2_O + 0.1% formic acid and solvent B was acetonitrile with 0.1% formic acid (v/v). HR-MS/MS analysis was performed on a Thermo Q-Exactive Orbitrap mass spectrometer operated in positive electrospray ionization (ESI+) and coupled to an Agilent 1290 HPLC system.

### Data processing and multivariate statistical analysis

Data processing and analyses were modified from a previously published protocol (Fei *et al*., 2014). Post-acquisition internal calibration using sodium formate clusters in both ESI+ and ESI- were performed with Bruker’s Data Analysis 4.0 SP4. LC-MS data files were converted to.mzXML format using Bruker CompassXport. Metabolic features were extracted and aligned using open source XCMS with centWave algorithm^89^; adducts, isotopic ions, and in-source fragments were identified using CAMERA^90,91^. To acquire the final metabolite feature list, isotopic ions and features with integrated peak area under 10,000 were removed. For mycelium metabolome, metabolite features that eluted after 25 min were eliminated.

Both extracted filtrates and mycelium were analyzed using supervised multivariate OPLS-DA after pareto scaling by SIMCA-P+ 12.0.1 (Umetrics, Kinnelon, NJ). The statistical parameters R^2^X(cum), R^2^Y(cum), and Q^2^(cum) of OPLS-DA were used to assess the fitness of the model. R^2^X (R2Y) indicated the fraction in which metabolite features (X) and group (Y) matrix was were explained by the model. A prediction statistic (Q^2^) above 0.4 was indicative of a statistically robust model, i.e. true differences between the comparing groups, and Q2 between 0.7–1.0 indicated the model was statistically significant^46^. Both R^2^ and Q^2^ followed an upward trend from 0 to 1. For an over-fit model, R^2^ approached 1, and Q^2^ fell toward 0^92^. Significant features between classes were identified based on OPLS-DA S-plot and their Variable Importance in Projection (VIP) score. To ensure the identified metabolites are the sole important markers, the two OPLS-DA analyses were conducted in parallel by only including the significant features or by removing the significant features from the raw data^92^. A useful metabolite subset was produced if the first model was successful and the later model failed.

### Metabolite Isolation and characterization

NMR experiments for 1D and 2D measurements were performed on a Bruker Advance III 700 MHz NMR spectrometer equipped with a 5 mm QNP cryoprobe, operating at 700.17 MHz for ^1^H NMR and 176.08 MHz for ^13^C NMR or a Bruker Advance III HD 850 MhZ NMR spectrometer equipped with a 5 mm TXI probe operating at 850.21 MHz for 1 H NMR and 213.81 MHz for 13 C NMR, with chemical shifts referenced to the residual solvent signal^93^. Nitrogen dried compounds were re-suspended in 200 μL of deuterated solvent (C6D6, CD3OD, or DMSO-*d6*) and transferred to 3 mm NMR tubes (Wilmad 335-pp-7) for NMR measurements. NMR data processing was done using MNOVA NMR software ver. 10.0.1 by Mestrelab Research. Optical rotation measurements were done using an Autopol IV Polarimeter (Rudolph Research Analytical).

Purification of metabolomic targeted metabolites was performed on a semi-preparative HPLC system consisting of an Agilent 1100 series HPLC with a G1311A Quaternary Pump, a G1379A Degasser, a G1367A Wellplate Autosampler, a G1316A Column Thermostat, a G1315B Diode Array Detector (DAD), and a G1364C Automatic Fraction Collector controlled by Agilent ChemStation software (Rev. B.03.02-SR2). Metabolites were isolated using a Phenomenex Synergi-Max reverse-phase C-12 column (250 × 10 mm, 4 μm) (Torrence, CA, USA) operating at 5 mL/min and 40 °C. Mobile phase composition was a linear gradient of 5% organic from 0 to 3 min, 5–30% from 3 to 16 min, 30% from 16 to 20 min, and 30–85% from 20–37 min with fractions collected every 20 s. Known isolated compounds (mg/L): dechlorogriseofulvin (**2**) eluted at 27.1 min (4 mg); griseofulvin (**1**) eluted at 29.1 min (2.8 mg); cytochalasin D (**3**) eluted at 30.2 min (2.5 mg); zygosporin E (**4**) eluted at 32.5 min (2 mg); hirsutatin A (**6**) eluted at 33.9 min (2 mg); and cyclic pentapeptide #1 (**9**) eluted at 34.9 min (4 mg) (Supplementary Figs. 3S and 32–49S)

Newly-isolated compounds (mg/L): ellisiiamide G (**18**) eluted at 31.6 min (0.3 mg); ellisiiamide A (**12**) eluted at 32.8 min (2.0 mg); ellisiiamide B (**13**) eluted at 33.4 min (1.3 mg); and ellisiiamide C (**14**) eluted at 35.6 min (2.3 mg). Compound fractions, from multiple HPLC runs, were pooled together and dried under N_2_ gas in pre-weighed vials prior to NMR and optical rotation measurements (Supplementary Figs. 5–31S, Tables 10–12S).

Ellisiiamide A (**12**) C_30_H_45_N_5_O_5_; white powder; [α]^21^ −86.1 (0.18, MeOH); For ^1^H and ^13^C NMR (DMSO_d6_) spectroscopic data see Supporting Table 9S: HRESIMS (*m/z*) 556.3501 [M+H]^+^ (calcd for C_30_H_46_N_5_O_5,_ 556.3493).

Ellisiiamide B (**13**) C_31_H_47_N_5_O_5_; white powder; [α]^21^ −43.1 (0.04, MeOH); For ^1^H and ^13^C NMR (DMSO_d6_) spectroscopic data see Supporting Table 10S: HRESIMS (*m/z*) 570.3656 [M+H]^+^ (calcd for C_31_H_48_N_5_O_5,_ 570.3650).

Ellisiiamide C (**14**) C_33_H_51_N_5_O_5_; white powder; [α]^21^ −47.8 (0.06, MeOH); For ^1^H and ^13^C NMR (DMSO_d6_) spectroscopic data see Supporting Table 11S: HRESIMS (*m/z*) 598.3968 [M+H]^+^ (calcd for C_33_H_52_N_5_O_5,_ 598.3963).

### Biological activity screening

Compounds were tested for their minimum inhibitory concentration (MIC) according to the Clinical Laboratory Standards Institute (CLSI) protocols M7-A5 and M27-A (National Committee for Clinical Laboratory Standards, 2000, 1997). Stock working solutions were made to 5, 10, and 20 mg/mL and tested at a maximum concentration of 200 μg/mL in 96-well liquid culture (National Committee for Clinical Laboratory Standards, 1997, 2003) as previously described^37^. Preliminary evaluation of biological activity was against *E. coli* BW25113 ΔbamBΔtolC, a membrane and efflux pump compromised strain, *Staphylococcus aureus* ATCC# 29213, *Bacillus subtilis* 1A1, *Micrococcus luteus, Saccharomyces cerevisiae* B4741, and *Candida albicans* ATCC# 90028. A cut-off of <25% growth was used for inhibition, with the trend across dilutions also considered^37^.

## Supplementary information

Supplementary information.
