# Supplementary material for: Metabolomic-guided discovery of cyclic nonribosomal peptides from Xylaria ellisii sp. nov., a leaf and stem endophyte of Vaccinium angustifolium
Source: Sci Rep. 2020 Mar 12;10:4599. doi: 10.1038/s41598-020-61088-x (PMC7067778; doi:10.1038/s41598-020-61088-x)
Supplement: Supplementary file 1 — Supplementary information. [file 41598_2020_61088_MOESM1_ESM.pdf]

Supporting Information

Metabolomic Guided Discovery of Cyclic Nonribosomal Peptides from *Xylaria ellisii* sp. nov., a leaf and stem endophyte of *Vaccinium angustifolium*.

Ashraf Ibrahim[1,*,] Joey Tanney[2,3,4], Fan Fei[1], Keith A. Seifert[4], G. Christopher Cutler[5], Alfredo Capretta[1] , J. David Miller[2], Mark W. Sumarah[2,6]

[1]Department of Chemistry and Chemical Biology, McMaster University, Hamilton, Ontario, L8S 4M1, Canada

[2]Department of Chemistry, Carleton University, Ottawa, Ontario, K1S 5B6, Canada

[3*]Pacific Forestry Centre, Canadian Forest Service, Natural Resources Canada, Victoria, British Columbia, V8Z 1M5, Canada

[4]Ottawa Research and Development Centre, Agriculture and Agri-Food Canada, Ottawa, Ontario K1A 0C6, Canada

[5]Department of Plant, Food, and Environmental Sciences, Faculty of Agriculture, Dalhousie University, Truro, NS B2N 5E3, Canada

[6]London Research and Development Centre, Agriculture and Agri-Food Canada, London, Ontario, N5V 4T3, Canada

* Present address: LifeMine Therapeutics, Cambridge, Massachusetts, 02140, USA

Corresponding author: mark.sumarah@canada.ca (M.W. Sumarah)

Table of Contents

1. Figure 1S. LC-UV/MS Chromatogram of extract E-107 from Malt and PDB media
2. Figure 2S. Supervised OPLS-DA and S-plot of extracted mycelium.
3. Table 1S. Validation parameters of calculated OPLS-DA models for the extracted mycelium.
4. Table 2S. Top 100 metabolite features ranked by VIP scores for the OPLS-DA extracted filtrates .
5. Table 3S. Top 30 metabolite features ranked by VIP scores for the OPLS-DA extracted filtrates in Malt media.
6. Table 4S. Top 50 metabolite features ranked by VIP scores for the OPLS-DA extracted filtrates in PDB media.
7. Table 5S. Top 100 metabolite features ranked by VIP scores for the OPLS-DA extracted mycelium.
8. Table 6S. Top 100 metabolite features ranked by VIP scores for the OPLS-DA extracted mycelium in Malt media.
9. Table 7S. Top 50 metabolite features ranked by VIP scores for the OPLS-DA extracted mycelium in PDB media.
10. Supporting Methods – Identification and characterization of compounds **1-11** and putative identification of cyclic pentapeptides ellisiiamide D-H.
11. Table 8S VIP Scores for compounds **1-19** from OPLS-DA models of extracted filtrates and mycelium of *Xylaria ellisii*.
12. Figure 3S. Q-Exactive MS/MS spectra of known compounds **1-8**.
13. Table 9SA and B HRMS molecular formula of compounds **9-19**, peptide monomer sequence and optical rotation measurements for isolated peptides.
14. Figure 4S. Q-Exactive MS/MS spectra of new and known cyclic pentapeptides **9-19** with key diagnostic ions annotated, and major fragmentation pathways shown.
15. Table 10S. ^{1}H (700 MHz) and ^{13}C (176 MHz) NMR spectroscopic data for ellisiiamide A.
16. Table 11S. ^{1}H (700 MHz) and ^{13}C (176 MHz) NMR spectroscopic data for ellisiiamide B.
17. Table 12S. ^{1}H (700 MHz) and ^{13}C (176 MHz) NMR spectroscopic data for ellisiiamide C.
18. Figure 5S-13S. NMR spectroscopic data of ellissiamide A.
19. Figures 14S-21S. NMR spectroscopic data of ellisiiamide B.
20. Figures 22S-31S. NMR spectroscopic data of ellisiiamide C.
21. Figures 32S-37S. NMR spectroscopic data of cyclic pentapeptide 1.
22. Figures 38S-43S. NMR spectroscopic data of dechlorogriseofulvin.
23. Figures 44S-45S. NMR spectroscopic data of hirsutatin A.
24. Figures 46S-47S. NMR spectroscopic data of cytochalasin D.
25. Figures 48S-49S. NMR spectroscopic data of zygosporin E.
26. Table 13S. Genebank table of sequences generated and used in the phylogenetic analyses in this study.

LC-UV/MS Chromatogram of E-107 from Malt and PDB media at 254 and 210 nm

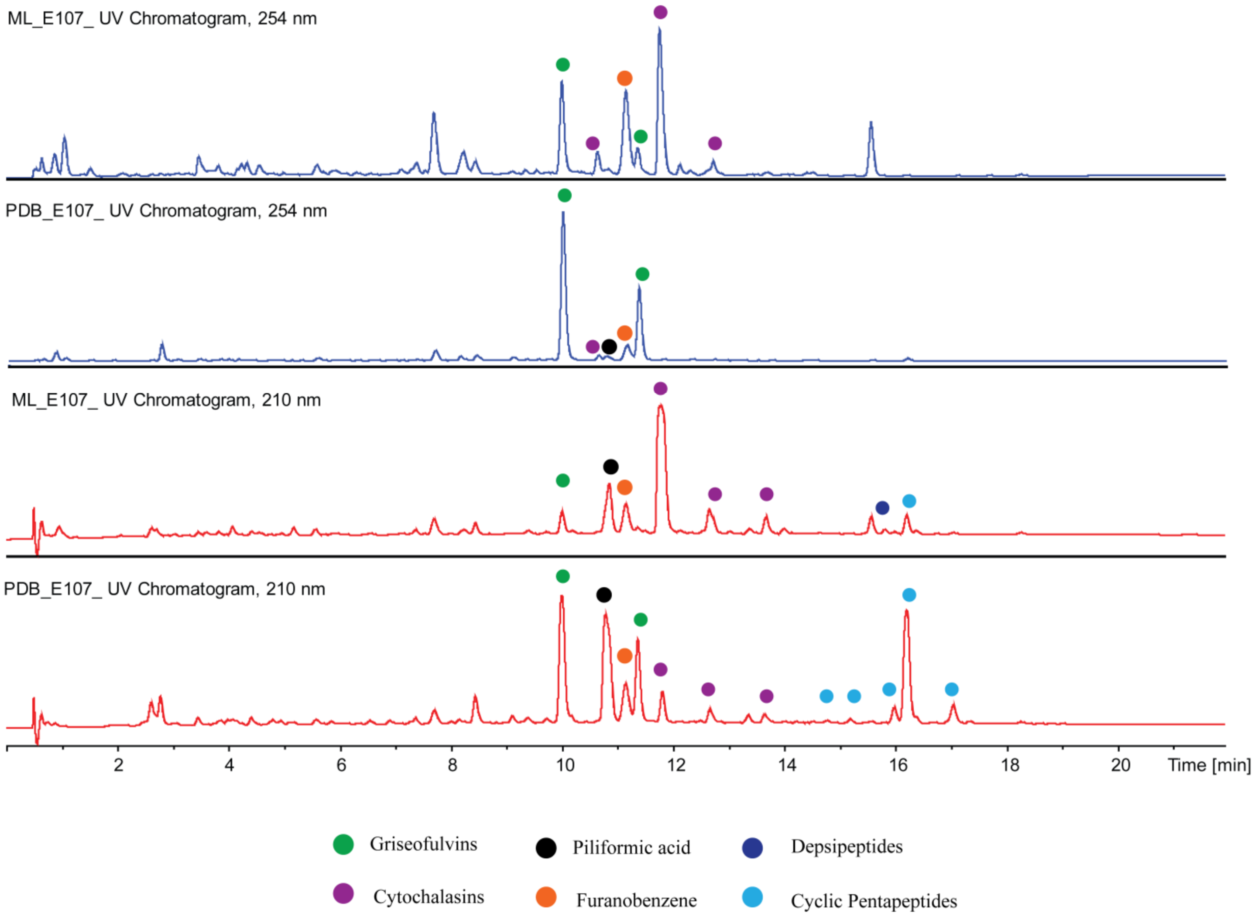

Figure 1S . LC-UV/MS Chromatogram of extract E-107 from Malt and PDB media at 254 and 210 nm respectively, with key metabolites highlighted.

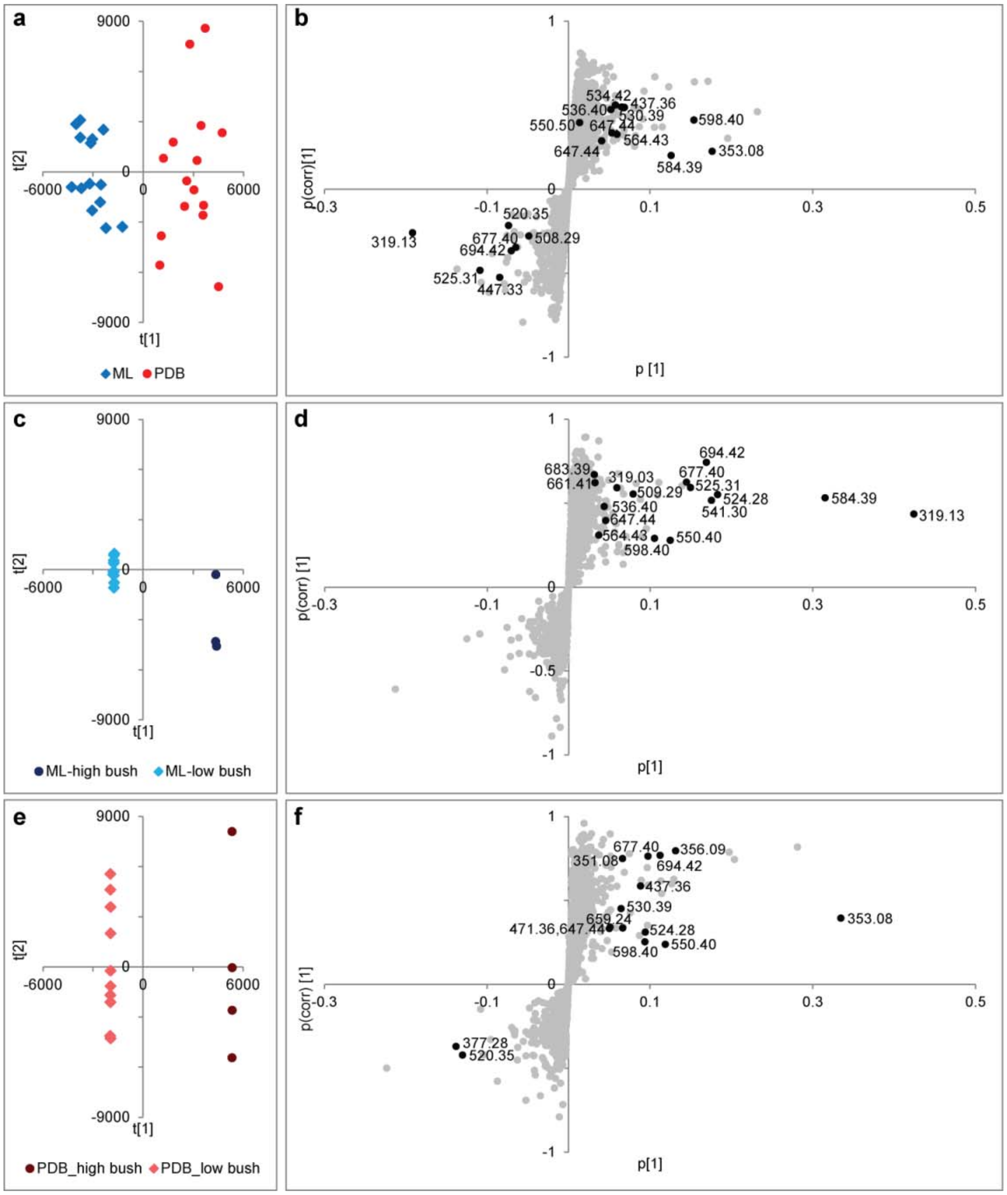

Figure 2S. Supervised multivariate analyses of extracted mycelium of griesofulvin producing *xylaria* endophytes. The OPLS-DA score plot (a) and S-plot (b) is a comparison between endophytes cultured in ML or PDB media. The OPLS-DA score plots and S- plots compares the endophytes isolates from high or low bush blueberries cultured in ML (c, d) or PDB (e, f) medium, respectively.

Table 1S. A summary of validation parameters (R^2X, R^2Y, Q^2) of all calculated OPLS-DA models for the extracted mycelium from the griseofulvin *Xylaria* endophytes isolates from low and high bush blueberries cultured in ML and PDB media. ML-H, endophytes isolated from high bush blueberries cultured in ML medium; ML-L, endophytes isolated from low bush blueberries cultured in ML medium; PDB-H, endophytes isolated from high bush blueberries cultured in PDB medium; PDB-L, endophytes isolated from low bush blueberries cultured in PDB medium.

**Model**	**Variables***	**R^2X(cum)**	**R^2Y(cum)**	**Q^2(cum)**	**Conditions**
**1a**	2645	0.239	0.9	0.689	ML, PDB
**1b**	100	0.292	0.847	0.697	ML, PDB (include top 100 VIP)
**1c**	2545	0.359	0.995	0.743	ML, PDB (exclude top 100 VIP)
**1d**	1945	0.249	0.951	0.347	ML, PDB (exclude top 700 VIP)
**2a**	2645	0.691	1	0.6	ML-H, ML-L
**2b**	100	0.865	1	0.616	ML-H, ML-L (include top 100 VIP)
**2c**	2545	0.0682	0.895	-0.446	ML-H, ML-L (exclude top 100 VIP)
**3a**	2645	0.659	1	0.676	PDB-H, PDB-L
**3b**	50	0.768	0.999	0.836	PDB-H, PDB-L (include top 50 VIP)
**3c**	2595	0.0715	0.89	-0.348	PDB-H, PDB-L (exclude top 50 VIP)

* Number of metabolic features included in the OPLS-DA analyses

Table 2S. The top 100 metabolic features ranked by VIP scores for OPLS-DA analysis of extracted filtrates of griseofulvin producing *Xylaria* endophytes cultured in ML or PDB media. Features highlighted in yellow, indicate metabolites that were identified during this study. Identifications are based on structural characterization of Semi-preparative HPLC or LC-SPE isolated compounds, which were subjected to HRMS measurements, comprehensive high-field 1-2D NMR experiments and/or MS/MS analysis.

Rank	m/z	Rt. (min)	VIP score	Rank	m/z	Rt. (min)	VIP score	Rank	m/z	Rt. (min)	VIP score
1	550.42	15.97	11.37	34	481.32	14.42	4.74	67	1047.62	10.87	3.65
2	584.41	16.2	11.1	35	487.35	8.92	4.74	68	263.11	6.43	3.62
3	261.13	2.62	10.64	36	557.29	10	4.68	69	487.35	13.17	3.61
4	239.18	8.46	9.78	37	1032.58	11.81	4.66	70	551.5	15.97	3.6
5	320.16	10.01	8.07	38	522.37	14.47	4.6	71	437.35	15.42	3.57
6	508.28	12.56	8.02	39	267.12	7	4.55	72	560.43	12.58	3.55
7	498.35	14.44	7.91	40	982.77	13.9	4.49	73	445.3	14.72	3.41
8	167.11	3.41	7.71	41	441.31	11.21	4.44	74	353.13	11.37	3.41
9	320.1	9.98	7.69	42	333.21	8.81	4.42	75	253.11	5.04	3.4
10	543.39	13.03	7.25	43	978.69	14.42	4.41	76	245.13	4.83	3.31
11	430.24	11.81	7.18	44	491.27	11.81	4.41	77	405.19	5.6	3.25
12	490.31	11.81	7.1	45	482.36	15.57	4.35	78	598.5	17.04	3.23
13	169.13	10.77	7.09	46	455.36	12.33	4.28	79	509.28	11.83	3.18
14	678.39	15.82	6.98	47	532.36	10.42	4.25	80	929.67	15.58	3.18
15	463.31	14.45	6.91	48	1000.62	13.69	4.23	81	305.11	7.28	3.17
16	197.13	2.72	6.74	49	647.43	13.11	4.22	82	661.39	17.24	3.16
17	207.16	15.59	6.69	50	317.22	14.71	4.2	83	439.36	13.35	3.09
18	197.12	10.77	6.53	51	447.32	15.57	4.18	84	169.11	3.51	2.99
19	600.4	14	6.46	52	706.2	11.37	4.15	85	237.15	7.13	2.96
20	524.29	10.87	5.82	53	946.77	15.59	4.14	86	561.41	9.59	2.93
21	208.11	4.25	5.61	54	309.14	6.82	4.07	87	240.16	8.46	2.92
22	564.43	16.89	5.59	55	207.1	3.47	4	88	1064.59	10.87	2.91
23	151.12	10.77	5.47	56	413.28	14.54	3.98	89	525.3	13.97	2.91
24	356.09	11.37	5.28	57	933.74	16.39	3.92	90	566.4	13.65	2.9
25	544.4	12.56	5.27	58	193.09	6.33	3.92	91	540.27	10	2.88
26	695.42	15.82	5.24	59	535.41	8.87	3.87	92	1014.69	11.89	2.86
27	485.33	9.33	5.16	60	355.12	11.36	3.86	93	321.13	10.01	2.85
28	1015.59	11.86	5.11	61	489.37	12.5	3.83	94	536.39	14.22	2.81
29	947.7	15.58	5.1	62	525.3	11.8	3.76	95	469.34	13.24	2.79
30	429.31	15.57	4.88	63	647.43	15.03	3.76	96	487.35	11.61	2.78
31	423.3	11.21	4.87	64	585.53	16.2	3.75	97	431.32	16.39	2.77
32	209.1	3.69	4.86	65	675.69	24.17	3.73	98	456.28	11.2	2.73
33	705.23	11.37	4.81	66	556.36	14.76	3.72	99	239.13	2.78	2.72
								100	203.18	8.92	2.71

Table 3S. The top 30 metabolic features ranked by VIP scores for OPLS-DA analysis of extracted filtrates of griesofulvin *Xylaria* endophyte isolate from low or high bush blueberries cultured in ML medium Features highlighted in yellow, indicate metabolites that were identified during this study. Identifications are based on structural characterization of Semi-preparative HPLC or LC-SPE isolated compounds, which were subjected to HRMS measurements, comprehensive high-field 1-2D NMR experiments and/or MS/MS analysis

Rank	m/z	Rt. (min)	VIP score
1	239.18	8.46	13.02
2	598.41	17.04	11.59
3	221.16	8.84	10.74
4	550.42	15.97	10.42
5	490.31	11.81	9.45
6	508.28	12.56	9.21
7	1032.58	11.81	8.26
8	678.39	15.82	8.24
9	319.16	10.01	8.12
10	1015.59	11.86	8.05
11	473.29	9.71	7.73
12	320.1	9.98	7.01
13	570.37	15.19	6.87
14	279.2	8.84	6.81
15	1016.62	11.81	6.69
16	203.18	8.92	6.53
17	309.14	6.82	6.51
18	430.24	11.81	6.43
19	267.12	7	5.9
20	354.1	11.37	5.66
21	240.1	8.45	5.45
22	584.41	16.2	5.36
23	305.11	7.28	5.26
24	295.17	7.03	5.22
25	675.69	24.17	5.13
26	219.18	6.96	5.05
27	173.1	7.38	4.93
28	491.27	11.81	4.75
29	237.12	5.52	4.73
30	263.11	6.43	4.63

Table 4S. The top 50 metabolic features ranked by VIP scores for OPLS-DA analysis of extracted filtrates of griesofulvin *Xylaria* endophytes isolated from low or high bush blueberries cultured in PDB medium. Features highlighted in yellow, indicate metabolites that were identified during this study. Identifications are based on structural characterization of Semi-preparative HPLC or LC-SPE isolated compounds, which were subjected to HRMS measurements, comprehensive high-field 1-2D NMR experiments and/or MS/MS analysis.

Rank	m/z	Rt. (min)	VIP score	Rank	m/z	Rt. (min)	VIP score
1	320.16	10.01	20.43	26	490.31	11.81	4.98
2	706.2	11.37	10.84	27	707.17	11.37	4.97
3	221.16	8.84	9.89	28	1048.6	10.89	4.88
4	598.41	17.04	9.49	29	203.18	8.92	4.72
5	356.09	11.37	8.66	30	525.3	11.8	4.65
6	353.13	11.37	8.53	31	265.19	13.26	4.58
7	541.3	10.87	8.02	32	393.3	24.07	4.47
8	321.13	10.01	7.9	33	705.23	11.37	4.36
9	181.05	10.01	7.35	34	320.1	9.98	4.33
10	1015.59	11.86	7.28	35	209.12	10.92	4.32
11	659.23	10.01	7.13	36	1047.62	10.87	4.23
12	550.42	15.97	6.51	37	527.39	14.24	4.22
13	678.39	15.82	6.26	38	763.66	24.04	4.22
14	223.17	13.79	6.16	39	570.37	15.19	4.21
15	584.41	16.2	6.09	40	456.28	11.2	4.13
16	251.11	10	6.02	41	317.11	9.56	4.07
17	473.29	9.71	5.95	42	1088.82	14.21	4.06
18	219.18	6.96	5.79	43	694.47	15.82	4.01
19	351.07	11.6	5.65	44	391.29	24.1	3.98
20	675.69	24.17	5.44	45	585.41	16.2	3.89
21	279.2	8.84	5.36	46	293.18	6.41	3.88
22	647.43	13.11	5.08	47	557.29	10	3.87
23	522.37	14.47	5.07	48	247.17	13.36	3.86
24	165.08	10	5	49	237.11	10.84	3.7
25	193.09	6.33	5	50	459.32	12.04	3.67

Table 5S. The 100 metabolic features ranked by VIP scores for OPLS-DA analysis of extracted mycelium of griesofulvin *Xylaria* endophytes cultured in ML or PDB media. Features highlighted in yellow, indicate metabolites that were identified during this study. Identifications are based on structural characterization of Semi-preparative HPLC or LC-SPE isolated compounds, which were subjected to HRMS measurements, comprehensive high-field 1-2D NMR experiments and/or MS/MS analysis

Rank	m/z	Rt. (min)	VIP score	Rank	m/z	Rt. (min)	VIP score	Rank	m/z	Rt. (min)	VIP score
1	550.4	16.03	11.98	34	520.35	16.46	3.45	67	205.07	0.52	2.79
2	353.08	11.42	10.77	35	758.6	23.31	3.38	68	169.13	10.83	2.75
3	666.54	24.7	8.06	36	677.4	15.85	3.35	69	754.65	28.53	2.72
4	761.64	28.66	7.91	37	294.16	0.76	3.32	70	251.16	0.84	2.71
5	319.13	10.05	7.78	38	764.67	25.16	3.31	71	441.31	12.71	2.69
6	598.4	17.08	7.17	39	1032.59	11.86	3.29	72	489.38	12.55	2.69
7	682.54	23.84	7.07	40	217.1	2.63	3.26	73	509.29	11.85	2.68
8	933.69	16.41	6.65	41	500.41	18.31	3.25	74	734.62	28.84	2.63
9	205.1	1.77	5.69	42	490.28	12.25	3.23	75	514.36	14.43	2.62
10	584.39	16.24	5.6	43	768.63	24.15	3.23	76	708.61	28.54	2.62
11	698.55	22.63	5.54	44	705.26	11.42	3.22	77	536.4	14.28	2.6
12	223.17	13.82	5.44	45	782.59	22.33	3.22	78	489.32	9.25	2.58
13	760.63	28.95	5.35	46	687.51	23.86	3.19	79	437.36	17.53	2.57
14	235.13	0.65	5.24	47	647.44	13.16	3.17	80	768.59	23.74	2.57
15	353.28	17.13	4.82	48	437.36	15.36	3.14	81	437.36	20.84	2.55
16	525.31	11.86	4.71	49	229.16	1.88	3.14	82	231.24	2.01	2.54
17	413.31	16.41	4.57	50	317.22	14.75	3.12	83	407.3	13.71	2.52
18	355.14	11.42	4.54	51	478.31	16.25	3.09	84	647.44	16.1	2.52
19	245.19	3.15	4.5	52	274.1	0.67	3.05	85	193.09	11.19	2.48
20	707.17	11.42	4.42	53	455.37	15.35	3.04	86	508.29	12.68	2.47
21	488.21	2.11	4.39	54	377.28	20.33	3.02	87	752.63	27.38	2.47
22	671.51	24.74	4.33	55	455.36	12.4	3.02	88	665.53	23.86	2.46
23	425.31	13.72	4.33	56	740.55	22.81	2.99	89	169.13	3.55	2.45
24	548.39	10.36	4.28	57	471.36	14.26	2.96	90	193.09	6.58	2.38
25	457.38	14.79	4.17	58	344.29	18.88	2.95	91	983.66	13.93	2.36
26	320.14	10.05	4.08	59	530.39	12.27	2.95	92	736.61	25.13	2.35
27	430.25	11.85	3.94	60	783.6	21.79	2.89	93	474.4	18.15	2.34
28	694.42	15.85	3.9	61	782.62	29.01	2.86	94	705.52	21.97	2.32
29	217.09	0.63	3.88	62	166.13	1.01	2.86	95	151.12	10.83	2.31
30	447.33	13.93	3.83	63	184.14	0.73	2.85	96	549.4	10.04	2.3
31	784.61	22.69	3.75	64	762.64	28.02	2.84	97	279.14	4.13	2.3
32	431.33	16.42	3.67	65	564.43	16.96	2.83	98	338.35	24.21	2.29
33	439.37	13.39	3.55	66	534.42	13.07	2.81	99	356.09	11.42	2.29
								100	642.56	25.17	2.28

Table 6S. The 100 metabolic features ranked by VIP scores for OPLS-DA analysis of extracted mycelium of griesofulvin *Xylaria* endophytes isolated from low or high bush blueberries cultured in ML medium. Features highlighted in yellow, indicate metabolites that were identified during this study. Identifications are based on structural characterization of Semi-preparative HPLC or LC-SPE isolated compounds, which were subjected to HRMS measurements, comprehensive high-field 1-2D NMR experiments and/or MS/MS analysis.

Rank	m/z	Rt. (min)	VIP score	Rank	m/z	Rt. (min)	VIP score	Rank	m/z	Rt. (min)	VIP score
1	319.13	10.05	21.82	34	493.3	13.72	3.14	67	279.2	8.9	2.12
2	584.39	16.24	16.24	35	144.99	27.6	3.14	68	146.99	25.81	2.1
3	933.69	16.41	10.93	36	637.25	10.06	3.08	69	249.23	16.9	2.09
4	524.28	10.92	9.43	37	319.03	12.57	3.05	70	395.34	20.65	2.07
5	541.3	10.91	9.05	38	706.19	11.42	2.99	71	204.13	0.71	2.07
6	694.42	15.85	8.7	39	740.55	22.81	2.98	72	317.07	9.61	2.06
7	525.31	11.86	7.7	40	206.08	0.55	2.92	73	727.16	11.42	2.01
8	677.4	15.85	7.42	41	982.72	13.94	2.81	74	1049.58	11.36	2
9	1064.58	10.92	7.18	42	223.17	13.82	2.54	75	551.42	20.18	1.97
10	550.4	16.03	6.44	43	671.51	24.74	2.53	76	172.1	0.88	1.96
11	783.6	21.79	6.4	44	282.28	21.55	2.48	77	321.02	12.56	1.93
12	355.14	11.42	5.72	45	740.65	22.69	2.47	78	564.43	16.96	1.92
13	166.13	1.01	5.65	46	528.25	3.72	2.46	79	263.1	6.35	1.91
14	1032.59	11.86	5.63	47	760.63	28.95	2.46	80	358.18	15.85	1.91
15	598.4	17.08	5.45	48	146.98	28.37	2.44	81	254.16	0.91	1.89
16	490.28	12.25	4.93	49	224.14	13.81	2.38	82	184.14	0.73	1.89
17	705.26	11.42	4.61	50	335.04	10.02	2.36	83	166.06	1.02	1.88
18	430.25	11.85	4.55	51	320.14	10.05	2.35	84	431.33	16.42	1.87
19	1047.56	10.92	4.28	52	647.44	16.1	2.34	85	295.2	7.07	1.86
20	509.29	11.85	4.08	53	377.33	25.07	2.33	86	175.06	0.54	1.86
21	339.42	24.21	4.05	54	413.31	16.41	2.29	87	1005.57	13.72	1.84
22	758.6	23.31	3.87	55	1000.61	13.71	2.28	88	786.63	23.76	1.84
23	318.31	14.52	3.72	56	277.22	12	2.27	89	184.07	0.59	1.84
24	785.61	22.96	3.66	57	536.4	14.28	2.26	90	699.39	15.87	1.83
25	205.1	1.77	3.64	58	437.36	17.53	2.25	91	207.1	8.26	1.83
26	707.17	11.42	3.62	59	666.54	24.7	2.24	92	237.12	10.85	1.8
27	782.59	22.33	3.5	60	356.09	11.42	2.24	93	203.19	9.36	1.78
28	267.1	7.03	3.46	61	169.13	3.55	2.21	94	268.11	0.72	1.75
29	377.28	20.33	3.42	62	478.31	16.25	2.19	95	355.3	20.12	1.74
30	526.31	11.58	3.28	63	457.31	10.22	2.19	96	1069.54	10.92	1.74
31	239.14	8.49	3.18	64	1015.57	11.86	2.17	97	687.51	23.86	1.7
32	659.24	10.06	3.17	65	804.6	22.12	2.17	98	491.32	12.25	1.7
33	716.55	24.94	3.15	66	771.61	24.08	2.15	99	231.24	2.01	1.7
								100	151.12	3.16	1.69

Table 7S. The top 50 metabolic features ranked by VIP scores for OPLS-DA analysis of extracted mycelium of griesofulvin *Xylaria* endophytes isolated from low or high bush blueberries cultured in PDB medium.

Rank	m/z	Rt. (min)	VIP score	Rank	m/z	Rt. (min)	VIP score
1	353.08	11.42	17.22	26	184.07	0.59	4.5
2	707.17	11.42	14.48	27	760.63	28.95	4.49
3	782.59	22.33	11.48	28	1064.58	10.92	3.92
4	666.54	24.7	10.49	29	727.16	11.42	3.86
5	682.54	23.84	10.16	30	338.35	24.21	3.58
6	377.28	20.33	7.1	31	181.05	10.05	3.53
7	356.09	11.42	6.78	32	457.38	14.79	3.46
8	520.35	16.46	6.68	33	353.28	17.13	3.44
9	706.19	11.42	6.66	34	351.08	11.65	3.42
10	768.59	23.74	6.54	35	659.24	10.06	3.42
11	550.4	16.03	6.14	36	254.16	0.91	3.39
12	698.55	22.63	5.89	37	764.67	25.16	3.38
13	489.32	9.25	5.85	38	530.39	12.27	3.33
14	694.42	15.85	5.79	39	804.6	22.12	3.22
15	761.64	28.66	5.55	40	320.14	10.05	3.2
16	758.6	23.31	5.38	41	642.56	25.17	3.07
17	671.51	24.74	5.04	42	639.33	10.06	3
18	677.4	15.85	5.03	43	514.36	14.43	2.85
19	541.3	10.91	5	44	488.21	2.11	2.74
20	490.28	12.25	4.98	45	925.75	28.42	2.73
21	783.6	21.79	4.9	46	375.07	11.42	2.72
22	524.28	10.92	4.86	47	353.09	12.9	2.71
23	598.4	17.08	4.84	48	251.1	10.05	2.69
24	341.11	10.07	4.61	49	339.42	24.21	2.69
25	437.36	15.36	4.56	50	217.09	0.63	2.68

Supporting Methods

Metabolite Identification of known compounds 1-11

Compounds (**1**-**2**) showed high VIP values (4.6 - 11.42 and 3.2 - 20.43) across the extracted filtrates and mycelium OPLS-DA models. Examination of the LC-HRMS and LC-HRMS/MS spectra revealed protonated molecular ions at *m/z* 353 and *m/z* 319, with diagnostic daughter ions (at *m/z* 285, 215, 165, 69 and 251, 181, 165, 69 respectively), indicative of the potent antifungal agent griseofulvin and dechlorogriseofulvin (Figure 4, Figure S3 and S38-43). Comparison of the 1D and 2D NMR measurements of compound (**2**) to the literature further validated the identifications (Kimura et al., 1992).

Compound (**3**) showed high VIP values between 4.98 – 7.18 and was predominantly produced from lowbush isolates grown in ML media. The HRMS spectrum showed a protonated molecular ion at *m/z* 508, giving a molecular formula of $C_{30}H_{38}NO_6$ with thirteen degrees of unsaturation. Dereplication screening using the molecular formula *via* Antibase revealed a total of eight potential cytochalasin compounds. Cytochalasins are a unique class of toxic secondary metabolites that are common to the *Xylariaceae* family (Whalley and Edwards, 1995), with over 70 reported analogues in Antibase alone. They are known inhibitors of actin polymerization, affecting cell division, and as plant growth regulators (Casella et al., 1981; Cox et al., 1983; Evidente et al., 1990). Key MS/MS fragment ions at *m/z* 490, 430, 265, 120 and supporting 1D and 2D NMR (^{1}H, ^{13}C, COSY, HSQC, and HMBC) assignments confirmed the structure as cytochalasin D (Figure 4, S3 and S46-47.). Examination of the molecular formulas and LC-HRMS/MS fragmentation of compounds (**4**) (m/z 492) and (**5**) (m/z 524), indicating possible analogues compounds to Cytochalasin D. The 1D and 2D NMR and MS/MS

analysis of compounds (**4**) and (**5**) identified them as Zygosporin E (*m/z* 492) (Figures S48-49).and epoxycytochalasin D *(m/z* 524) (Figures 3S and 48-49S).

Compound (**6**) had protonated molecular ion of m/z 677 with VIP values from 3.35- 7.42, and was predominantly found in the intercellular metabolome from both ML and PDB media. Database screening returned no matches from Norine, and only one potential candidate from Antibase, that of hirsutatin A, a cyclic hexadepsipeptide isolated from the insect pathogenic fungus *Hirsutella nivee* (Isaka et al., 2005). The ^{1}H spectrum of compound (**6**) in C_6D_6 revealed the presence of key diagnostic amide protons (δ 8.50 [d, *J* =6.5 Hz], δ 7.93 [d, *J*=8.8 Hz], δ 6.75 [d, *J* =9.0 Hz]) and one N-methyl-amide signal (δ 2.58 [3H,s]), including six α-protons, which supported the hirsutatin peptide scaffold (Figure 3S and 44-45S). Furthermore, the ^{13}C NMR and multiplicity edited HSQC spectrums revealed a total of 34 carbon atoms, with six resonances attributed to carbonyls (δ 172.7, 171.9, 171.8, 170.5, 169.7 and 168.0 ppm), which is consistent with the literature assignments (Isaka et al., 2005).

Compound (**7**) was established to be piliformic acid and based on the protonated molecular ion of *m/z* 215 and key diagnostic MS/MS fragments of *m/z* 197, 169, 241, 120, that are in agreement with the literature (Mangaleswaran and Argade, 2000) (Figure 3S).

Compound (**8**) was identified as 2, 3-dihydro-2,4-dimethylbenzofuran-7-carboxylic acid, a furanobenzene metabolite recently reported from *Xylaria* along with compounds (**1**) and (**7**) (Richardson et al., 2014). The furanobenzene metabolite had a protonated molecular ion at *m/z* 193 and diagnostic MS/MS fragments at *m/z* 175, 165, 147 and 91(Figure 3S).

The HRSMS spectra for Compound (**9**) showed a protonated molecular ion at *m/z* 584, affording a molecular formula of $C_{32}H_{49}N_5O_5$, with VIP values of 5.36-16.24. Compound (**9**) is found in

both the extracted filtrates and mycelium. Database screening of the HRMS molecular formula identified only one candidate, cyclic pentapeptide 1, *cyclo*-(-NMePhe-Pro- Leu-Ile-Val-) (Wu et al., 2011). Isolation and characterization of compound (**9**) by 1D and 2D NMR as well as MS/MS analysis confirmed the identify of cyclic pentapeptide 1 (Figure 4S and 32-37S). Examination of the ^{1}H and ^{13}C NMR spectra revealed the presence of five α protons (δ5.24/61.0, 5.04/58.8, 4.86/49.0, 4.25/57.8 and 4.01/59.9 ppm) and their associated carbonyl resonances (174.4, 173.2, 172.5, 172.4, and 171.5 ppm). Analysis of the MS/MS spectra of (**9**) further validated the identification (Figure 4, Figure 4BS). Cyclic peptides in general, can generate a number of direct-sequence and non-direct sequence fragment ions when subjected to collision induced dissociation (CID) within a mass spectrometer. These fragment ions can arise from multiple ring opening events along the peptide back-bone, with cleavage sites at the amide bonds (Eckart, 1994; Liu et al., 2009). In the case of cyclic pentapeptide 1, the presence of the N-methylated phenylalanine (N-MePhe) and the presence of a basic residue (Pro), created two main fragmentation pathways with ring opening events at the (1) N-MePhe- Pro and (2) Pro-Leu amide bonds (Figure 4, Figure 4BS). Diagnostic MS/MS fragments for (**9**) at *m/z* 487.3, 374.2, 261.2 and 162.1 support the main N-MePhe-Pro fragmentation pathway, while the minor Pro-Leu pathway is evident with diagnostic fragments at *m/z* 471.3, 358.2, 261.2 and 162.1. The optical rotation for cyclic pentapeptide 1 was measured to be $[\alpha]^{21}$ -63.4 (0.18, MeOH), which is in-line with values reported by Wu et al., (2011) of $[\alpha]^{25}$ -46.8 (1.36, MeOH) and ellissiamides A-C at $[\alpha]^{21}$ -86.1 (0.06, MeOH), $[\alpha]^{21}$ -43.1 (0.04, MeOH), and $[\alpha]^{20}$ -47.8 (0.06, MeOH) respectively (Supporting Table 9SA and 9SB).

Compound (**10**) was identified as xylarotide A based on the protonated molecular ion at *m/z* 550 m/z and diagnostic MS/MS fragments (*m/z* 453.3, 340.3, 227.3, 128.1). Xylarotide is a related

cyclic pentapeptide with an N-MeLeu substituted for N-MePhe (Li et al., 2011) with a reported optical rotation of [α]d -3.6 (c 0.18, CHCl3). Xylarotide was present in all extracted filtrates and mycelium models, and found in both low and high bush blueberry extracts with VIP scores of 6.51-11.53 (Figure 4AS).

Compounds (**11**) was identified as cyclic pentapeptide 2 with a protonated molecular ion of *m/z* 536 and diagnostic MS/MS fragments in accordance with literature values (*m/z* 423.3, 326.2, 213.2, 100.1) (Figure 4AS). Cyclic pentapeptide 2 had VIP scores of 2.26-2.81 and is structurally similar to cyclic pentapeptide 1 and xylariotide A with Leu substituted for N-MePhe and N-MeLeu respectively (Wu et al., 2011).

Metabolomic-guided discovery and putative characterization of ellisiiamides D-H Ellisiiamide D (**15**) (Rt ~ 14.47min) was identified in the PDB extracted filtrates of highbush blueberry extracts with VIP scores of 4.6-5.07. The molecular formula was determined to be $C_{27}H_{47}N_5O_5$ with 7 double bond equivalents based on the protonated molecular ion of *m/z* 522. Analysis of the MS/MS spectra revealed the two main fragmentation pathways with diagnostic fragments of *m/z* 409.3 (-Leu), 312.2 (-Pro), 199.1 (-Leu), 100.1 (-Val) indicating ring-opening events at Val-Leu, and fragments at *m/z* 425.3 (-Pro), 312.2 (-Leu) indicating a ring-opening event at Leu-Val Figure 2S). Compound **15** is analogous to cyclic pentapeptide 2 with Val substituting for IsoLeu at position #3 (Figure 4, 4AS, Table 1 and 10S) confirming a sequence of cyclo-(Leu-Val-Val-Leu-Pro).

Ellisiiamide E (**16**) (Rt ~16.89) was identified in the extracted filtrates and mycelium and primarily in lowbush blueberry extracts with VIP scores of 1.92-5.59. The protonated molecular ion was *m/z* 564 and the molecular formula was established to be $C_{30}H_{54}N_5O_5$ with 7 double bond equivalents. MS/MS analysis of the tandem MS spectra

revealed key fragment ions at m/z 467.4 (-Pro), 354.3 (-Leu), 241.2 (-IsoLeu), 128.1 (-Isoleu/Leu) and at *m/z* 451.3 (-Leu), 338.2 (-IsoLeu) and 225.2 (-IsoLeu) indicating ring-opening events at N-MeLeu-Pro and Pro-Leu, consistent with the cyclic pentapeptide scaffold. Compound **16** is structurally similar to cyclic pentapeptide 2, with an N-MeLeu substituted for Leu at position 1 and Iso/Leu substituted at position 2 (Figure 4, 4AS, Table 1 and 10S).

Ellisiiamide F (**17**) was identified based on the protonated molecular ion at *m/z* 586 affording a molecular formula of $C_{31}H_{47}N_5O_6$ with 11 double bond equivalents and comparative LC-HRMS/MS analysis to elissiamide B (**13**). Examination of the molecular formula of **13** and **17** indicated a difference of a single oxygen atom, with no change in the degrees of unsaturation. Comparative analysis of the MS/MS tandem spectra of **17** revealed diagnostic fragments for ring-opening at N-MePhe-Pro+16 of *m/z* 473.3 (-Pro+16), 360.2 (-Leu), 261.2 (-Val) and 162.1 (-Val) and ring-opening at Pro+16-Leu at *m/z* 473.3 (-Leu), 374.2 (-Val), and 275.1 (-Val). This fragmentation pattern is consistent with the cyclic pentapeptide scaffold, and similar to (**13**), indicative of a hydroxyl-proline moiety at position 5. This is further supported by the earlier, more polar elution time of **17** vs **13**, Rt ~14.11 min vs ~15.19 min using reverse-phase C-18 column (Figure 4, 4AS, Table 1 and 10S).

Ellisiiamide G (**18**) had a protonated molecular ion of *m/z* 600, affording a molecular formula of $C_{32}H_{51}N_5O_6$ with 11 double bond equivalents and differing in only a single oxygen atom as compared to **9**. Ellisiamide G was predominately found in the extracellular metabolome with a VIP score of 6.46. LC-HRMS/MS analysis of **18** revealed a similar fragmentation pattern to **9** with diagnostic fragments at *m/z* 487.3 (-Pro+16), 374.2 (-Leu), 261(-IsoLeu), 162.1 (-Val) indicating a hydroxyl proline moiety. Examination of the elution times of **18** and **9** reveals

Rt~14.00 vs ~16.2min respectively, supporting the presence of a hydroxyl addition at position #5 (Figure 4, 4AS, Table 1 and 10S).

Ellisiiamide H (**19**) was identified as another new hydroxy proline substituted cyclic pentapeptide. The LC-HRMS spectra of **19** afforded a protonated molecular ion o f m/z 614, affording a molecular formula of $C_{33}H_{51}N_5O_6$ with 11 double bond equivalents and differing in a single oxygen atom as compared to elisiamide C. MS/MS analysis of the tandem MS spectra showed diagnostic ions at *m/z* 501.3 (-Pro+16), 388.2 (-Leu), 275.2 (-IsoLeu), 162.1 (-IsoLeu) with only one main fragmentation pathway visible, indicating ring-opening at N-MePhe-Pro+16. Similar to above, compound **19** eluted earlier than **14** at Rt~14.89 vs ~17.04 respectively (Figure 4, 4AS, Table 2 and 9S).

Table 8S. TOP VIP scores for compounds **1-19** from OPLS-DA validation models of extracted filtrates and mycelium of Low and High bush isolates of griseofulvin producing *Xylaria ellisii*.

#	Compound	Class	VIP Scores - Filtrates*			VIP Scores - Mycelium*		
			1b	2b-ML	3b-PDB	1b	2b- ML	3b-PDB
				Low vs High Bush			Low vs High Bush	
	**Known**							
1	Griseofulvin	PKS	4.81	5.66	8.53	10.77	5.72	17.22
2	Dechlorogriseofulvin	PKS	8.07	8.12	20.43	7.78	21.82	3.20
3	Cytochalasin D	PKS-NRPS	7.18	9.45	7.28	3.94	4.55	/
4	Zygosporin E	PKS-NRPS	/	/	/	2.36	2.81	/
5	Epoxycytochalasin D	PKS-NRPS	5.82	/	4.23	/	9.43	4.86
6	Hirsutain A	NRPS	/	/	4.01	3.90	8.70	5.79
7	Piliformic acid	PKS	6.53	/	3.70	2.75	1.80	
8	2,3-dihydro,2,4-dimethylbenzofuran -7-carboxylic acid	PKS	/	/	/	2.48	/	/
9	Cyclic Pentapeptide #1	NRPS	11.10	5.36	6.09	5.60	16.24	/
10	Xylarotide A	NRPS	11.37	10.52	6.51	11.98	6.44	6.14
11	Cyclic Pentapeptide #2	NRPS	2.81	/	/	2.60	2.26	/
	**New**							
12	Ellisiiamide A	NRPS	3.72	/	/	2.60	/	/
13	Ellisiiamide B	NRPS	/	6.87	4.21	/	/	/
14	Ellisiiamide C	NRPS	3.23	11.59	/	7.17	5.45	4.84
15	Ellisiiamide D	NRPS	4.60	/	5.07	/	/	/
16	Ellisiiamide E	NRPS	5.59	/	/	2.83	1.92	/
17	Ellisiiamide F	NRPS	/	/	/	/	/	/
18	Ellisiiamide G	NRPS	6.46	/	/	/	/	/
19	Ellisiiamide H	NRPS	/	/	/	/	/	/

* Assignments based on the parent molecular ion [M+H]+, double charged species [2M+H]+, including Na and NH4 adducts, precursor ion fragments and HRS-MS/MS analaysis.

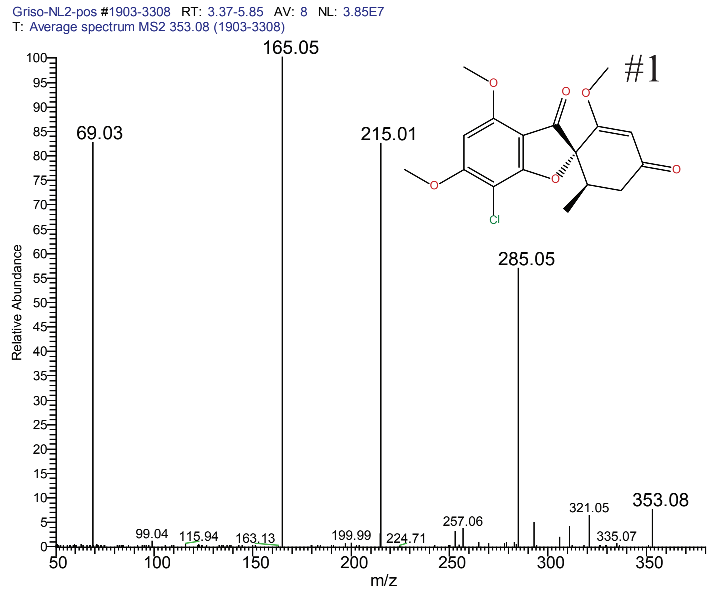
Griso-NL2-pos #1903-3308 RT: 3.37-5.85 AV: 8 NL: 3.85E7
T: Average spectrum MS2 353.08 (1903-3308)
#1
165.05
69.03
215.01
285.05
353.08
321.05
257.06
99.04
115.94
163.13
199.99
224.71
335.07
Cl
Relative Abundance
m/z

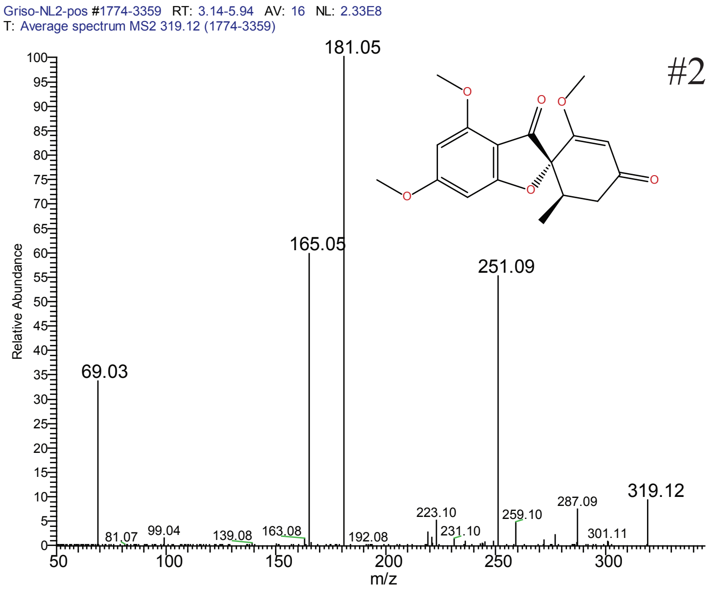
Griso-NL2-pos #1774-3359 RT: 3.14-5.94 AV: 16 NL: 2.33E8
T: Average spectrum MS2 319.12 (1774-3359)
#2
181.05
165.05
251.09
69.03
319.12
287.09
223.10
259.10
81.07
99.04
139.08
163.08
192.08
231.10
301.11
Relative Abundance
m/z

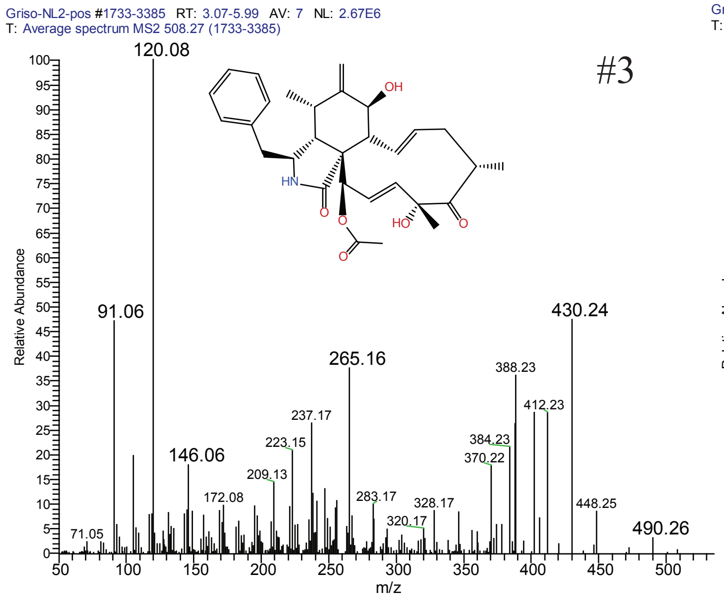
Griso-NL2-pos #1733-3385 RT: 3.07-5.99 AV: 7 NL: 2.67E6
T: Average spectrum MS2 508.27 (1733-3385)
#3
120.08
91.06
430.24
265.16
388.23
412.23
237.17
223.15
384.23
370.22
146.06
209.13
172.08
283.17
328.17
448.25
320.17
71.05
490.26
OH
HN
HO
Relative Abundance
m/z

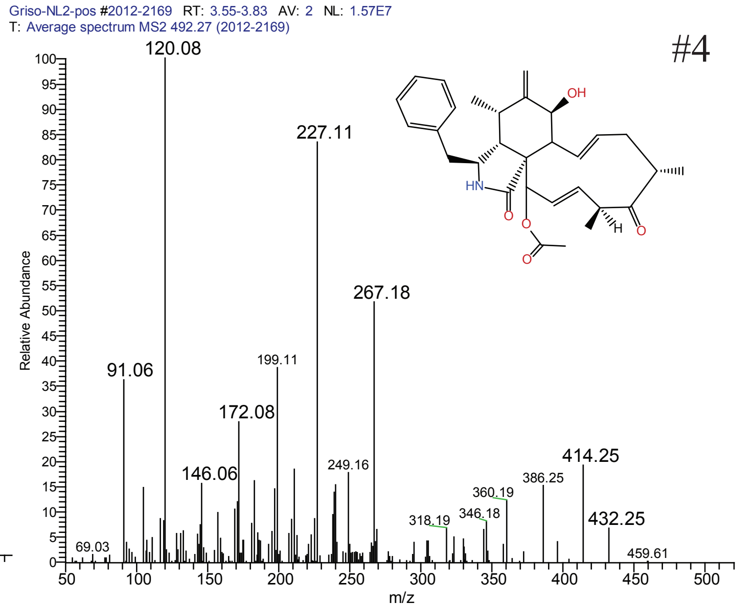
Griso-NL2-pos #2012-2169 RT: 3.55-3.83 AV: 2 NL: 1.57E7
T: Average spectrum MS2 492.27 (2012-2169)
#4
120.08
227.11
267.18
91.06
199.11
172.08
146.06
249.16
414.25
386.25
360.19
346.18
318.19
432.25
69.03
459.61
OH
HN
H
Relative Abundance
m/z

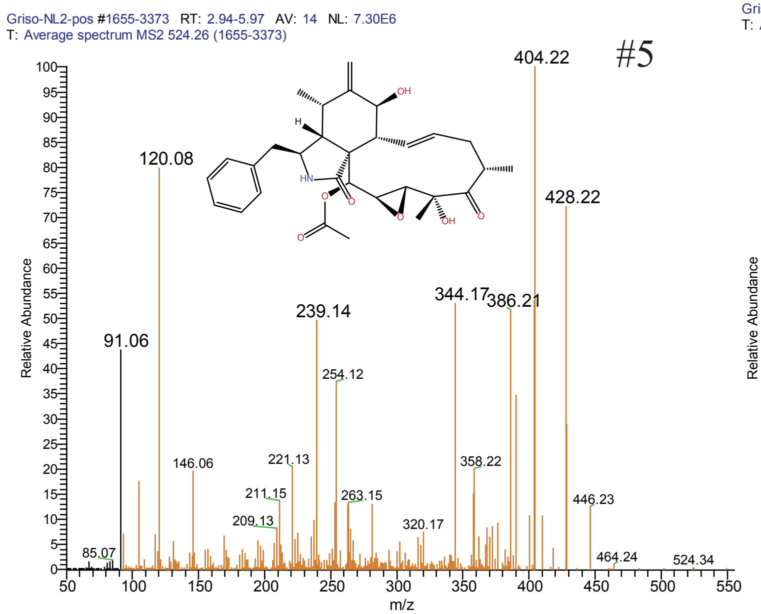
Griso-NL2-pos #1655-3373 RT: 2.94-5.97 AV: 14 NL: 7.30E6
T: Average spectrum MS2 524.26 (1655-3373)
#5
404.22
120.08
428.22
344.17
386.21
239.14
91.06
254.12
146.06
221.13
358.22
211.15
263.15
446.23
209.13
320.17
85.07
464.24
524.34
OH
H
HN
Relative Abundance
m/z

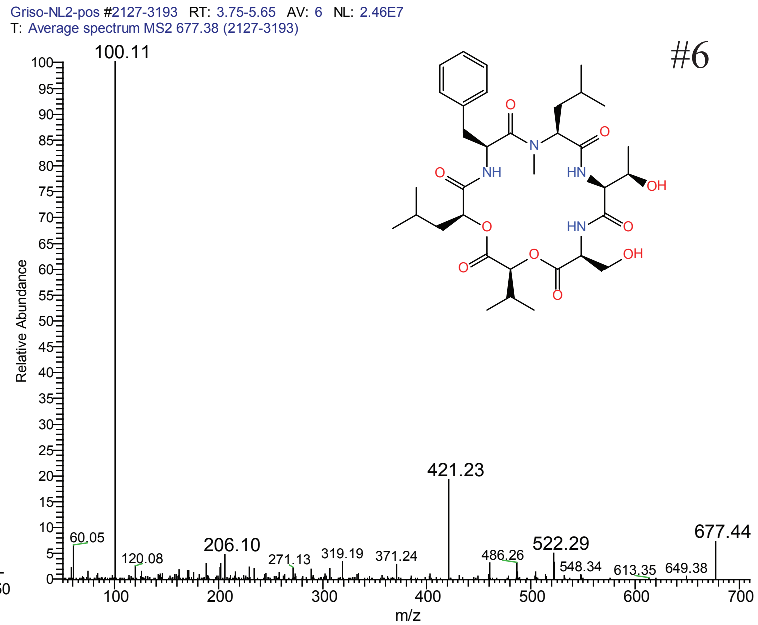
Griso-NL2-pos #2127-3193 RT: 3.75-5.65 AV: 6 NL: 2.46E7
T: Average spectrum MS2 677.38 (2127-3193)
#6
100.11
421.23
206.10
677.44
522.29
60.05
120.08
271.13
319.19
371.24
486.26
548.34
613.35
649.38
NH
HN
OH
Relative Abundance
m/z

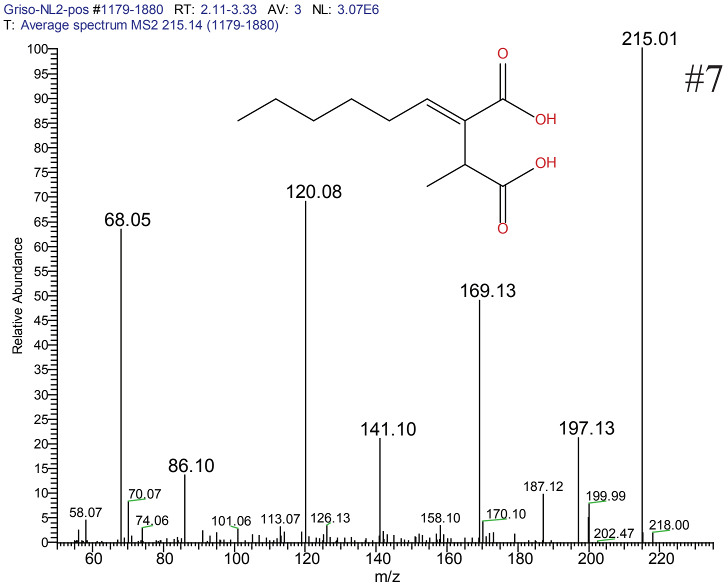

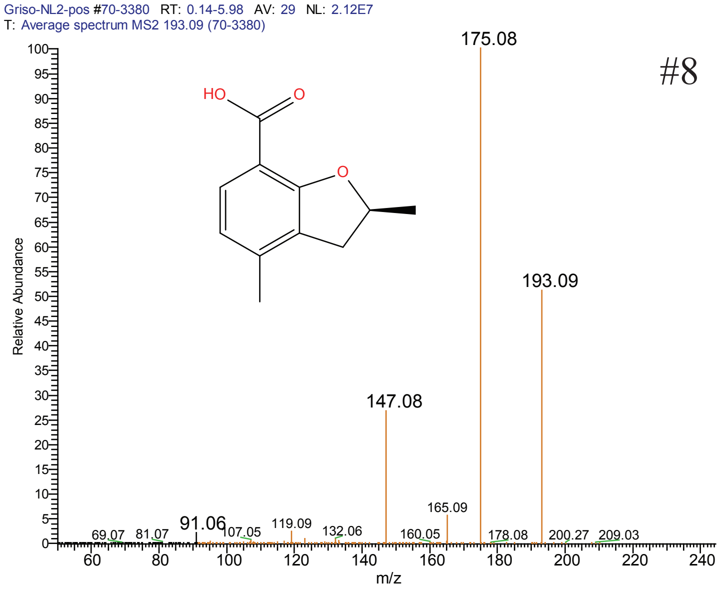

Figure 3S LC-MS/MS spectra of known compounds **1**-**8**.

Table 9S A. Cyclic nonribosomal peptide HRMS and peptide monomer sequences. Total of nine peptides have been identified, of these, eight are new secondary metabolites. Putative identifications for ellisiiamide D-H.

Metabolite	Rt (min)	Formula	Measured * [M+H]+	Calculated [M+H]+	ppm error	Peptide Sequence (MS/MS) 1	2	3	4	5
**Cyclic Pentapeptide 1	16.20	$C_{32}H_{50}N_5O_5$	584.3816	584.3806	-1.71	N-Me-Phe	Val	Ile	Leu	Pro
Cyclic Pentapeptide 2	14.28	$C_{28}H_{50}N_5O_5$	536.3819	536.3806	-2.42	Leu	Val	Ile	Leu	Pro
Xylarotide A	15.97	$C_{29}H_{52}N_5O_5$	550.3973	550.3963	-1.82	N-Me-Leu	Val	Ile	Leu	Pro
**Ellisiiamide A	14.76	$C_{30}H_{46}N_5O_5$	556.3501	556.3493	-1.44	N-Me-Phe	Ala	Ile	Leu	Pro
**Ellisiiamide B	15.19	$C_{31}H_{48}N_5O_5$	570.3656	570.3650	-1.05	N-Me-Phe	Val	Val	Leu	Pro
**Ellisiiamide C	17.04	$C_{33}H_{52}N_5O_5$	598.3968	598.3963	-0.84	N-Me-Phe	Ile	Ile	Leu	Pro
Ellisiiamide D	14.47	$C_{27}H_{48}N_5O_5$	522.3662	522.3650	-2.30	Leu	Val	Val	Leu	Pro
Ellisiiamide E	16.89	$C_{30}H_{54}N_5O_5$	564.4132	564.4119	-2.30	N-Me-Leu	Ile	Ile	Leu	Pro
Ellisiiamide F	14.11	$C_{31}H_{48}N_5O_6$	586.3616	586.3599	-1.72	N-Me-Phe	Val	Val	Leu	Pro+16
Ellisiiamide G	14.00	$C_{32}H_{50}N_5O_6$	600.3768	600.3756	-2.00	N-Me-Phe	Val	Ile	Leu	Pro+16
Ellisiiamide H	14.89	$C_{33}H_{52}N_5O_6$	614.3936	614.3912	-2.41	N-Me-Phe	Ile	Ile	Leu	Pro+16

* HRMS and MSMS measurements performed on modified HPLC gradient for improved metabolite resolution.
** Structural characterization by comprehensive 1D and 2D NMR and MS/MS analysis

Table 9S B. Optical rotation measurments for the isolated cyclic pentapeptides 1, 12-15.

Compound	Wt. meas. (mg)	Wt. g/100ml	Solvent	Vol. (mL)	Spec. Rot. o	StDev	Temp oC
Cyclic Pentapeptide 1	4.79	0.177	MeOH	2.7	-63.356	0.1932	20.9
Ellisiiamide A	1.59	0.059	MeOH	2.7	-86.138	0	21.1
Ellisiiamide B	1.03	0.038	MeOH	2.7	-43.101	0.8832	20.2
Ellisiiamide C	1.5	0.056	MeOH	2.7	-47.802	0	21.5

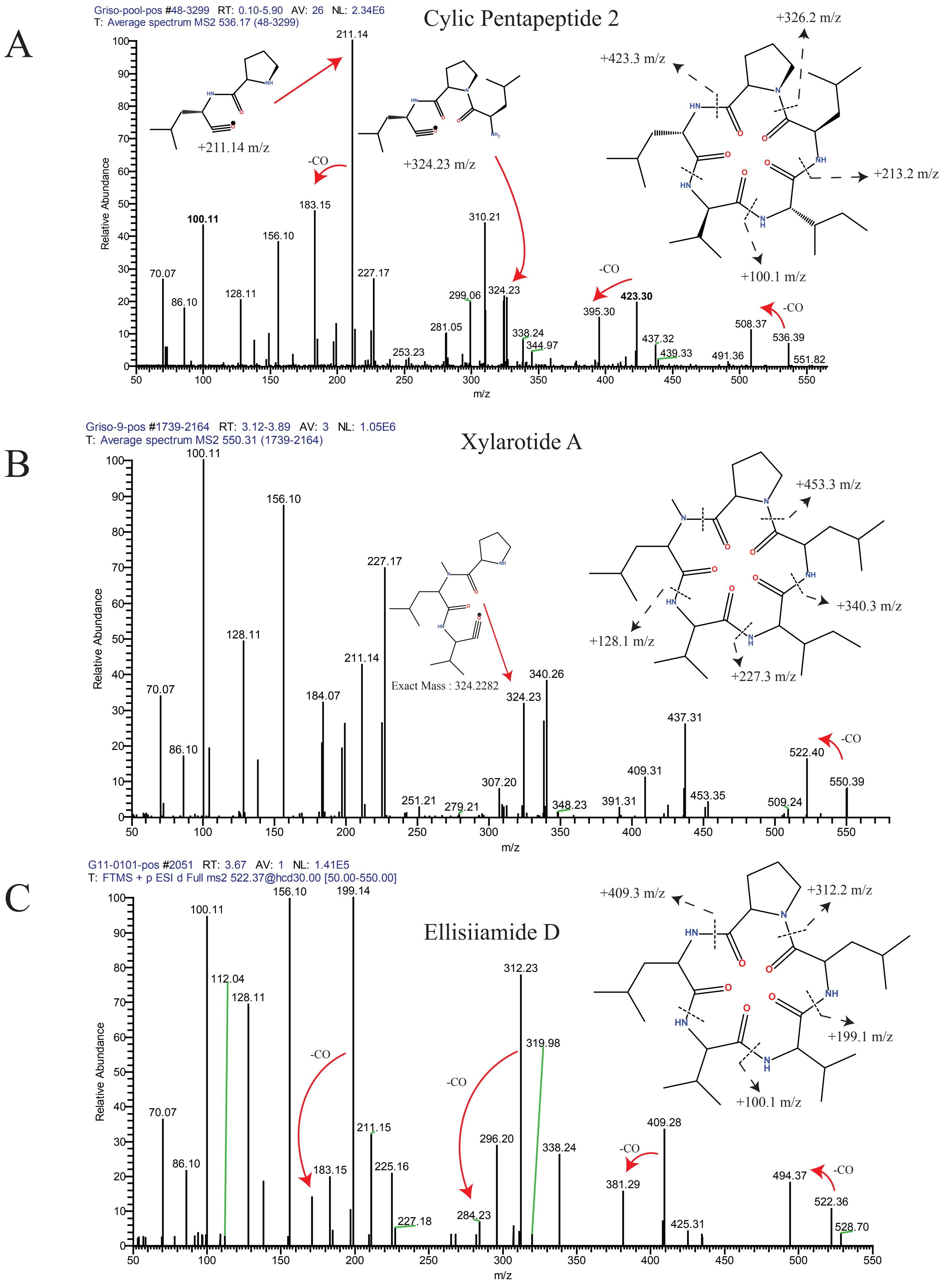

A
Griso-pool-pos #48-3299 RT: 0.10-5.90 AV: 26 NL: 2.34E6
T: Average spectrum MS2 536.17 (48-3299)
Cylic Pentapeptide 2
+211.14 m/z
+324.23 m/z
-CO
+326.2 m/z
+423.3 m/z
+213.2 m/z
+100.1 m/z
211.14
183.15
100.11
156.10
310.21
227.17
70.07
128.11
86.10
299.06
324.23
423.30
395.30
281.05
338.24
344.97
253.23
437.32
439.33
508.37
536.39
491.36
551.82
Relative Abundance
m/z
B
Griso-9-pos #1739-2164 RT: 3.12-3.89 AV: 3 NL: 1.05E6
T: Average spectrum MS2 550.31 (1739-2164)
Xylarotide A
Exact Mass : 324.2282
+453.3 m/z
+340.3 m/z
+128.1 m/z
+227.3 m/z
-CO
100.11
156.10
227.17
128.11
211.14
340.26
184.07
70.07
324.23
437.31
86.10
522.40
409.31
307.20
550.39
251.21
279.21
348.23
391.31
453.35
509.24
Relative Abundance
m/z
C
G11-0101-pos #2051 RT: 3.67 AV: 1 NL: 1.41E5
T: FTMS + p ESI d Full ms2 522.37@hcd30.00 [50.00-550.00]
Ellisiiamide D
+409.3 m/z
+312.2 m/z
+199.1 m/z
+100.1 m/z
-CO
156.10
199.14
100.11
112.04
128.11
312.23
319.98
70.07
211.15
296.20
338.24
409.28
86.10
183.15
225.16
381.29
494.37
522.36
227.18
284.23
425.31
528.70
Relative Abundance
m/z

D

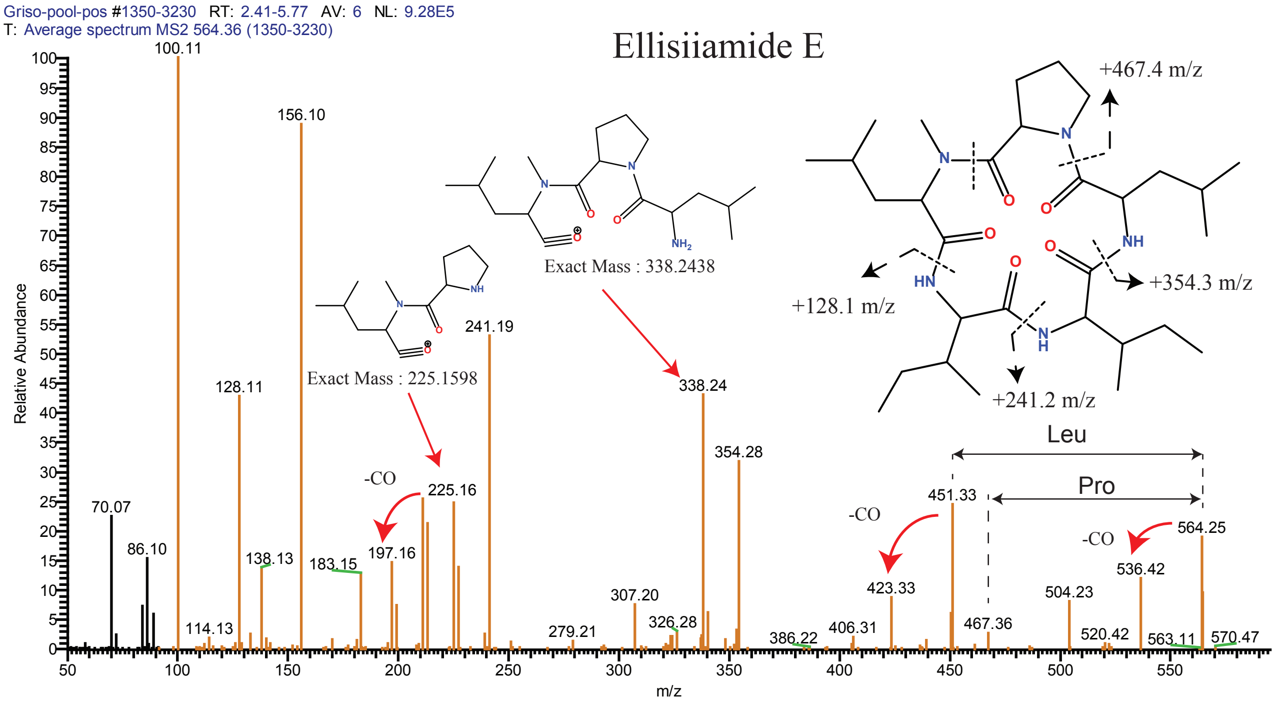

Griso-pool-pos #1350-3230 RT: 2.41-5.77 AV: 6 NL: 9.28E5
T: Average spectrum MS2 564.36 (1350-3230)
Ellisiiamide E
Relative Abundance
m/z
100.11
156.10
241.19
128.11
338.24
354.28
70.07
86.10
138.13
183.15
197.16
225.16
-CO
Exact Mass : 225.1598
Exact Mass : 338.2438
114.13
279.21
307.20
326.28
386.22
406.31
423.33
451.33
467.36
504.23
520.42
536.42
563.11
564.25
570.47
Leu
Pro
+467.4 m/z
+354.3 m/z
+128.1 m/z
+241.2 m/z

E

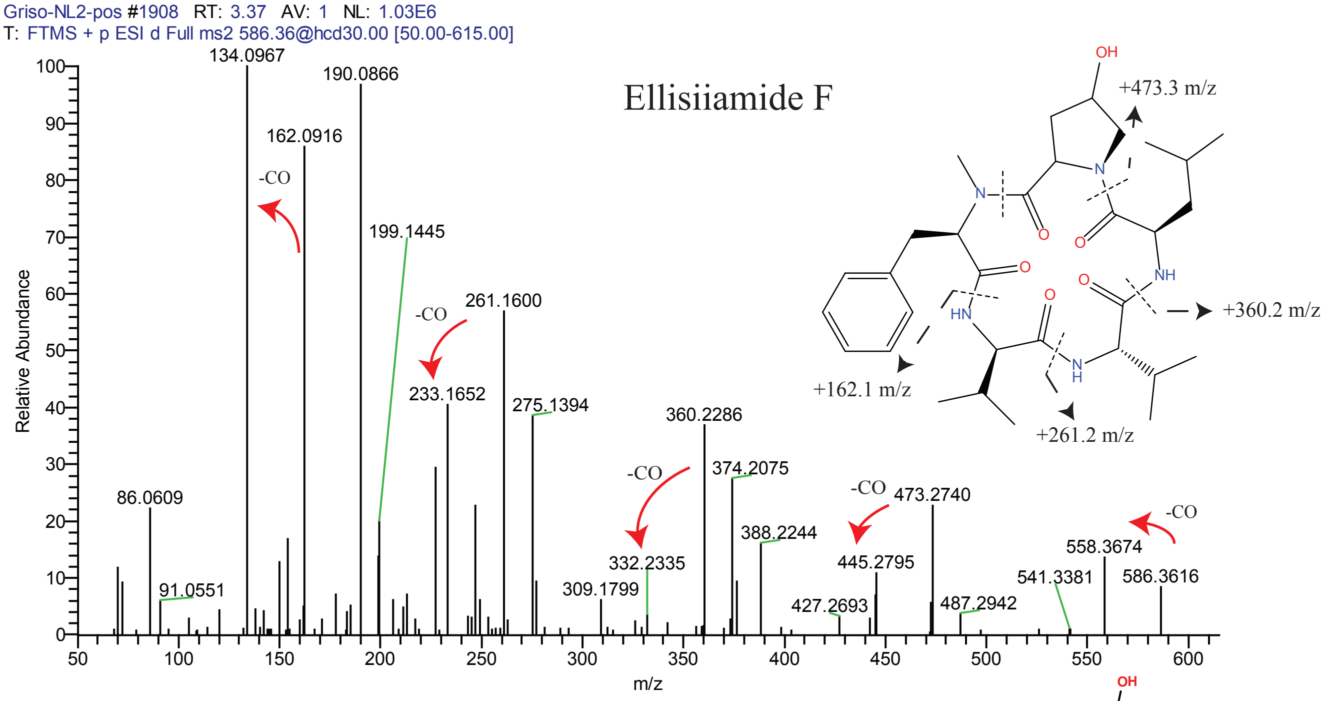

Griso-NL2-pos #1908 RT: 3.37 AV: 1 NL: 1.03E6
T: FTMS + p ESI d Full ms2 586.36@hcd30.00 [50.00-615.00]
Ellisiiamide F
Relative Abundance
m/z
134.0967
190.0866
162.0916
199.1445
261.1600
233.1652
275.1394
360.2286
374.2075
388.2244
86.0609
91.0551
309.1799
332.2335
427.2693
445.2795
473.2740
487.2942
541.3381
558.3674
586.3616
-CO
+473.3 m/z
+360.2 m/z
+162.1 m/z
+261.2 m/z

F

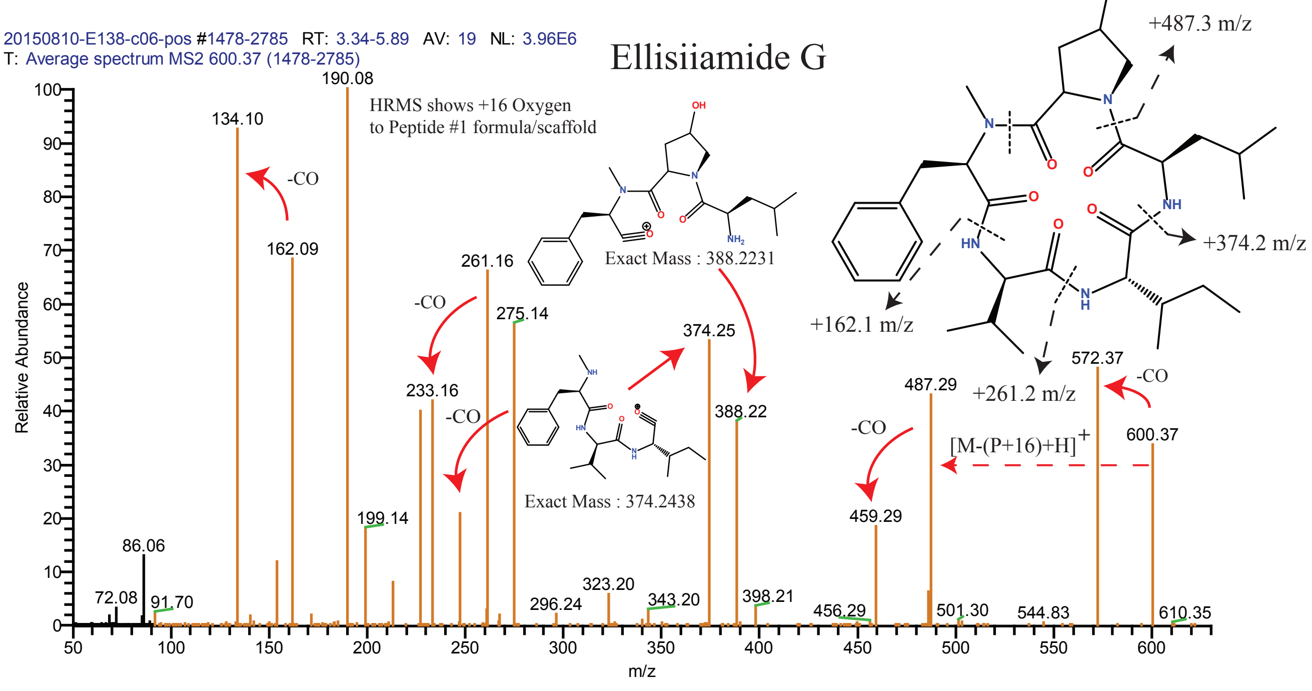

20150810-E138-c06-pos #1478-2785 RT: 3.34-5.89 AV: 19 NL: 3.96E6
T: Average spectrum MS2 600.37 (1478-2785)
Ellisiiamide G
HRMS shows +16 Oxygen
to Peptide #1 formula/scaffold
Relative Abundance
m/z
190.08
134.10
162.09
261.16
275.14
233.16
374.25
388.22
199.14
86.06
72.08
91.70
296.24
323.20
343.20
398.21
456.29
459.29
487.29
501.30
544.83
572.37
600.37
610.35
-CO
Exact Mass : 388.2231
Exact Mass : 374.2438
[M-(P+16)+H]+
+487.3 m/z
+374.2 m/z
+162.1 m/z
+261.2 m/z

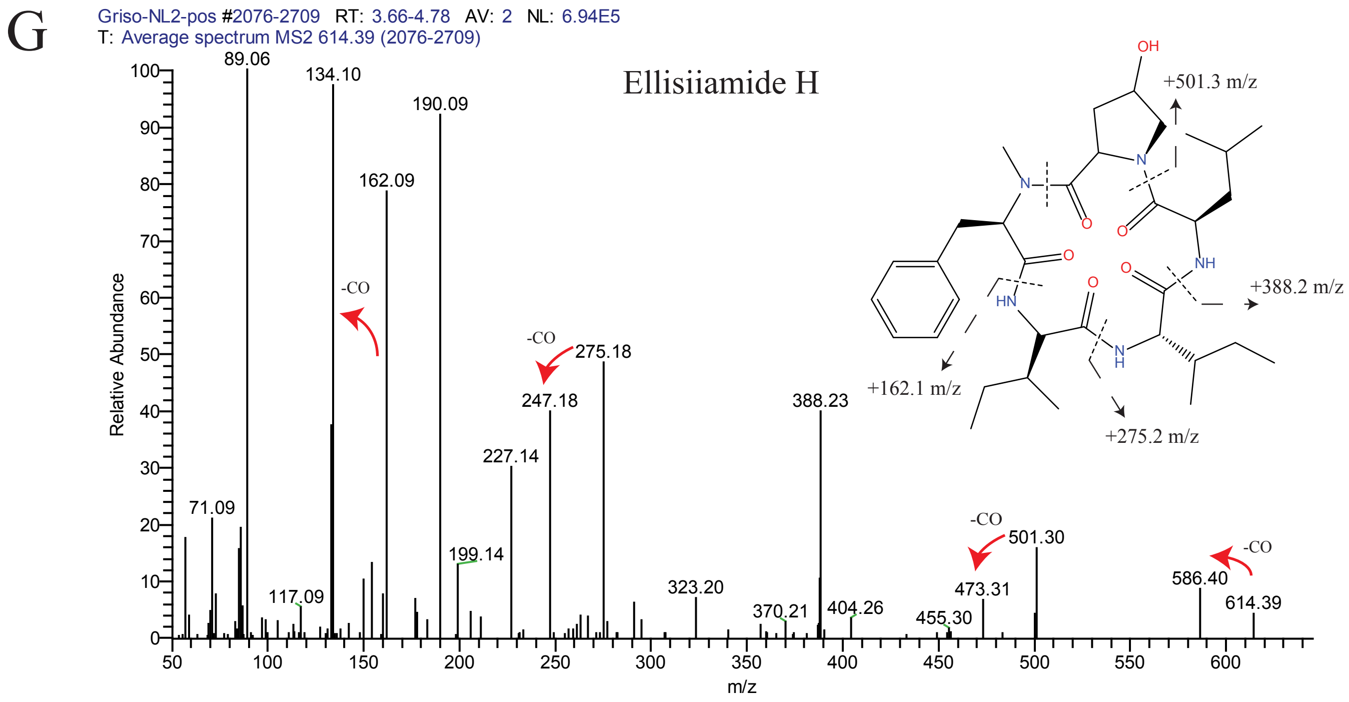

Figure 4AS. Q-Exactive MS/MS spectra of the cyclic pentapeptides with key diagnostic ions shown. (A) Cyclic pentapeptide 2, (B) Xylarotide A, (C) Cyclic pentapeptide 6, (D) Cyclic pentapeptide 7, (E) Cyclic pentapeptide 8, (F) Cyclic pentapeptide 9, (G) Cyclic pentapeptide 10.

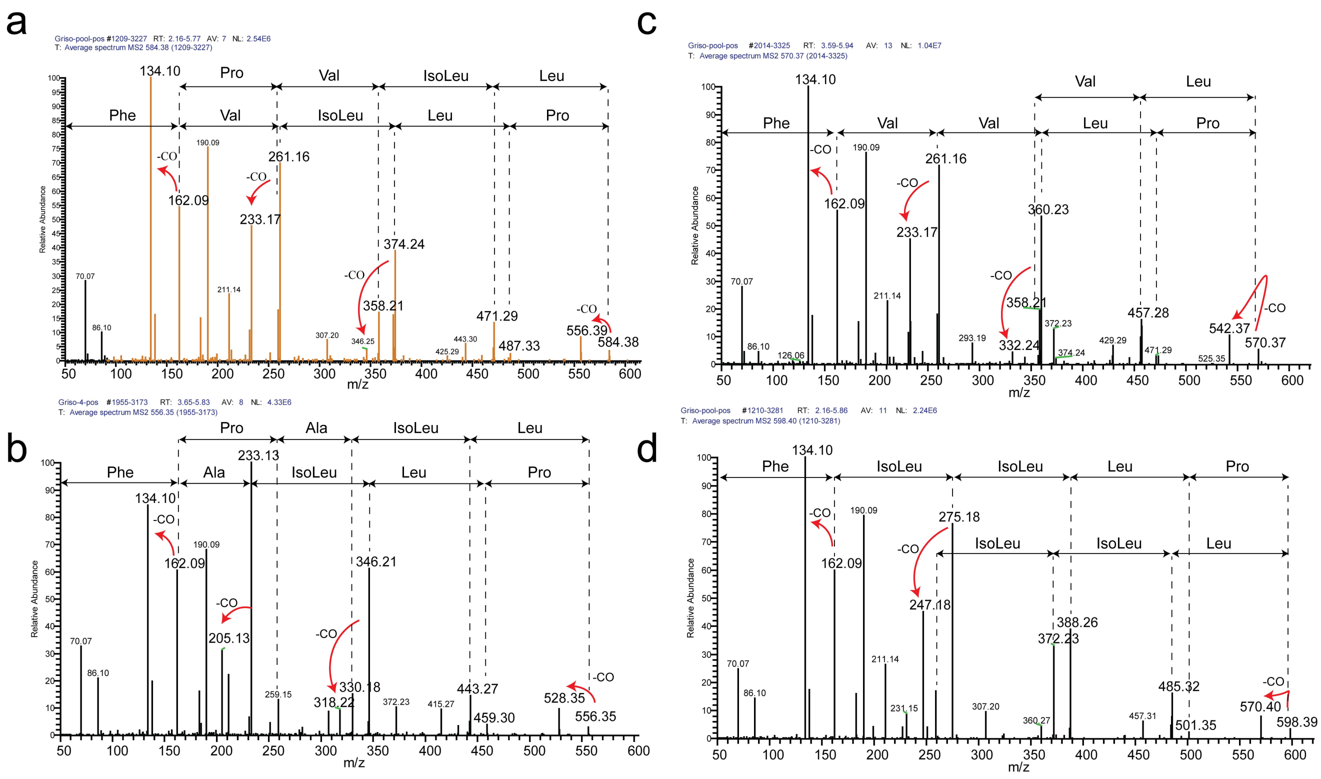

Figure 4BS. MS/MS spectra of cyclic pentapeptides; (A) cyclic pentapeptide #1 (**9**) , (B) ellisiiamide A (**12**), (C) ellisiiamide B (#**13**), (D) ellisiiamide C (**14**).

Table 10S. 1H (700 MHz) and 13C (176 MHz) NMR data and HMBC correlations for ellissiamide A ($DMSO_{d6}$)

Amio acid	position	δ_C	δ_H (*J* in Hz)	HMBC
L-*N*-methyl phenylalanine	CO	170.6		
	α	55.8	5.08 (dd, 11.9, 5.1)	*β, N*–CH3*, γ,* CO
	β	33.5	3.08 (m)	
			2.9 (dd, 14.8, 5.1)	
	γ	137.4		
	ortho	128.4	7.21 (m)	*meta, para, β*
	meta	128.1	7.27 (m)	*ortho, γ*
	para	126.4	7.20 (m)	*ortho*
	N-CH3	30.2	3.04 (s)	α, CO-L-Pro
L-alanine	CO	171.9		
	α	46.0	4.49 (dq, 8.9, 6.9)	*β,* CO, CO-N-MePhe
	β	14.2	1.1 (d, 7.0)	α, CO
	NH		8.52 (d, overlap)	α, CO-N-MePhe
D-isoleucine	CO	170.1		
	α	55.7	4.17 (dd, 8.3, 5.2)	*β, β*–CH3*, γ,* CO, CO-L-Ala
	β	38.5	1.50 (ddt, 14.1, 7.2, 3.5)	*α, β*–CH3*, δ,* CO
	γ	25.8	1.31 (m)	*α, β, β*–CH3*, δ*
			0.99 (dt, 13.4, 7.4)	
	β - CH3	14.4	0.72 (d, 6.8)	*α, β, γ,*
	δ	11.6	0.85 (t, 7.4)	*β, γ,*
	NH		6.94 (d,8.3)	α, CO, CO-L-Ala
L-leucine	CO	168.8		
	α	46.5	4.74 (m)	*β, γ,* CO, CO-L-Pro
	β	41.1	1.45 (m)	*α, γ, δ,* CO
			1.40 (m)	
	γ	24.2	1.39 (m)	*α, β, δ*
	δ	22.2	0.79 (d, 5.9)	*β, γ, δ*
		22.9	0.82 (d, 6.1)	
	NH		8.49 (d, overlap)	α, CO-D-Ile
L-proline	CO	172.2		
	α	58.9	5.10	*β, γ, δ,* CO, N-MePhe
	β	30.0	1.83 (tt, 12.1, 8.0)	*α, γ,* CO
			0.90 (dd, 12.1, 7.4)	
	γ	20.32	1.6 (m)	*α, β,*
			1.26 (m)	
	δ	45.7	3.27 (m)	*α, β, γ,* CO-Leu

Table 11S. 1H (850 MHz) and 13C (176 MHz) NMR data and HMBC correlations ellissiamide B ($DMSO_{d6}$)

Amio acid	position	δ_C	$\delta_{H\ (J\ in\ Hz)}$	HMBC
L-*N*-methyl phenylalanine	CO	170.5		
	α	56.0	5.10 (dd, 10.9, 6.0)	*β, N*–CH3*, γ,* CO
	β	33.8	3.05 (m)	α, γ, *ortho*, CO
			2.90 (dd, 14.5, 6.0)	
	γ	137.3		
	ortho	128.6	7.21 (m)	*meta, para, β*
	meta	128.1	7.26 (m)	*ortho, γ*
	para	126.4	7.19 (td, 7.1, 1.4)	*ortho*
	N-CH3	30.2	3.03 (s)	α, CO-L-Pro
L-valine	CO	170.8		
	α	57.6	3.95 (m)	*β, γ,* CO
	β	26.4	1.97 (dt, 10.5, 6.7)	α, γ, CO
	γ	18.5	0.73 (d, 6.7)	*α, β,*
		19.7	0.84 (d, 6.5)	
	NH		8.18 (d, 9.6)	α, CO-N-MePhe
D-valine	CO	170.1		
	α	56.8	4.10 (dd, 8.7, 6.7)	*β, γ,* CO
	β	31.3	1.74 (h, 6.8)	α, γ, CO
	γ	17.9	0.74 (d, 6.8)	*α, β,*
		19.2	0.78 (m)	
	NH		6.98 (d, 8.8)	α, CO-L-Val
L-leucine	CO	168.7		
	α	46.6	4.72 (q, 4.7, 7.2)	*β, γ,* CO
	β	41.4	1.46 (m)	*α, γ, δ,* CO
			1.38 (m)	
	γ	24.2	1.39 (m)	*α, β, δ,* CO
	δ	22.8	0.82 (d, 6.4)	*β, γ, δ*
		22.4	0.79 (m)	
	NH		8.43 (d, 9.4)	α, CO-D-Val
L-proline	CO	171.9		
	α	58.7	5.08 (d, 7.9)	*β, γ, δ,* CO, N-MePhe
	β	30.1	1.86 (tt, 12.1, 8.1)	*α, γ, δ,* CO, N-MePhe
			0.98 (dd, 12.1, 7.4)	
	γ	20.4	1.62 (dq, 13.1, 7.0, 6.6)	*α, β,*
			1.31 (m)	
	δ	45.71	3.30 (dd, 9.1, 5.9)	*α, β, γ,* CO-Leu

Table 12S. 1H (700 MHz) and 13C (176 MHz) NMR data and HMBC correlations for ellissiamide C ($DMSO_{d6}$)

Amio acid	position	δ_C	$\delta_{H\,(J\text{ in Hz})}$	HMBC
L-*N*-methyl phenylalanine	CO	170.3		
	α	56.0	5.1 (m)	*β, N*–CH3*, γ,* CO
	β	33.8	3.03 (m)	α, γ, *ortho*, CO
			2.90 (dd, 14.4, 6.1)	
	γ	137.3		
	ortho	128.6	7.21 (m)	*meta, para, β*
	meta	128.1	7.26 (m)	*ortho, γ*
	para	126.3	7.19 (m)	*ortho*
	N-CH3	30.2	3.04 (s)	α, CO-L-Pro
L- isoleucine	CO	171.0		
	α	55.8	4.05 (dd, 10.8, 9.6)	*β, β*–CH3*, γ,* CO
	β	32.0	1.82 (m)	α
	γ	24.0	1.30 (m)	*β,β*–CH3*, δ*
			0.92 (m)	
	β - CH3	15.7	0.79 (m)	*α, β, γ,*
	δ	10.1	0.74 (t, 7.4)	*β, γ,*
	NH		8.12 (d, 9.6)	α, CO-N-MePhe
D-isoleucine	CO	170.3		
	α	55.1	4.25 (dd, 8.8, 5.4)	*β, β*–CH3*, γ,* CO
	β	37.7	1.55 (p, 6.5)	*α, β*–CH3*, δ,* CO
	γ	25.9	1.27 (m)	*α, β, β*–CH3*, δ*
			1.01 (m)	
	β - CH3	14.3	0.7 (6.8)	*α, β, γ,*
	δ	11.5	0.85 (t, 7.4)	*β, γ,*
	NH		6.94 (d, 8.8)	α, CO-L-Ile
L-leucine	CO	168.8		
	α	46.5	4.72 (td, 9.1, 8.5, 6.5)	*β, γ,* CO
	β	41.2	1.44 (m)	*α, γ, δ,* CO
			1.41 (m)	
	γ	24.2	1.4 (m)	*α, β, δ*
	δ	22.2	0.80 (m)	*β, γ, δ*
		22.9	0.83 (d, 6.1)	
	NH		8.45 (d, 9.4)	α, CO-D-Ile
L-proline	CO	171.9		
	α	58.7	5.09 (m)	*β, γ, δ,* CO, N-MePhe
	β	30.0	1.85 (m)	*α, γ, δ,* CO, N-MePhe
			0.98 (dd, 12.6, 7.6)	
	γ	20.4	1.62 (m)	*α, β,*
			1.29 (m)	
	δ	45.7	3.29 (td, 7.8, 7.4, 3.3)	*α, β, γ,* CO-Leu

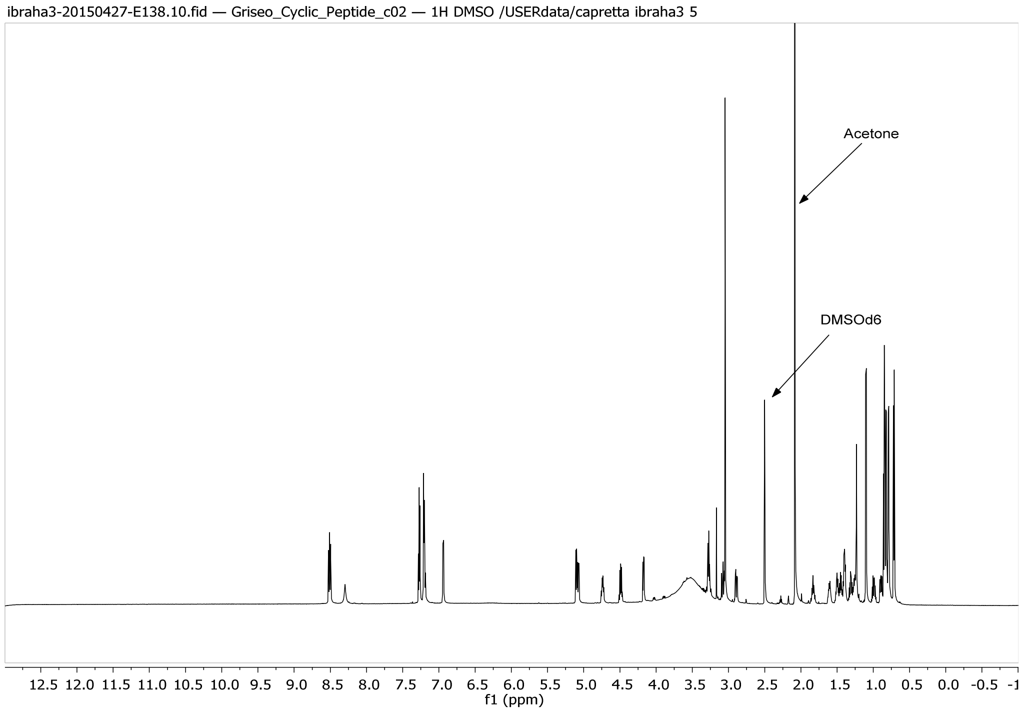

Figure 5S. ^{1}H spectrum of ellisiiamide A (700 MHz, $DMSO_{d6}$)

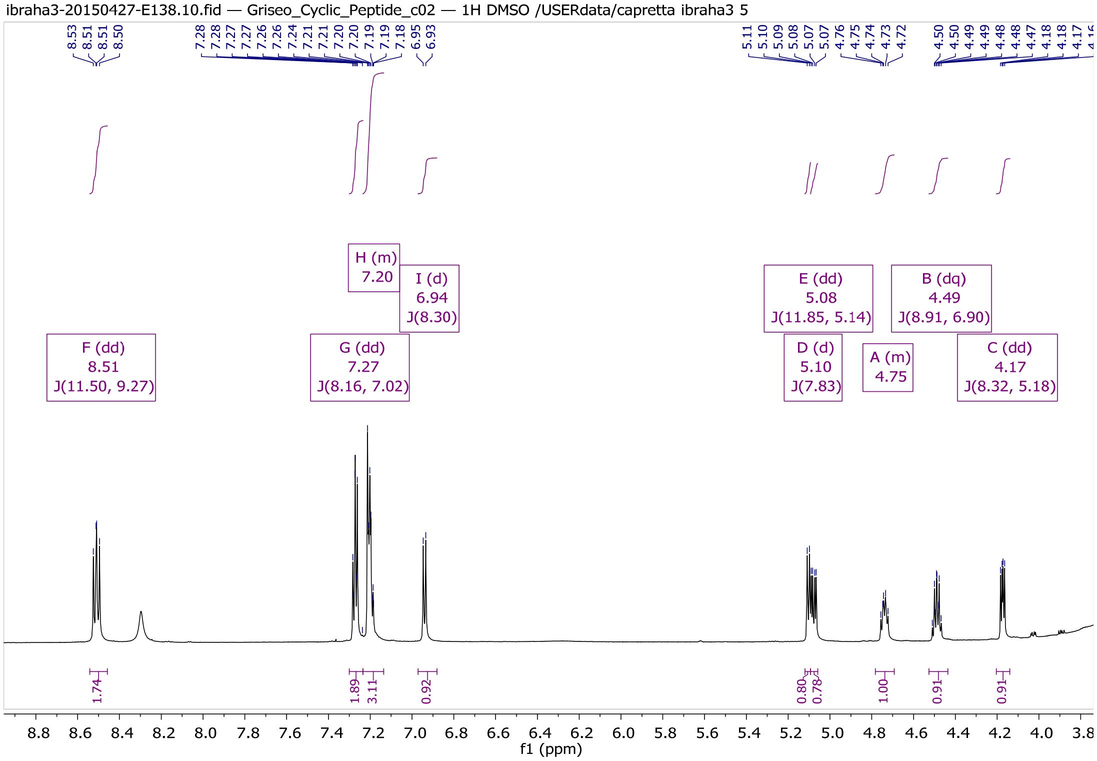

Figure 6S. Expanded ^{1}H spectrum of ellisiiamide A (700 MHz, $DMSO_{d6}$).

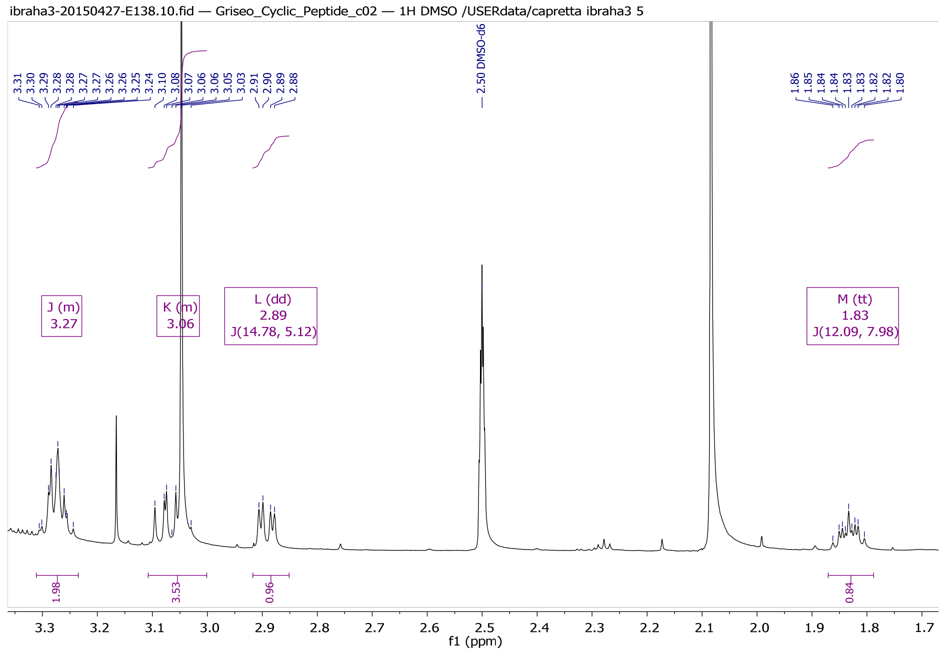

Figure 7S. Expanded ^{1}H spectrum of ellisiiamide A (700 MHz, DMSO $_{d6}$).

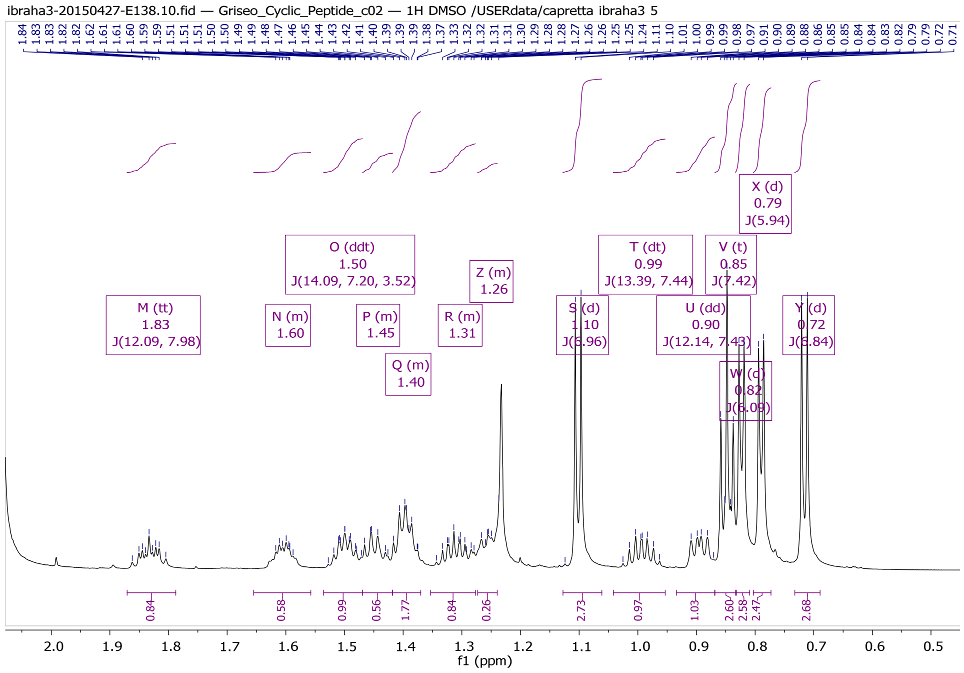

Figure 8S. Expanded ^{1}H spectrum of ellisiiamide A (700 MHz, DMSO $_{d6}$).

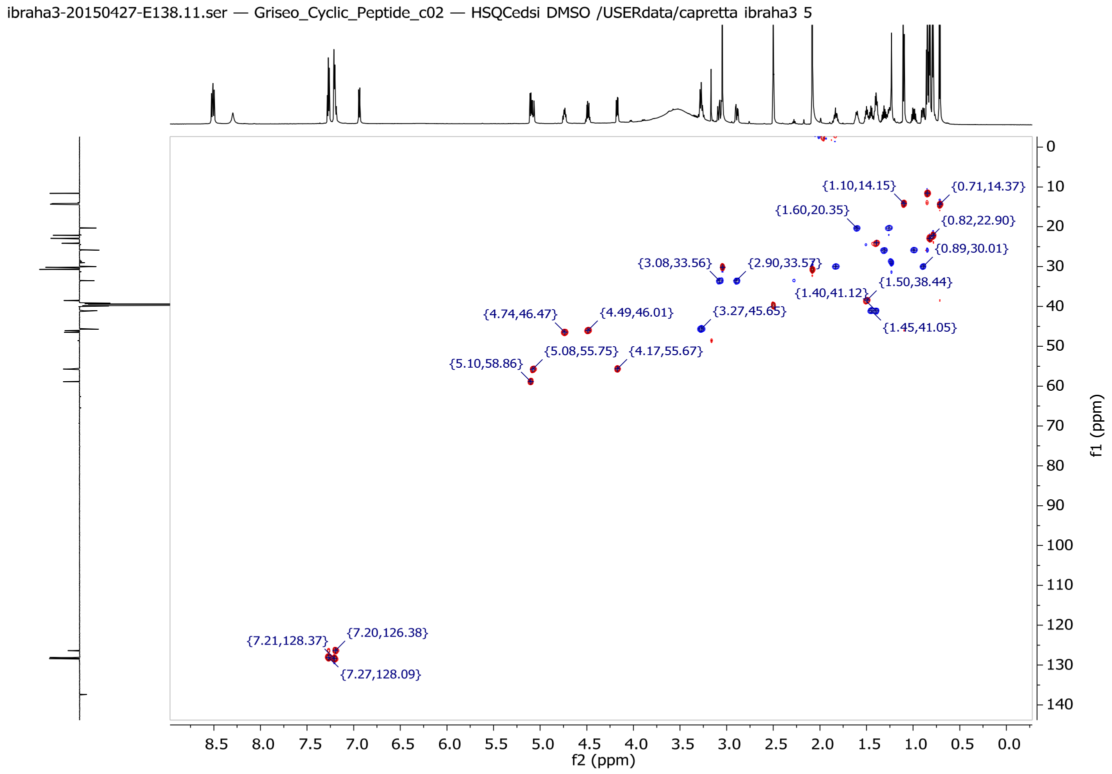

Figure 9S. HSQC spectrum of ellisiiamide A (700 MHz, DMSO $_{d6}$), multiplicity edited.

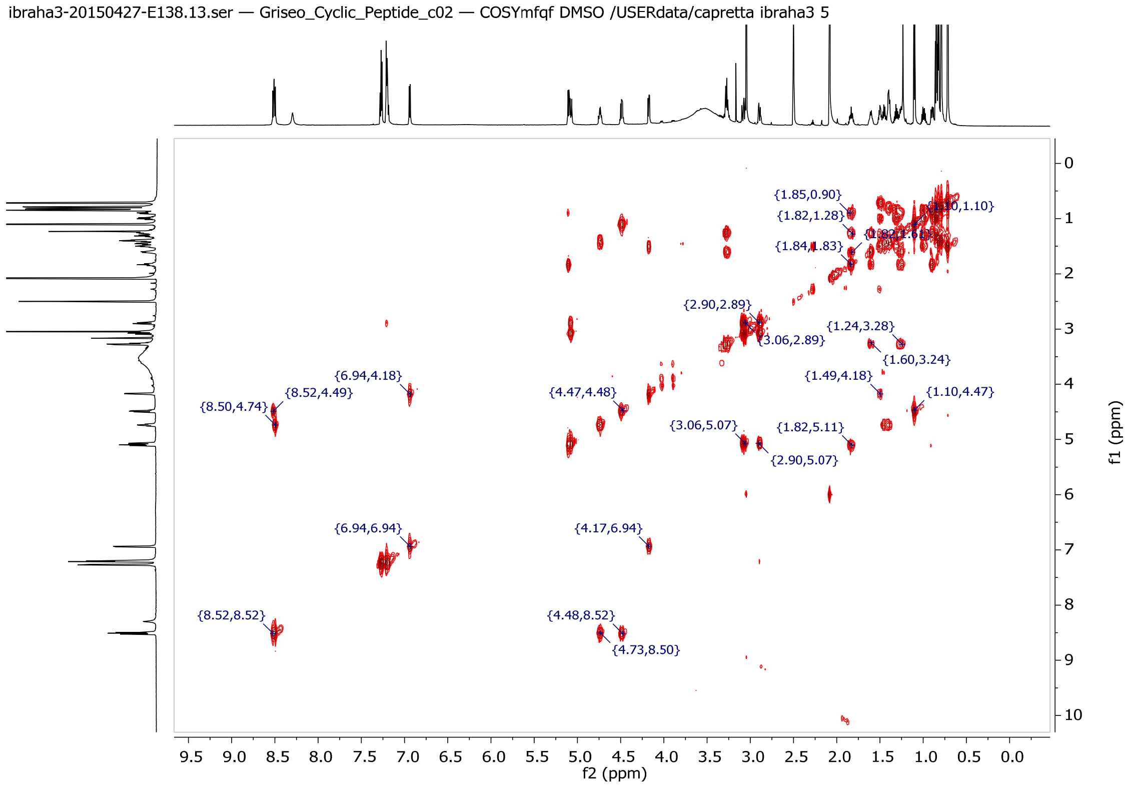

Figure 10S. COSY spectrum of ellisiiamide A (700 MHz, DMSO $_{d6}$).

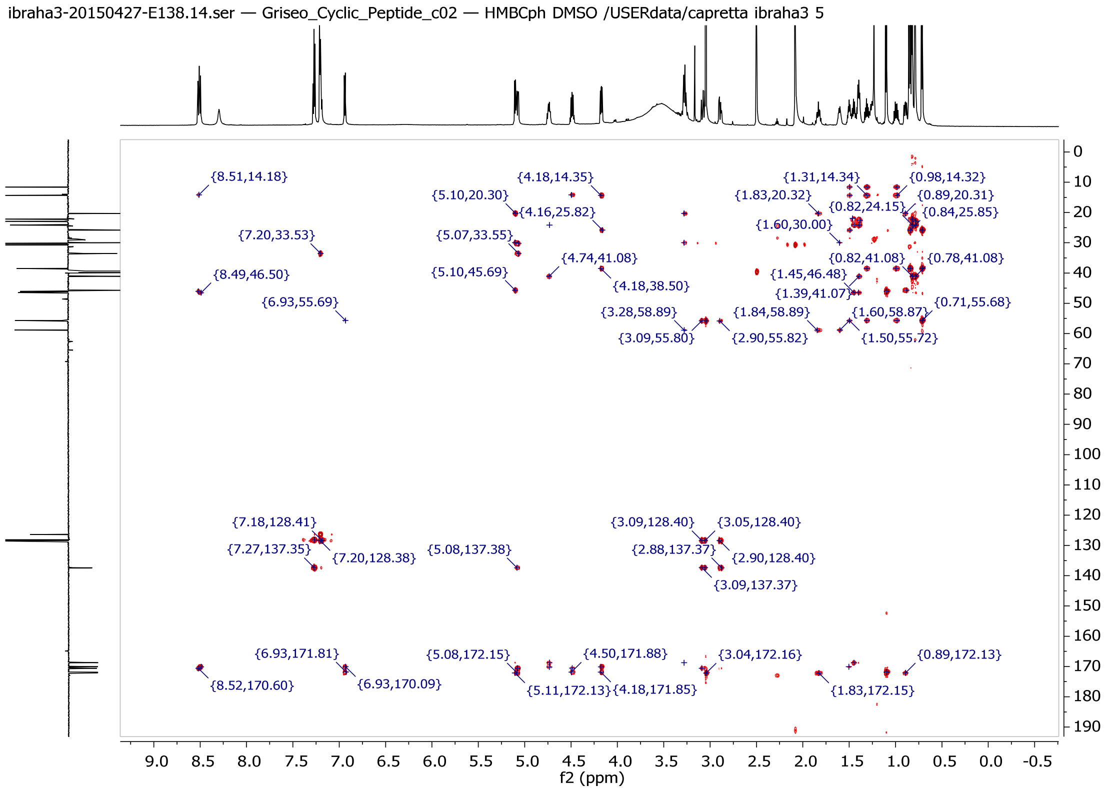

Figure 11S. HMBC spectrum of ellisiiamide A (700 MHz, DMSO $_{d6}$).

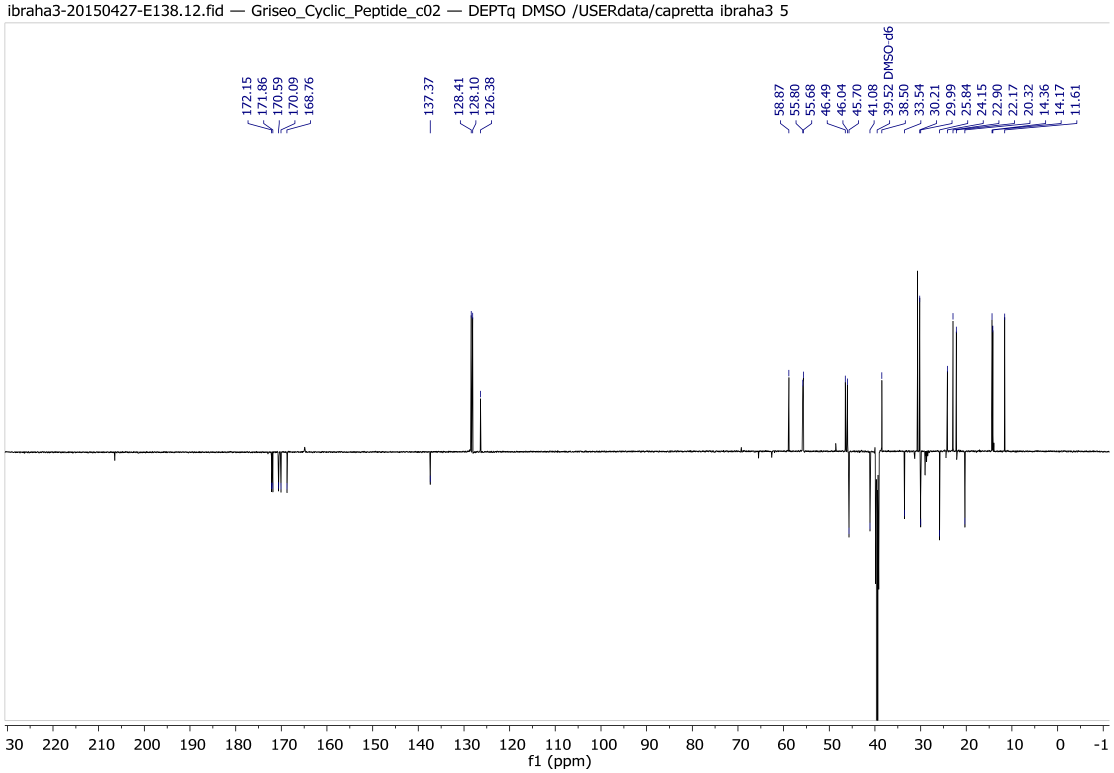

Figure 12S. ^{13}C -DEPTq spectrum of ellisiiamide A (176 MHz, DMSO $_{d6}$).

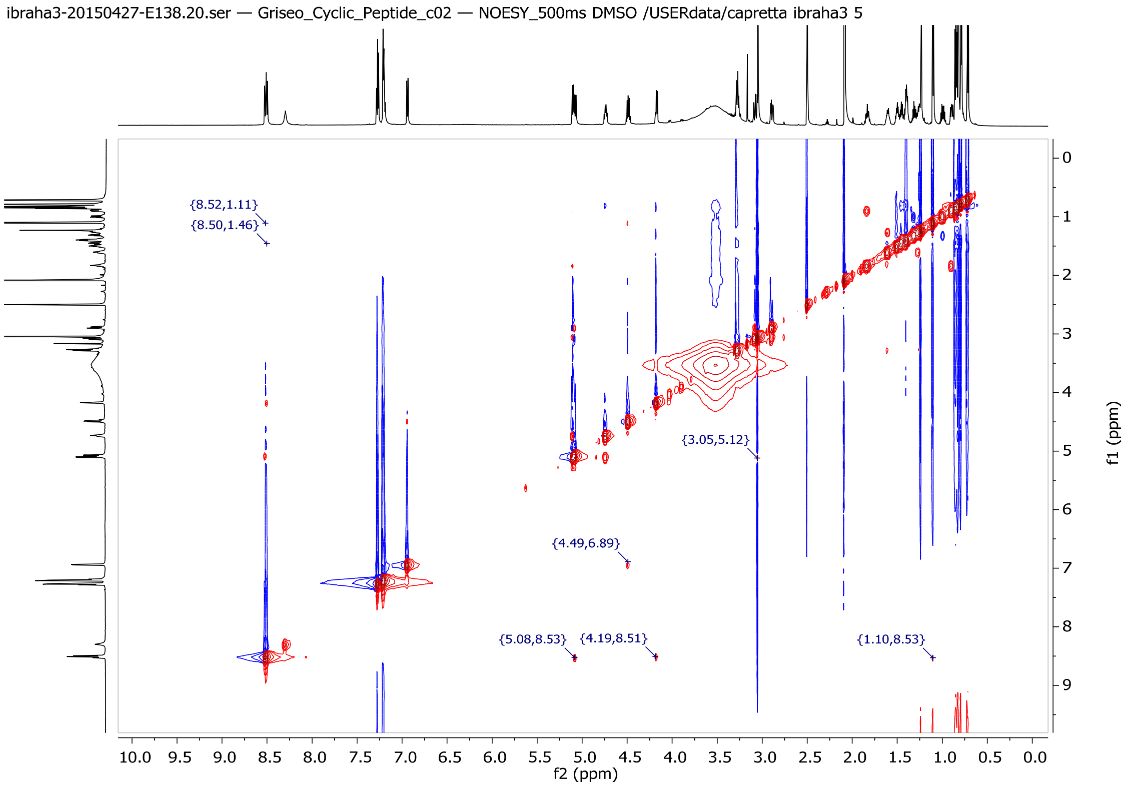

Figure 13S. NOESY spectrum of ellisiiamide A (700 MHz, DMSO $_{d6}$).

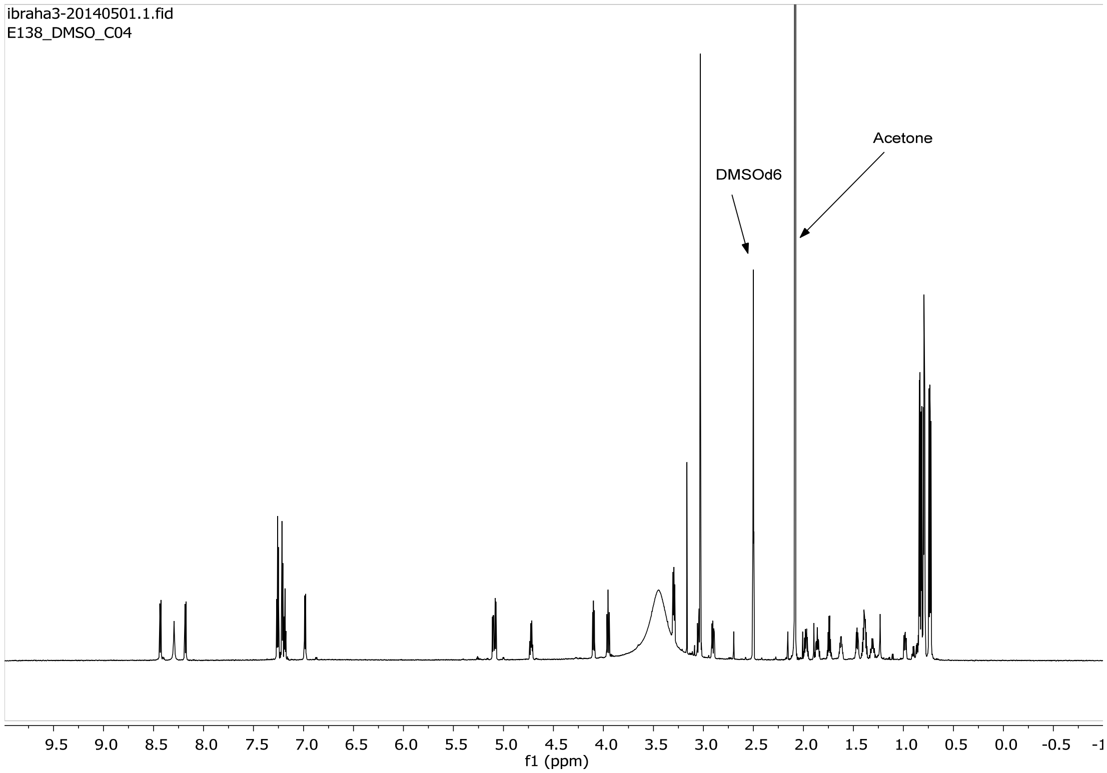

Figure 14S. ^{1}H spectrum of ellisiiamide B (850 MHz, DMSO $_{d6}$)

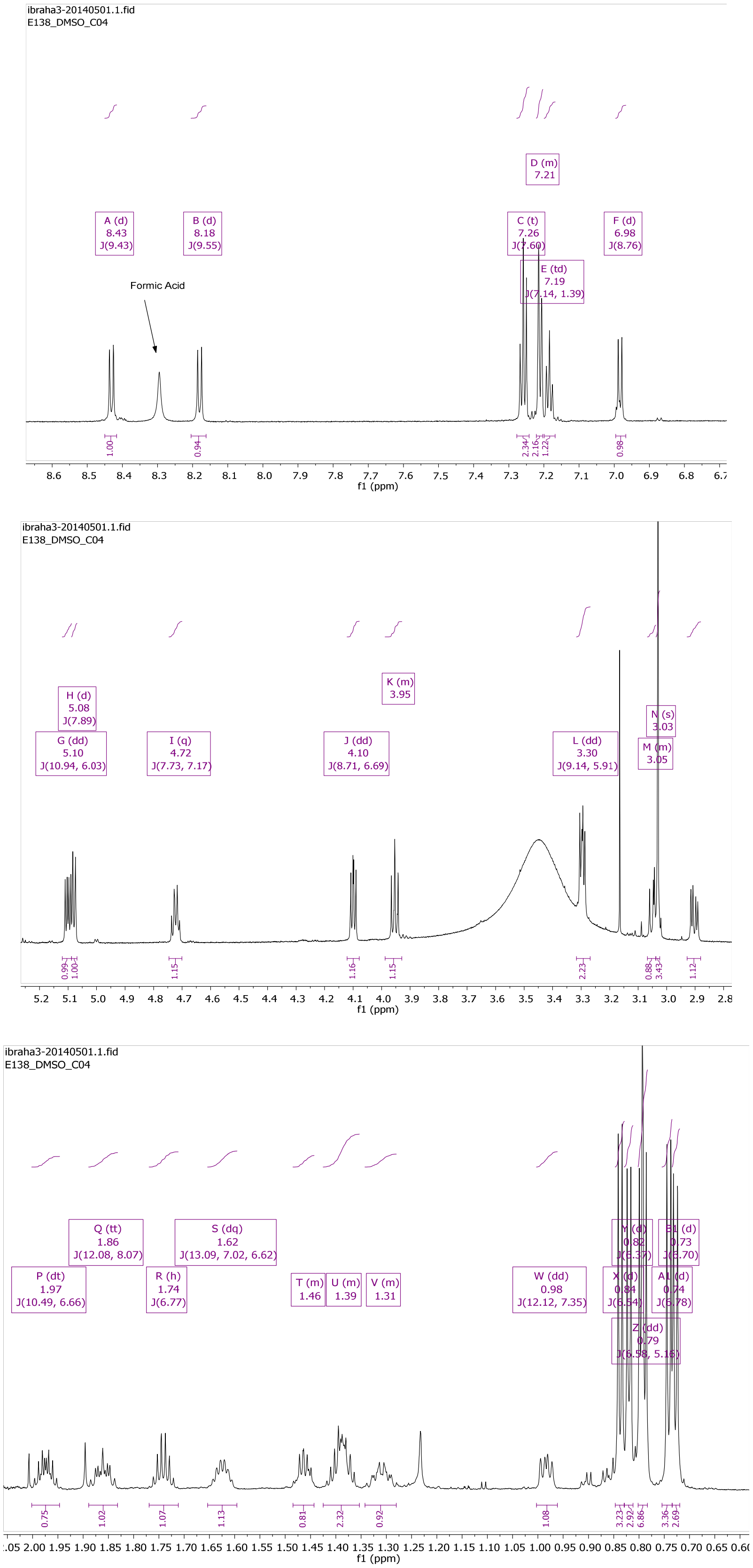

Figure 15S. Expanded ^{1}H spectrums of ellisiiamide B (850 MHz, DMSO $_{d6}$)

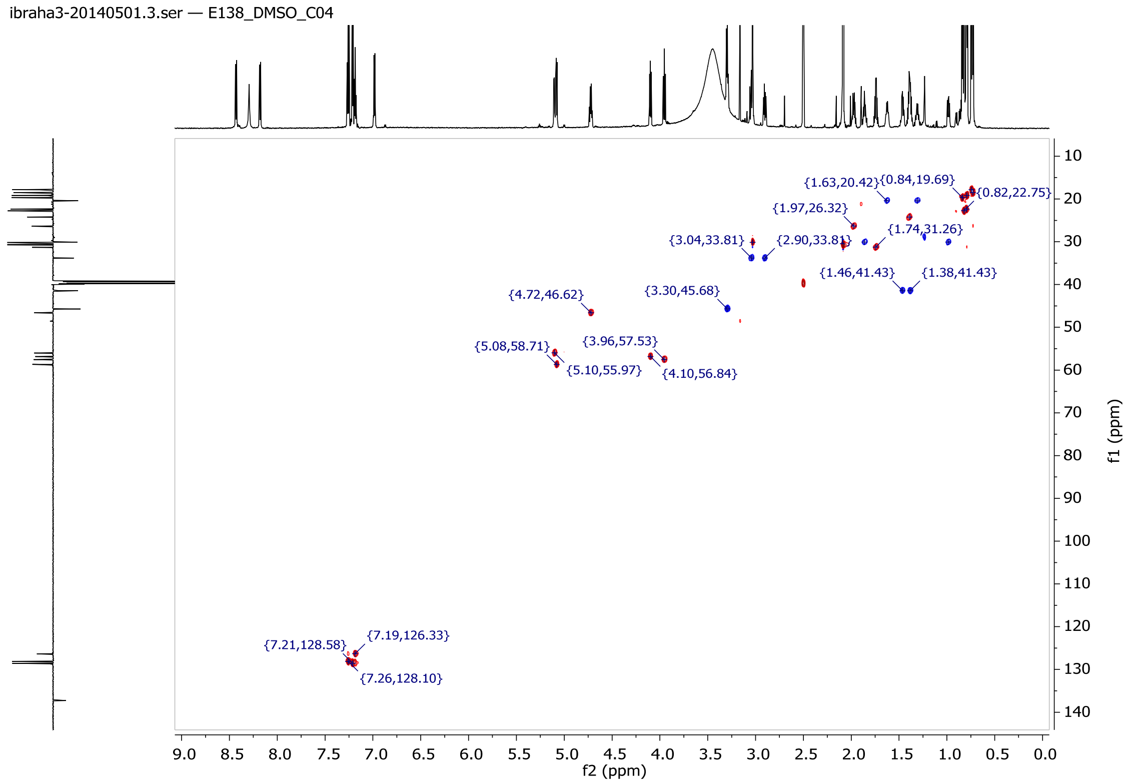

Figure 16S. HSQC spectrum of ellisiiamide B (850 MHz, DMSO $_{d6}$), multiplicity edited.

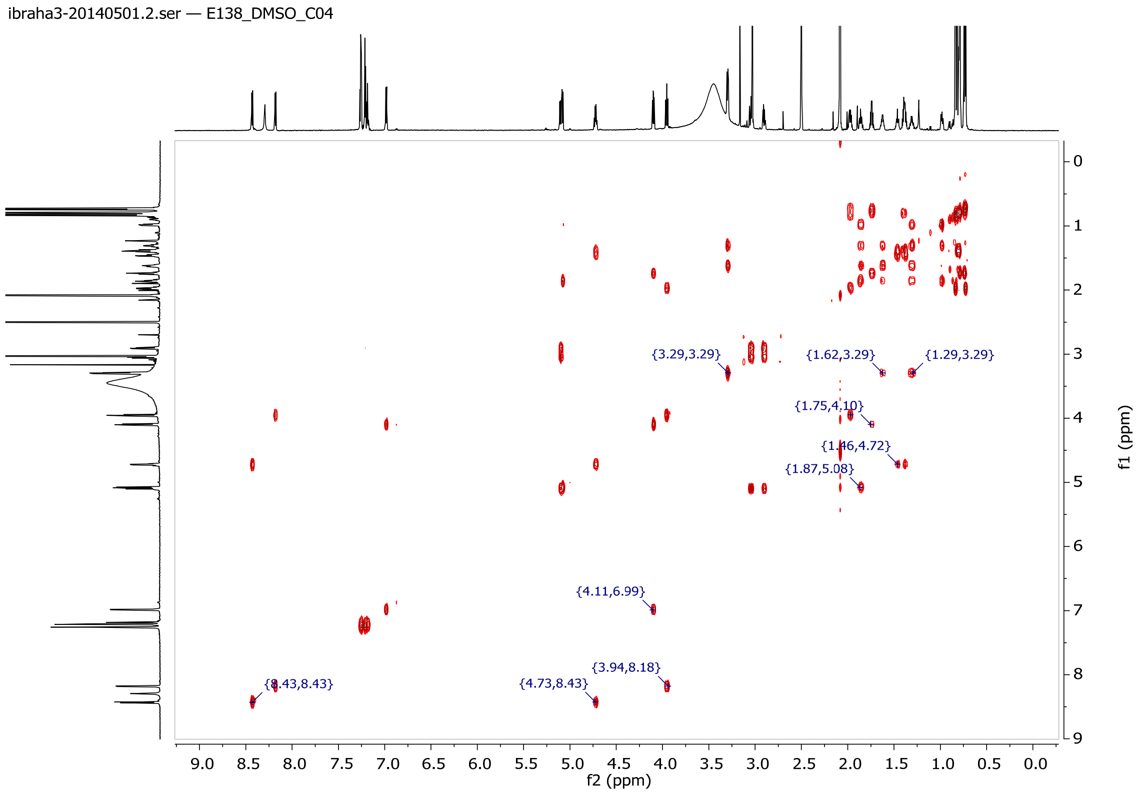

Figure 17S. COSY spectrum of ellisiiamide B (850 MHz, DMSO $_{d6}$).

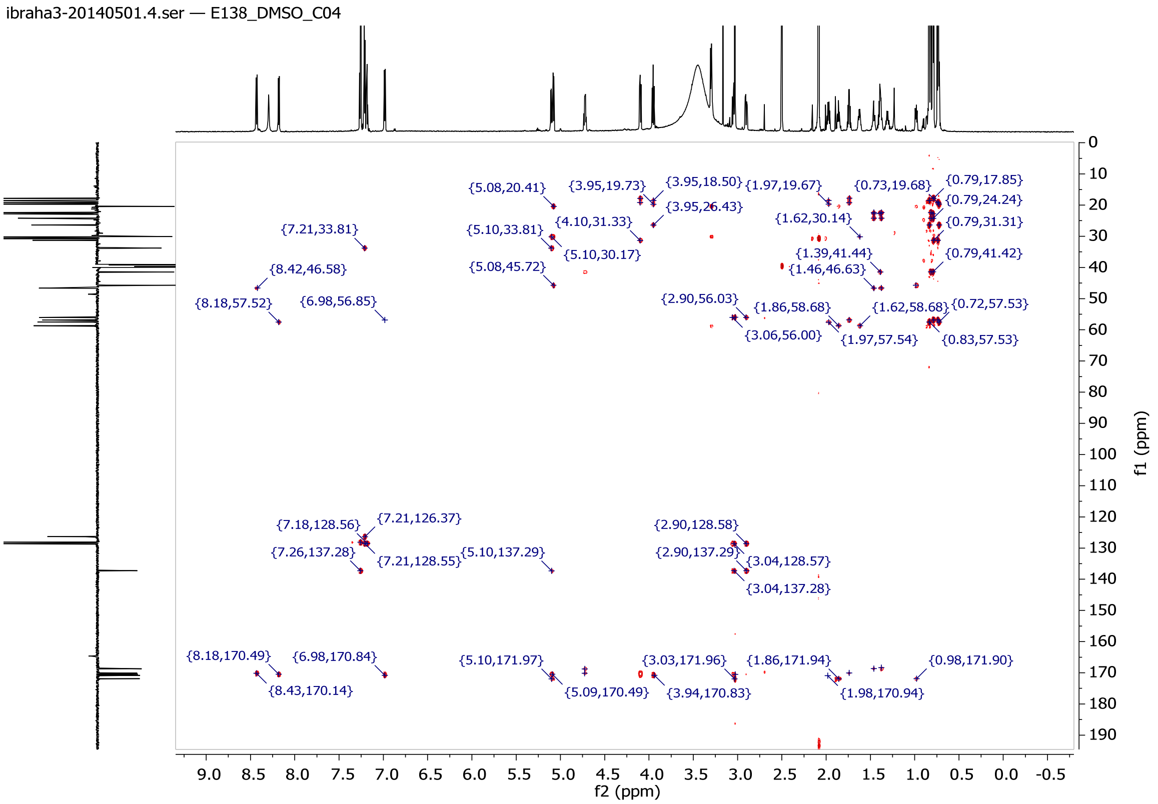

Figure 18S. HMBC spectrum of ellisiiamide B (850 MHz, DMSO $_{d6}$).

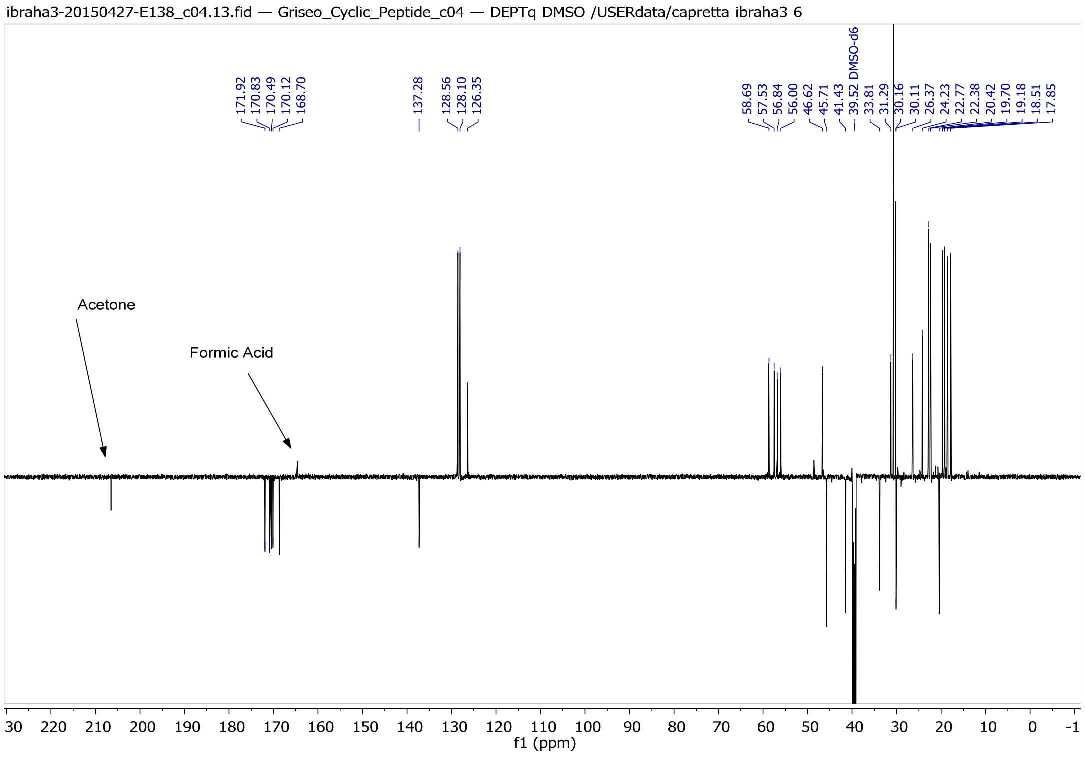

Figure 19S. ^{13}C -DEPTq spectrum of ellisiiamide B (176 MHz, DMSO $_{d6}$).

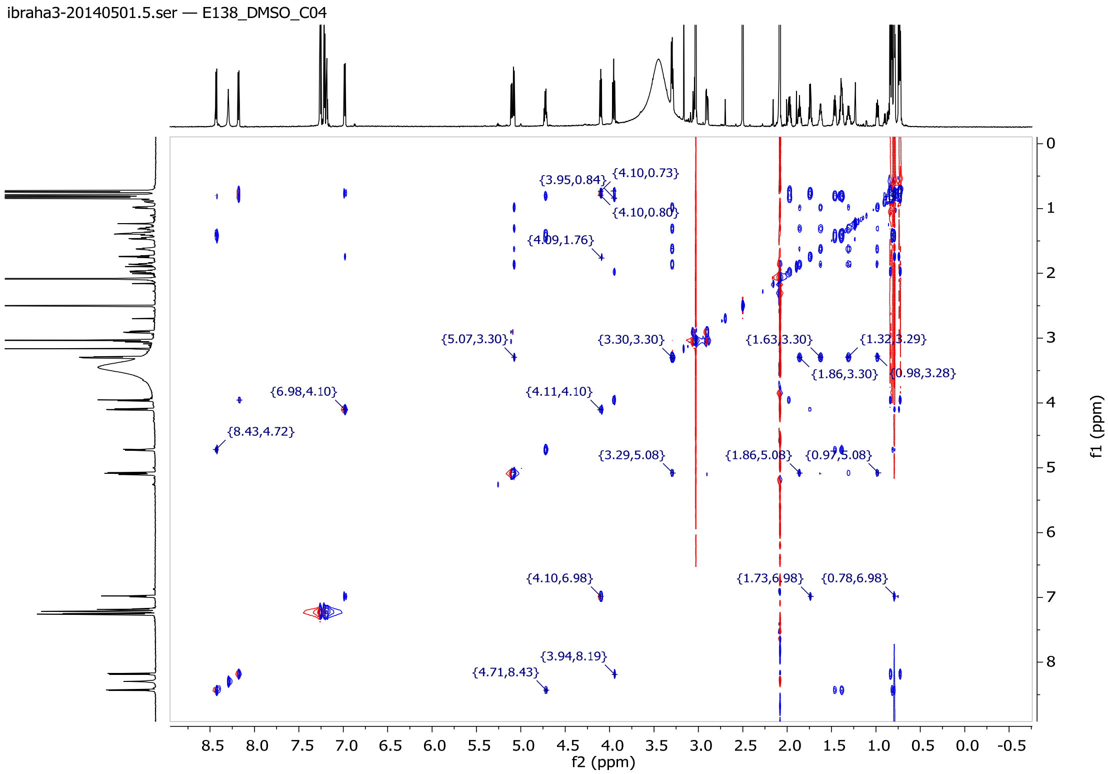

Figure 20S. TOCSY spectrum of ellisiiamide B (850 MHz, DMSO $_{d6}$).

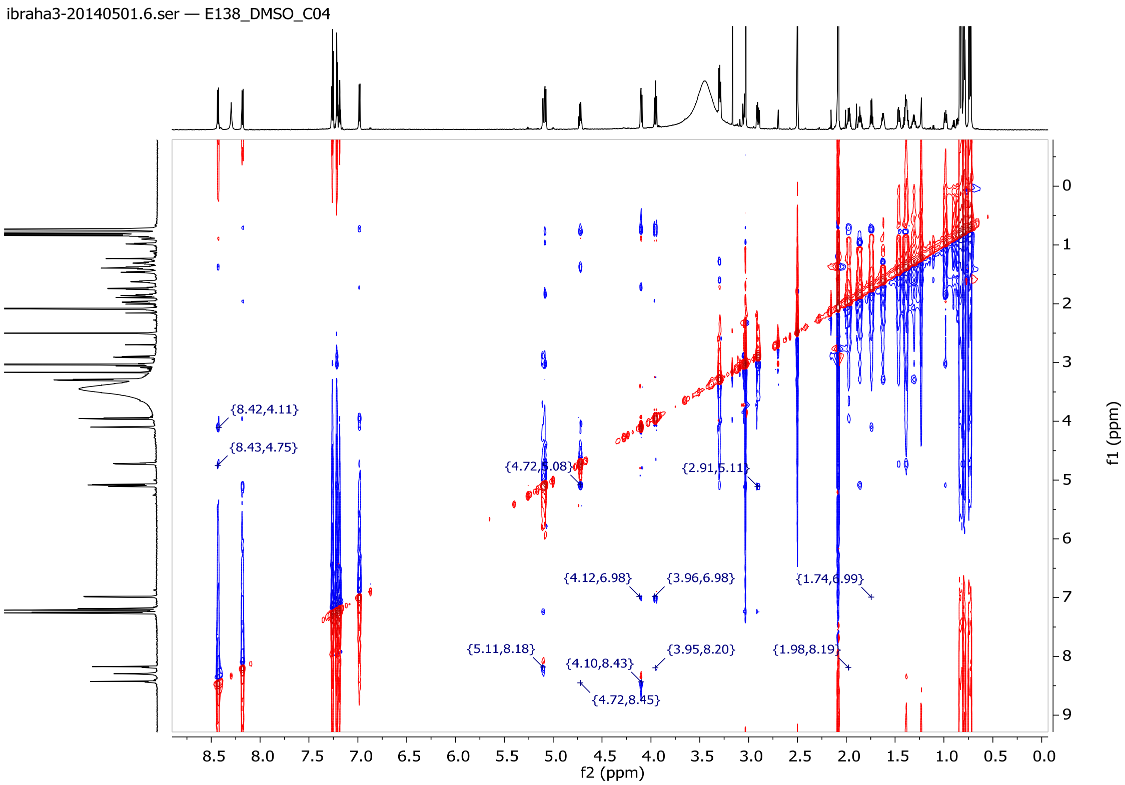

Figure 21S. ROESY spectrum of ellisiiamide B (850 MHz, DMSO $_{d6}$).

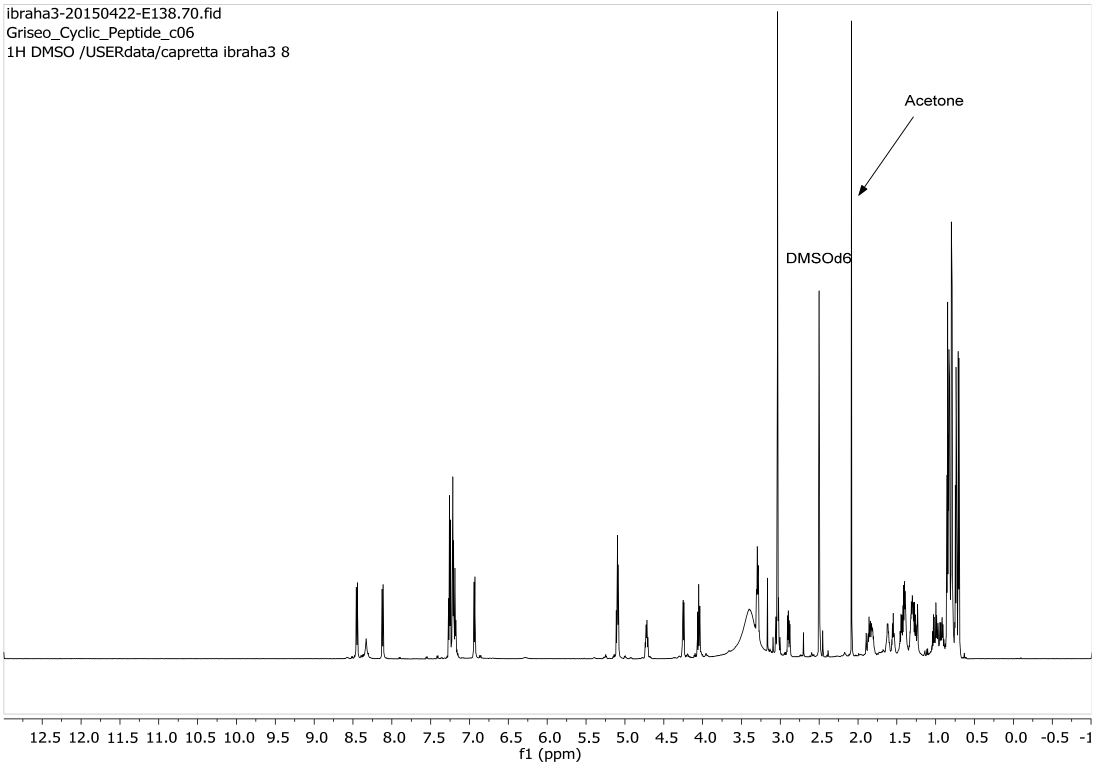

Figure 22S. ^{1}H spectrum of ellisiiamide C (700 MHz, DMSO $_{d6}$).

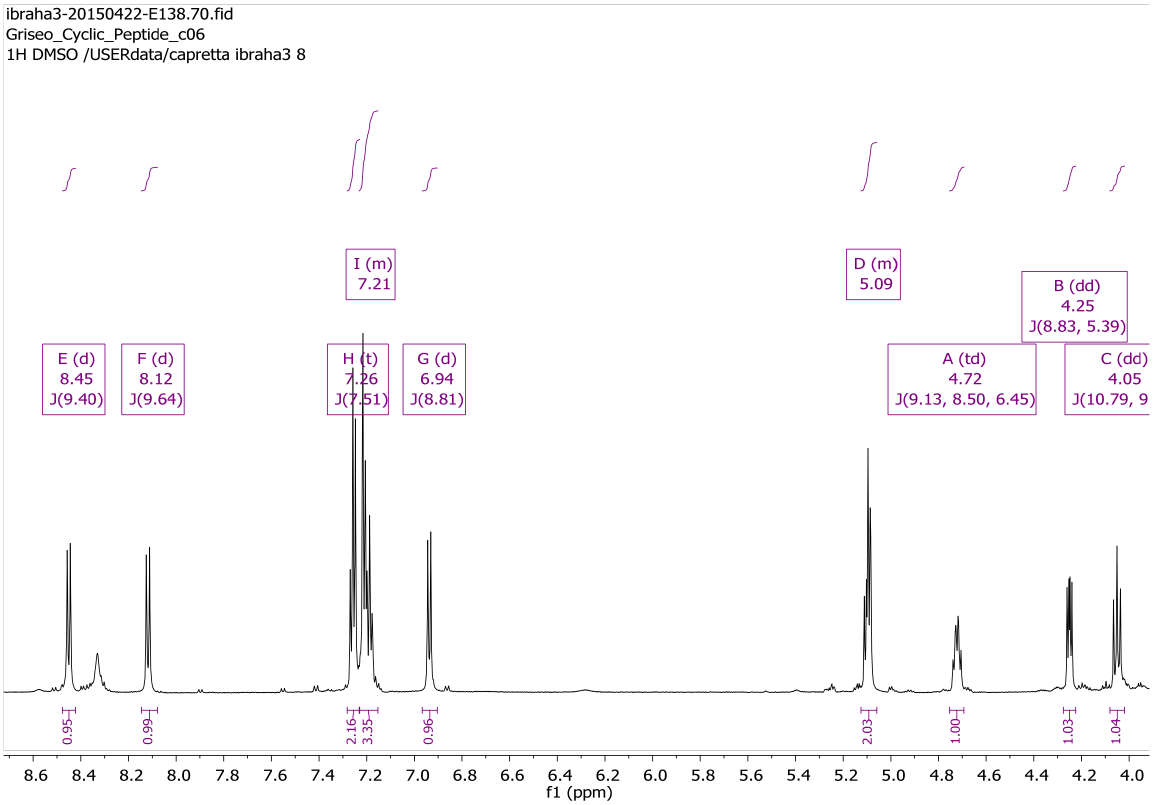

Figure 23S. Expanded ^{1}H spectrum of ellisiiamide C (700 MHz, DMSO $_{d6}$).

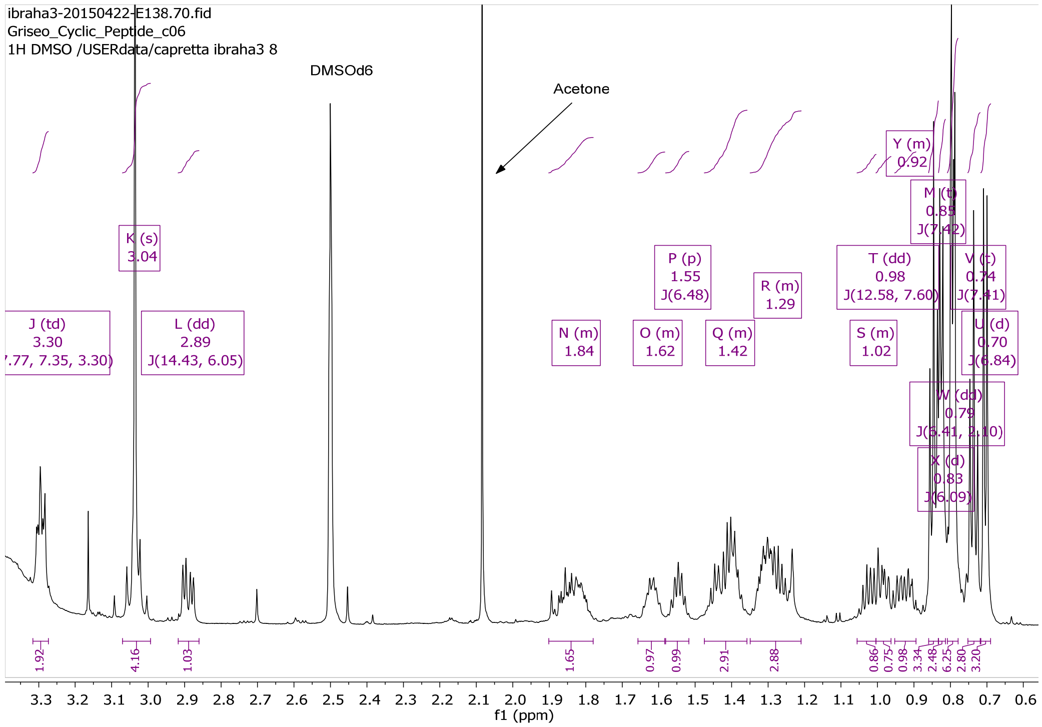

Figure 24S. Expanded ^{1}H spectrum of ellisiiamide C (700 MHz, DMSO $_{d6}$).

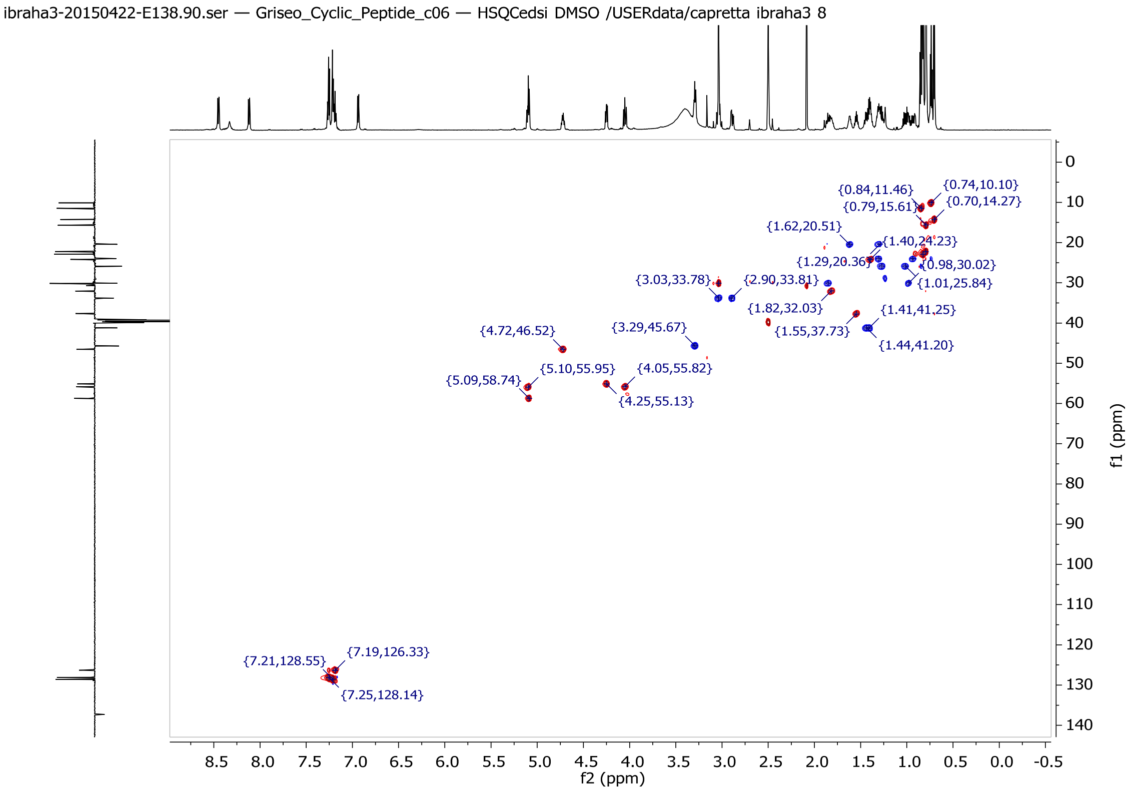

Figure 25S. HSQC spectrum of ellisiiamide C (700 MHz, DMSO $_{d6}$), multiplicity edited.

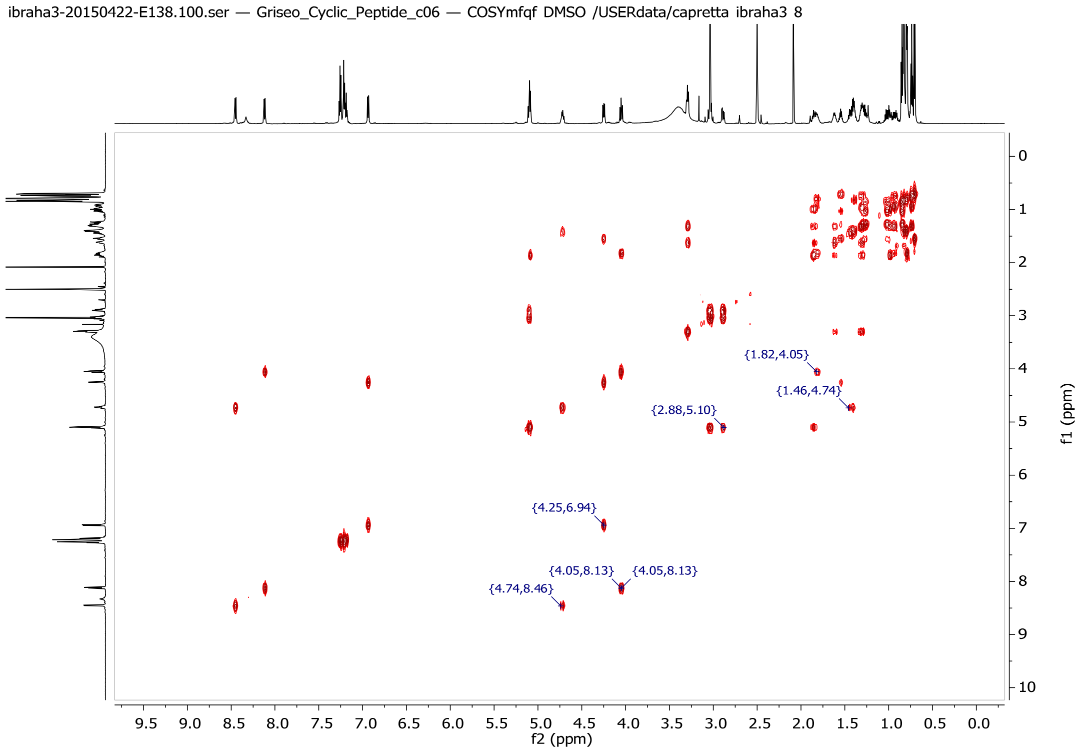

Figure 26S. COSY spectrum of ellisiiamide C (700 MHz, DMSO $_{d6}$).

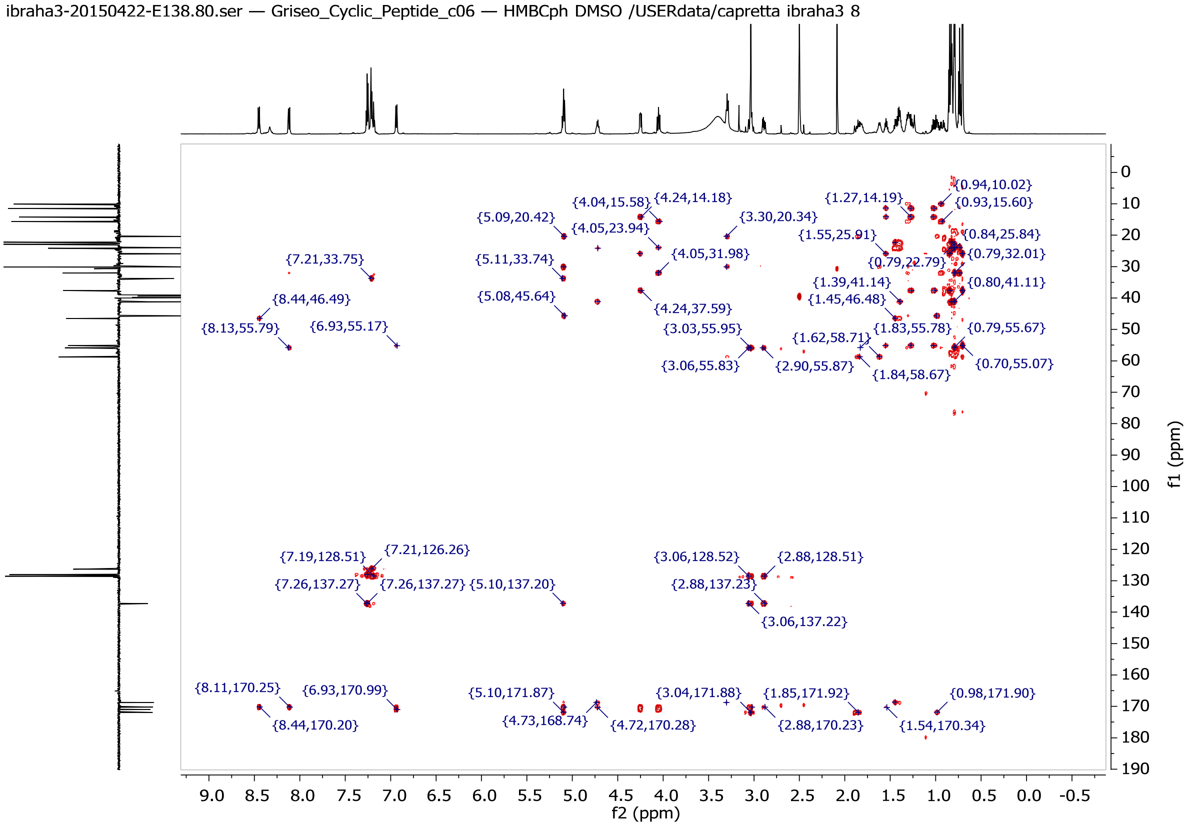

Figure 27S. HMBC spectrum of ellisiiamide C (700 MHz, DMSO $_{d6}$).

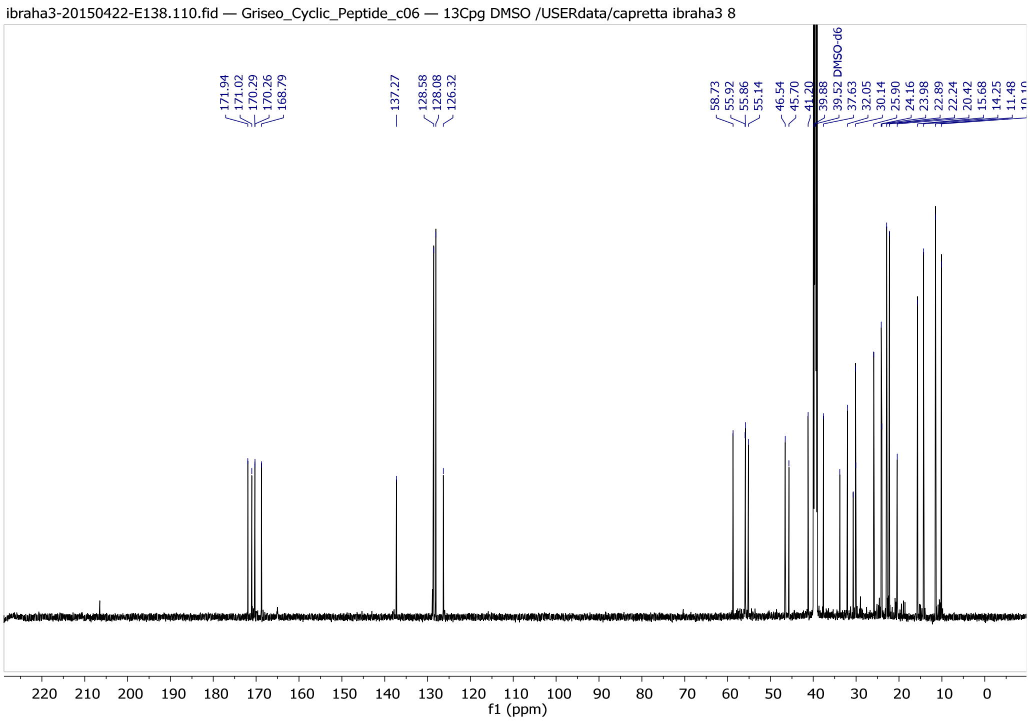

Figure 28S. ^{13}C spectrum of ellisiiamide C (176 MHz, DMSO $_{d6}$).

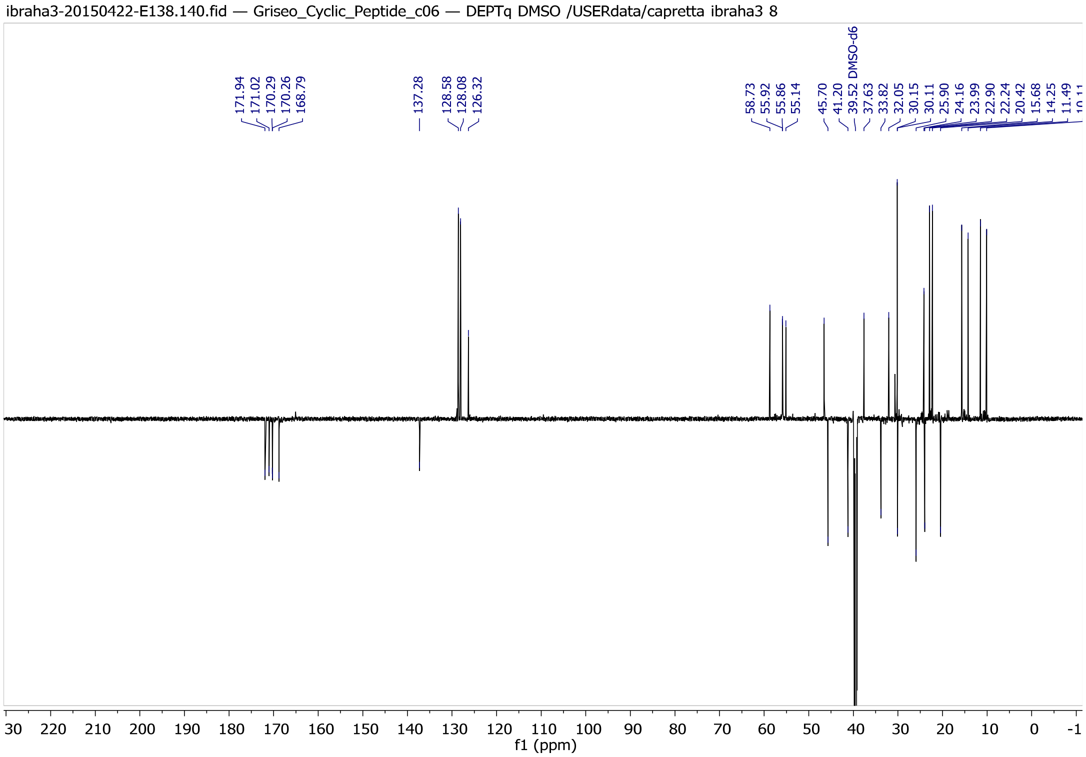

Figure 29S. ^{13}C -DEPTq spectrum of ellisiiamide C (176 MHz, DMSO $_{d6}$).

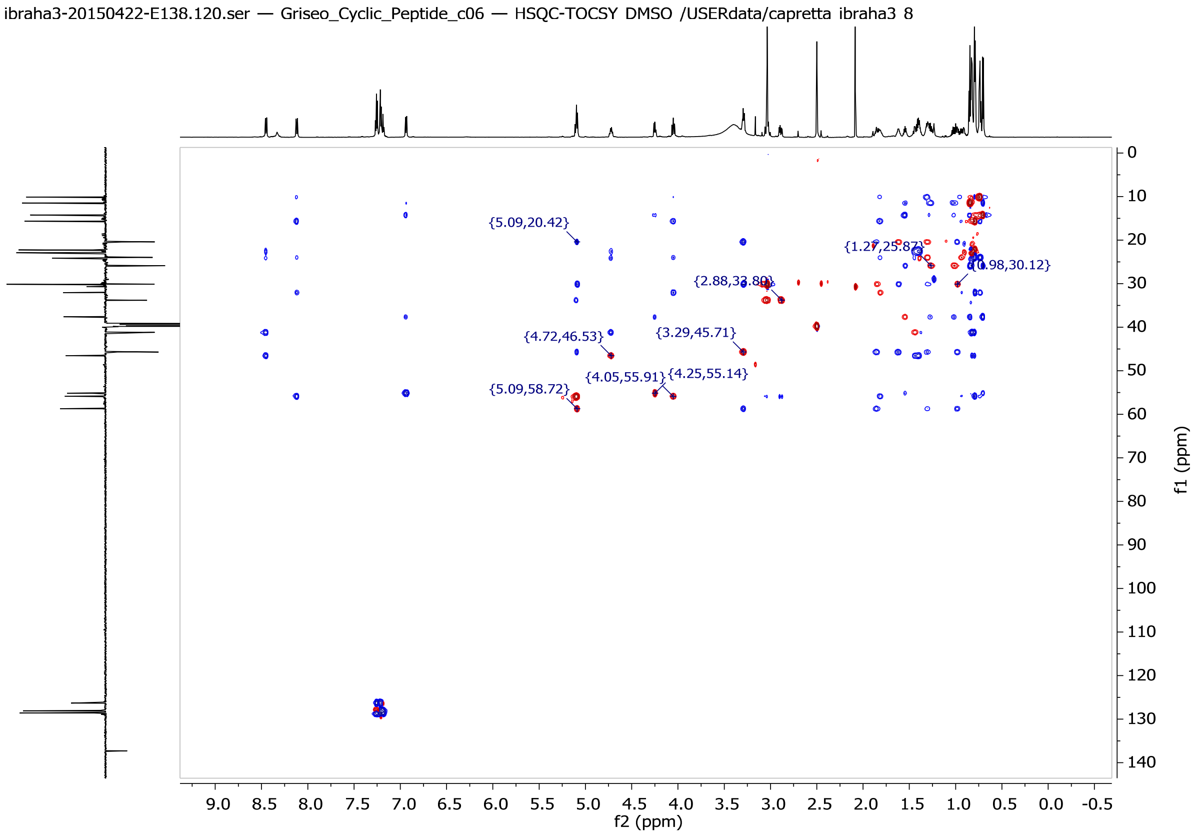

Figure 30S. HSQC-TOCSY spectrum of ellisiiamide C (700 MHz, DMSO $_{d6}$).

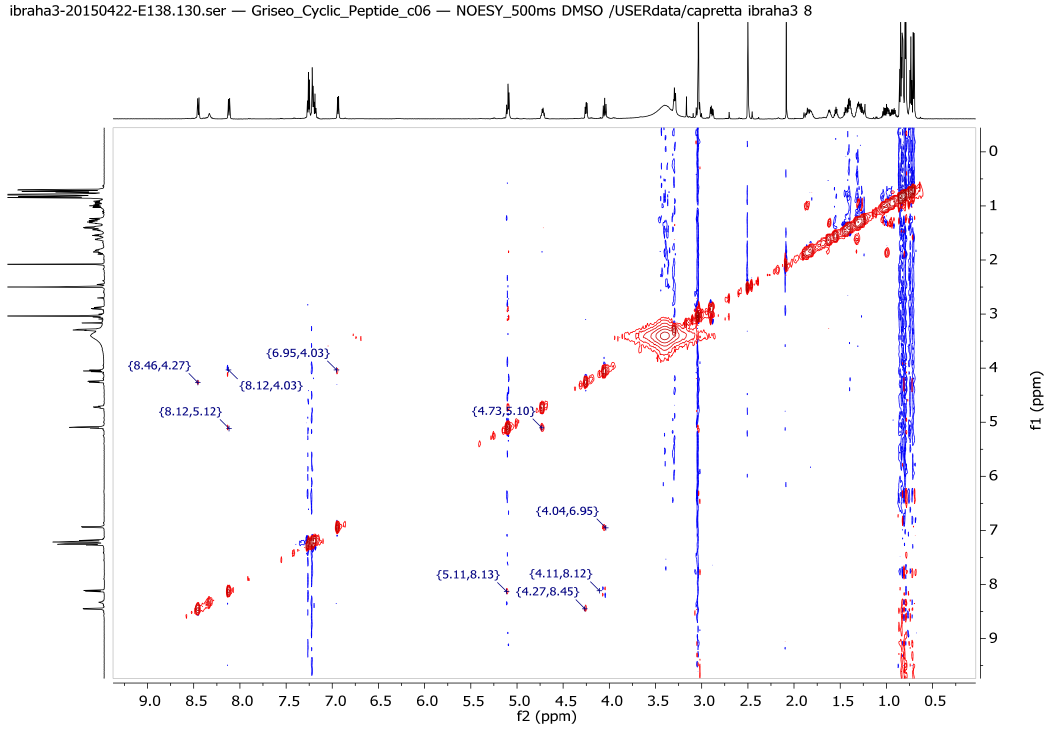

Figure 31S. NOESY spectrum of ellisiiamide C (700 MHz, DMSO $_{d6}$).

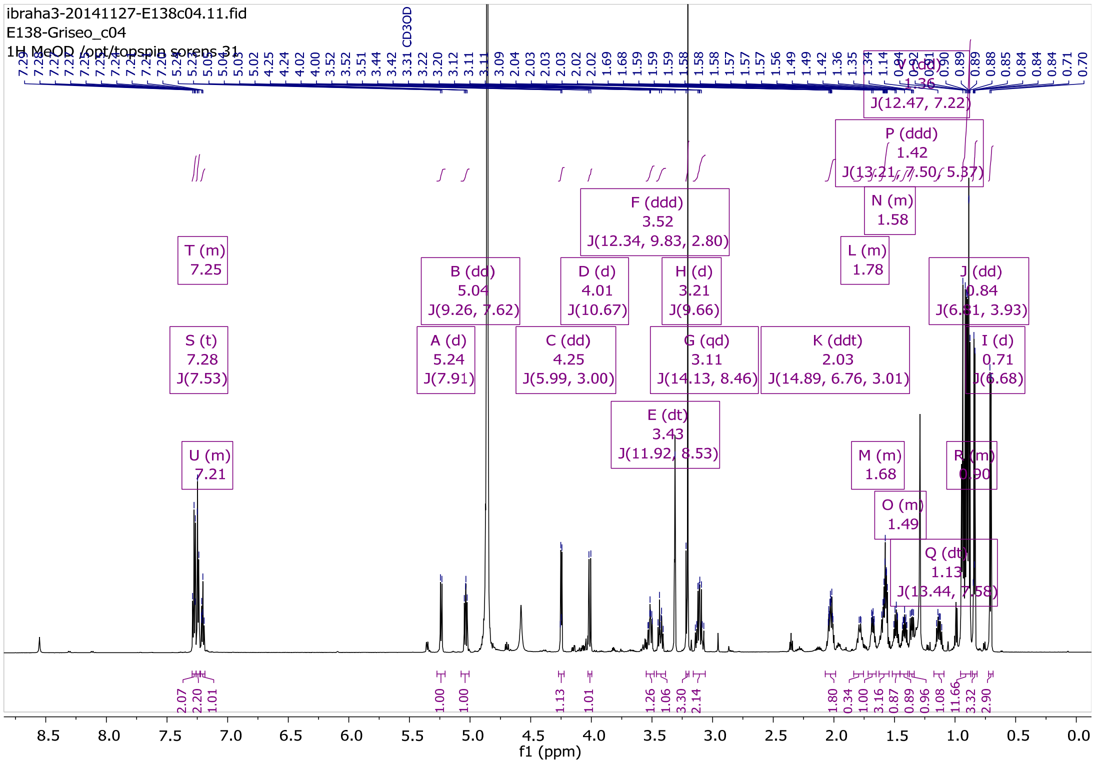

Figure 32S. ^{1}H spectrum of cyclic pentapeptide 1 (700 MHz, CD_3OD).

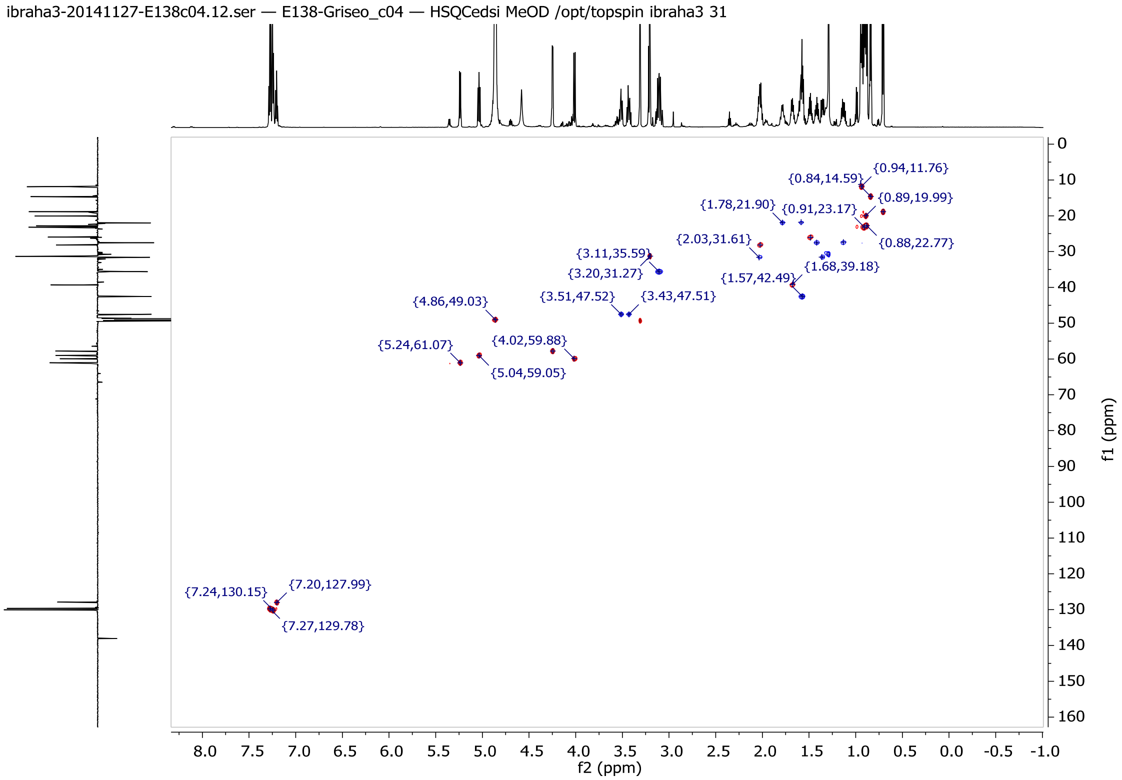

Figure 33S. HSQC spectrum of cyclic pentapeptide 1 (700 MHz, CD_3OD).

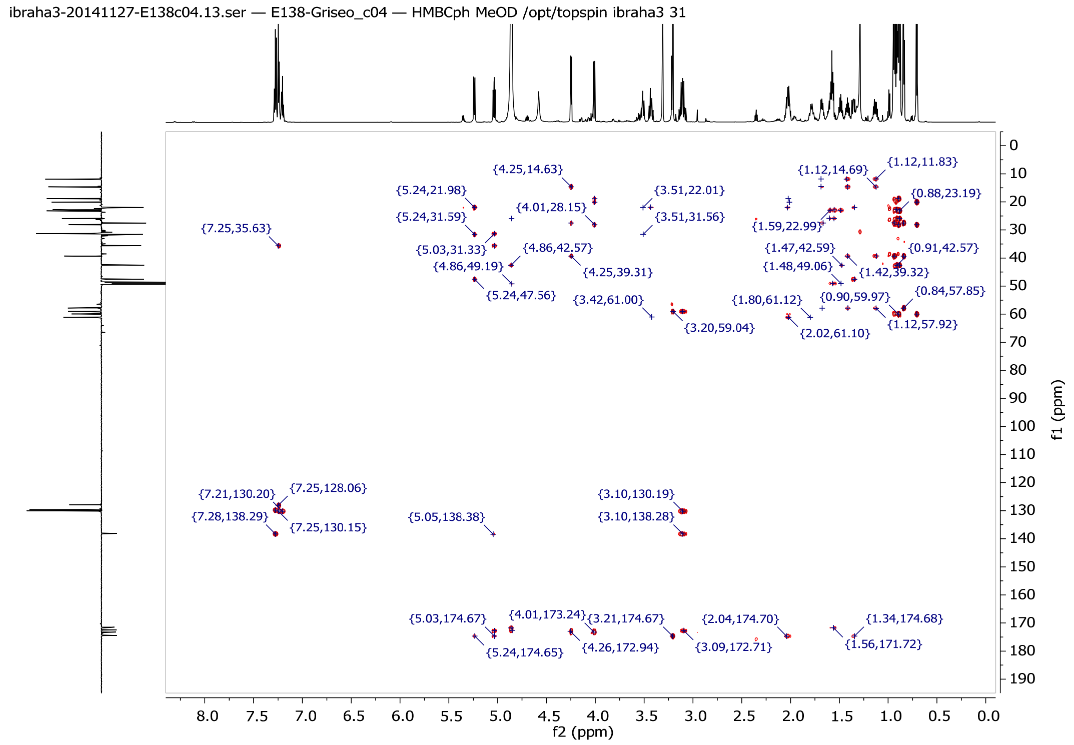

Figure 34S. HMBC spectrum of cyclic pentapeptide 1 (700 MHz, CD_3OD).

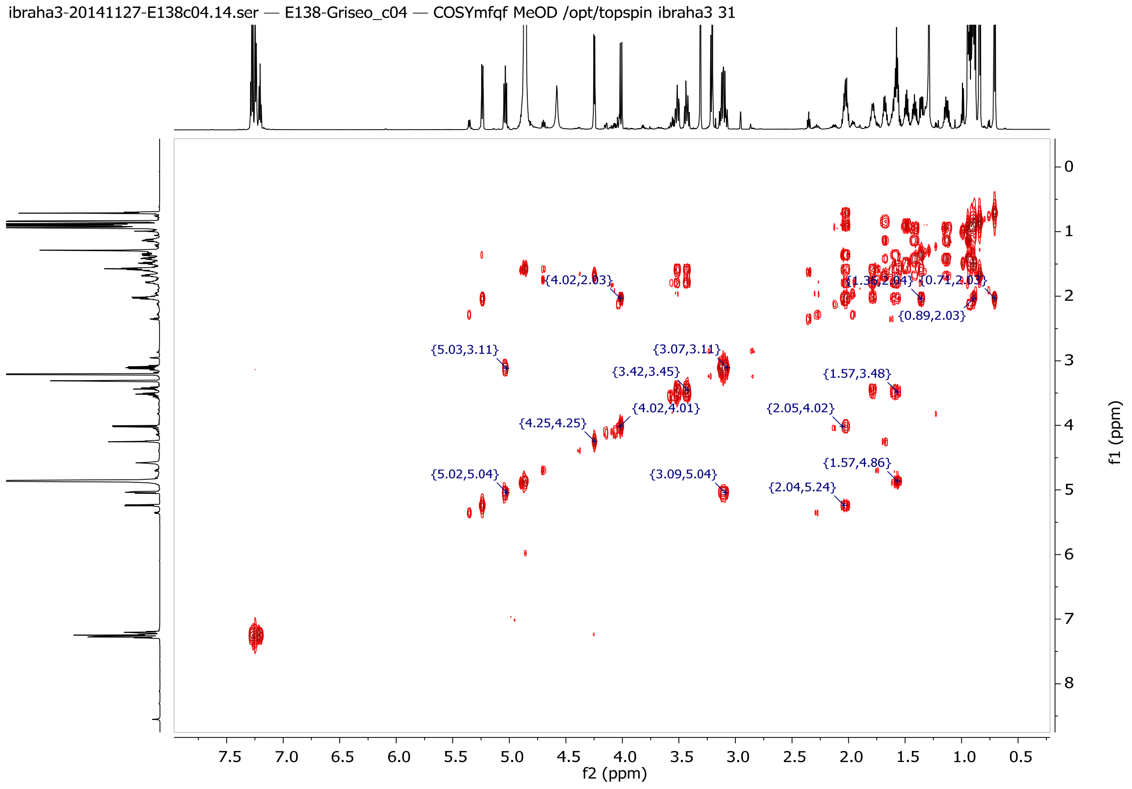

Figure 35S. COSY spectrum of cyclic pentapeptide 1 (700 MHz, CD_3OD).

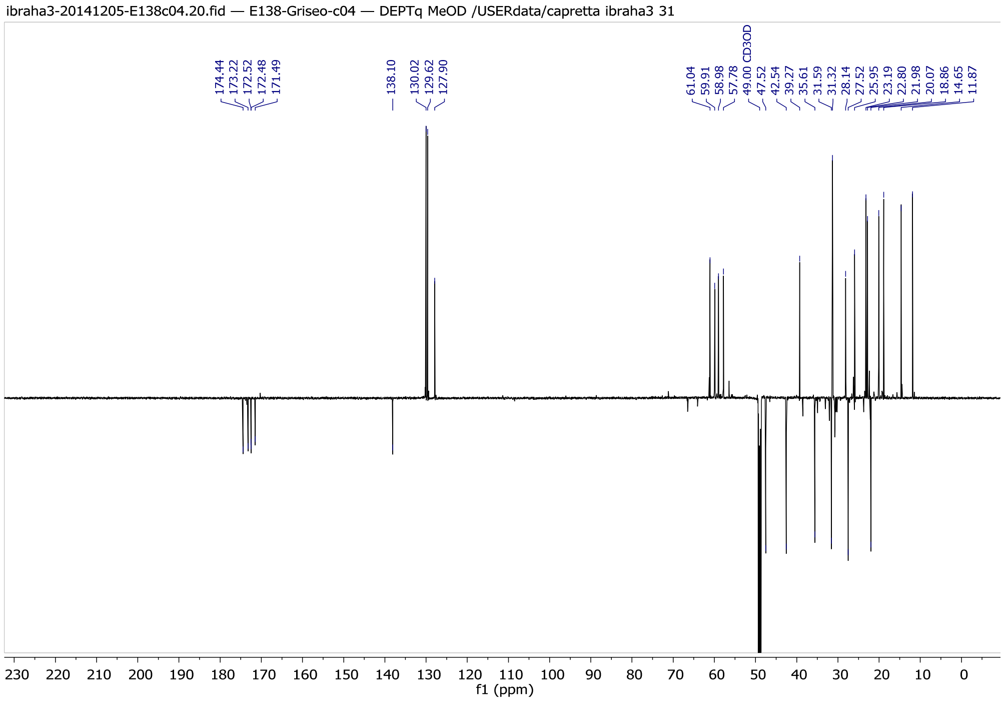

Figure 36S. ^{13}C -DEPTq spectrum of cyclic pentapeptide 1 (176 MHz, CD_3OD).

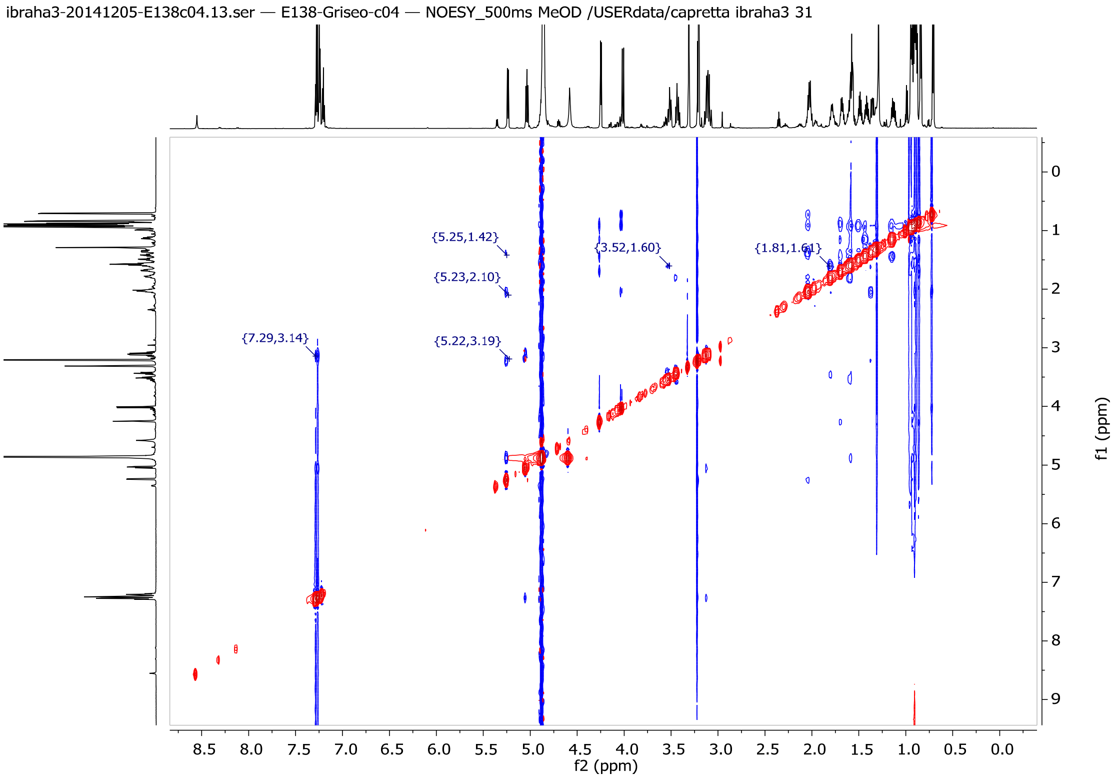

Figure 37S. NOESY spectrum of cyclic pentapeptide 1 (700 MHz, $DMSO_{d6}$).

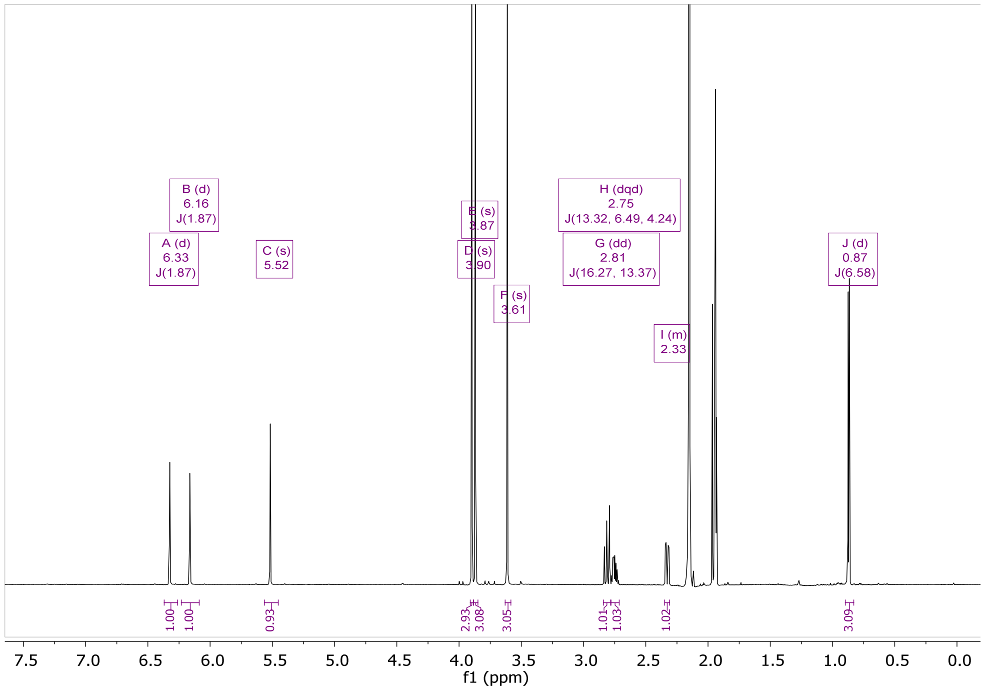

Figure 38S. ^{1}H spectrum of dechlorogriseofulvin (700 MHz, CD_3CN).

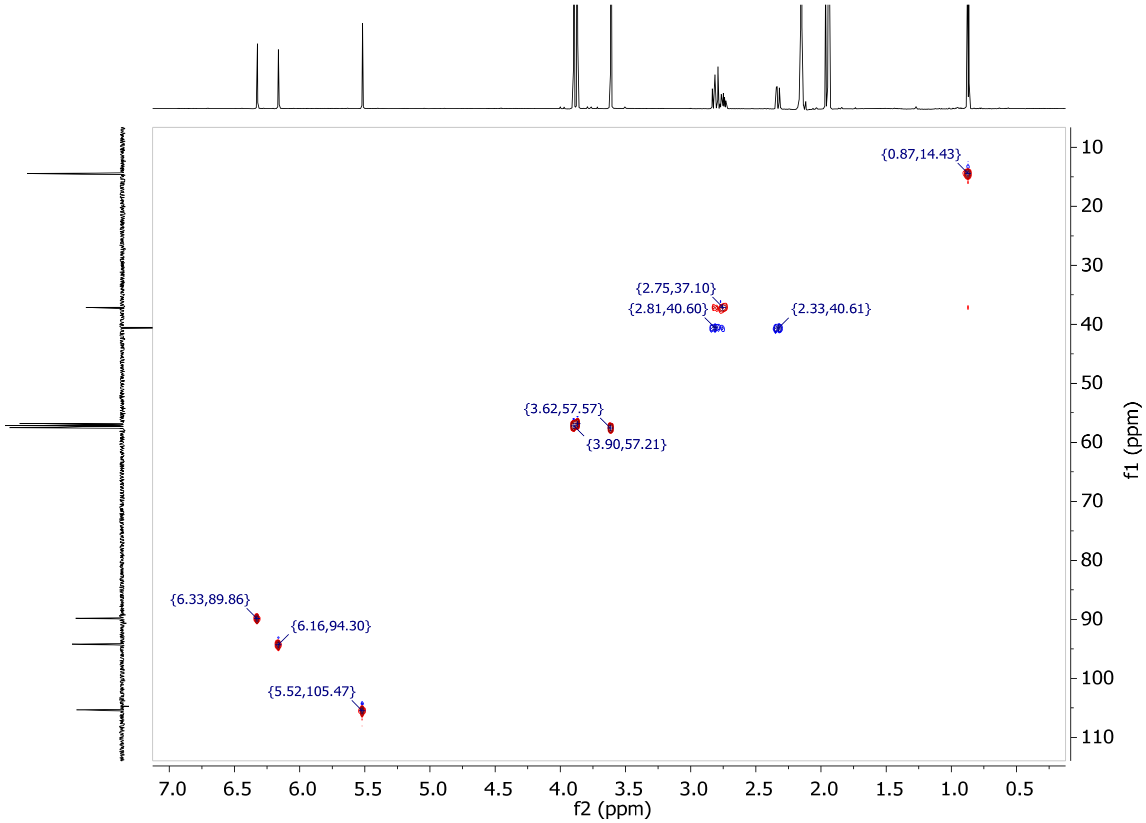

Figure 39S. HSQC spectrum of dechlorogriseofulvin (700 MHz, CD_3CN).

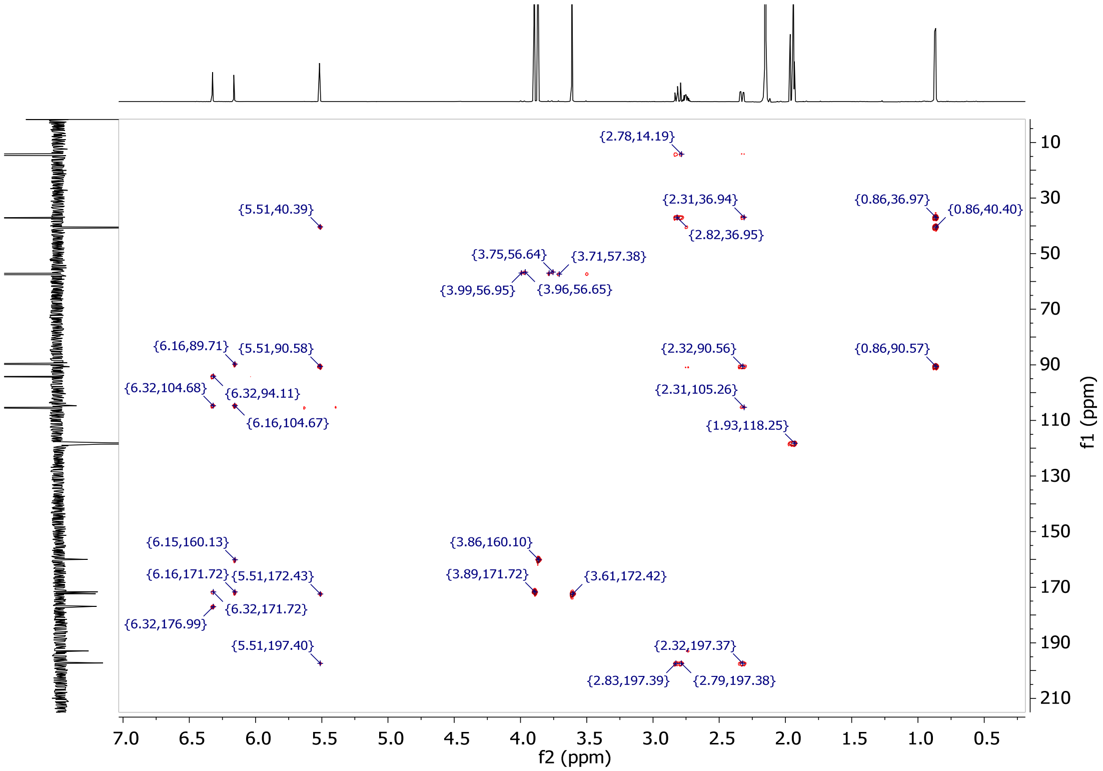

Figure 40S. HMBC spectrum of dechlorogriseofulvin (700 MHz, CD_3CN).

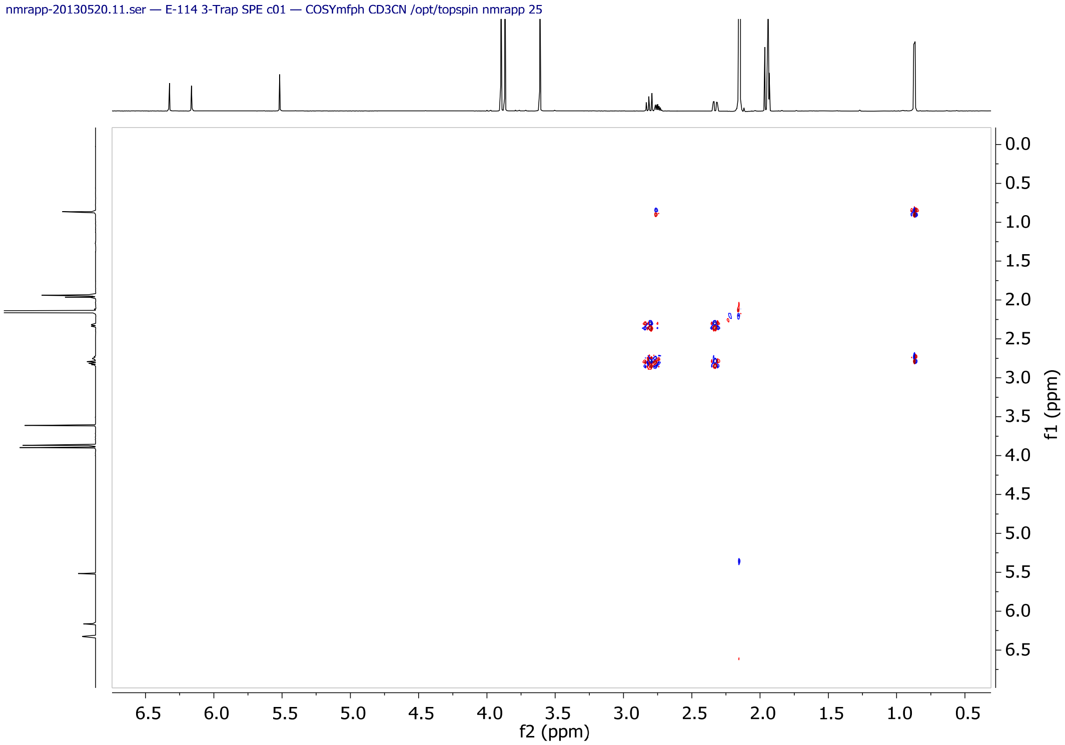

Figure 41S. COSY spectrum of dechlorogriseofulvin (700 MHz, CD_3CN).

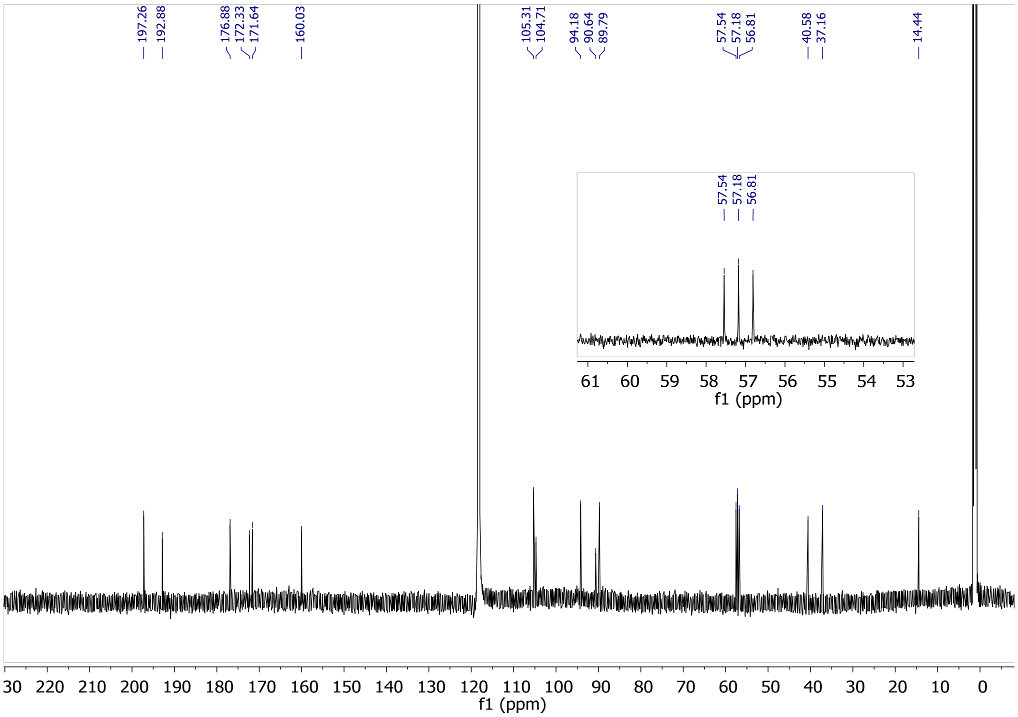

Figure 42S. ^{13}C spectrum of dechlorogriseofulvin (176 MHz,CD_3CN).

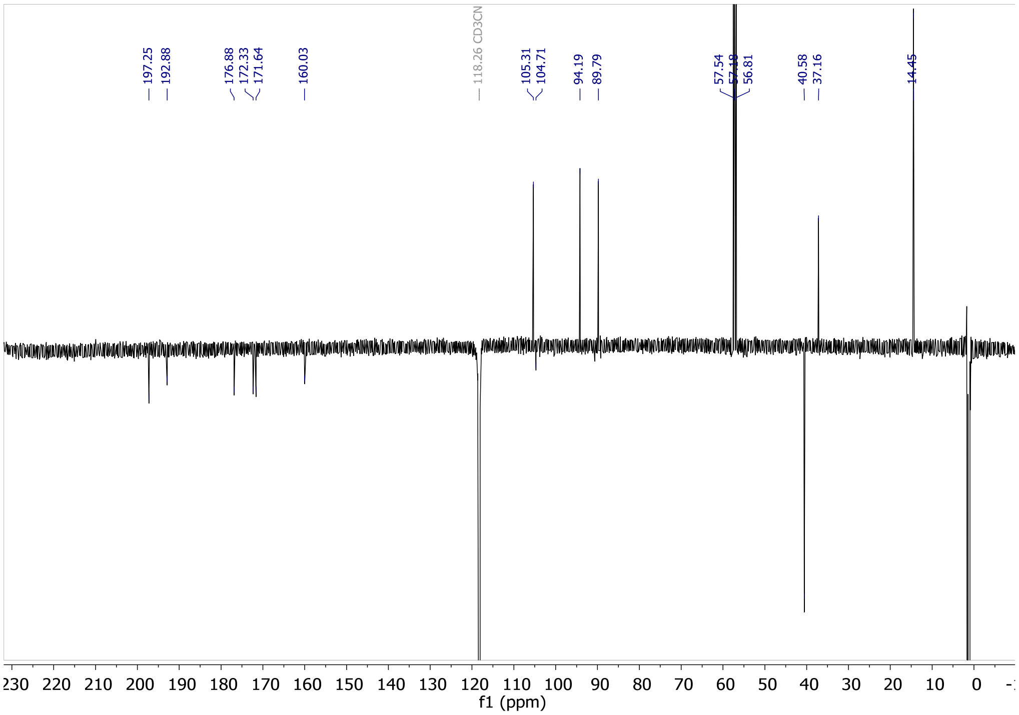

Figure 43S. ^{13}C -DEPTq spectrum o of dechlorogriseofulvin (176 MHz, CD_3CN).

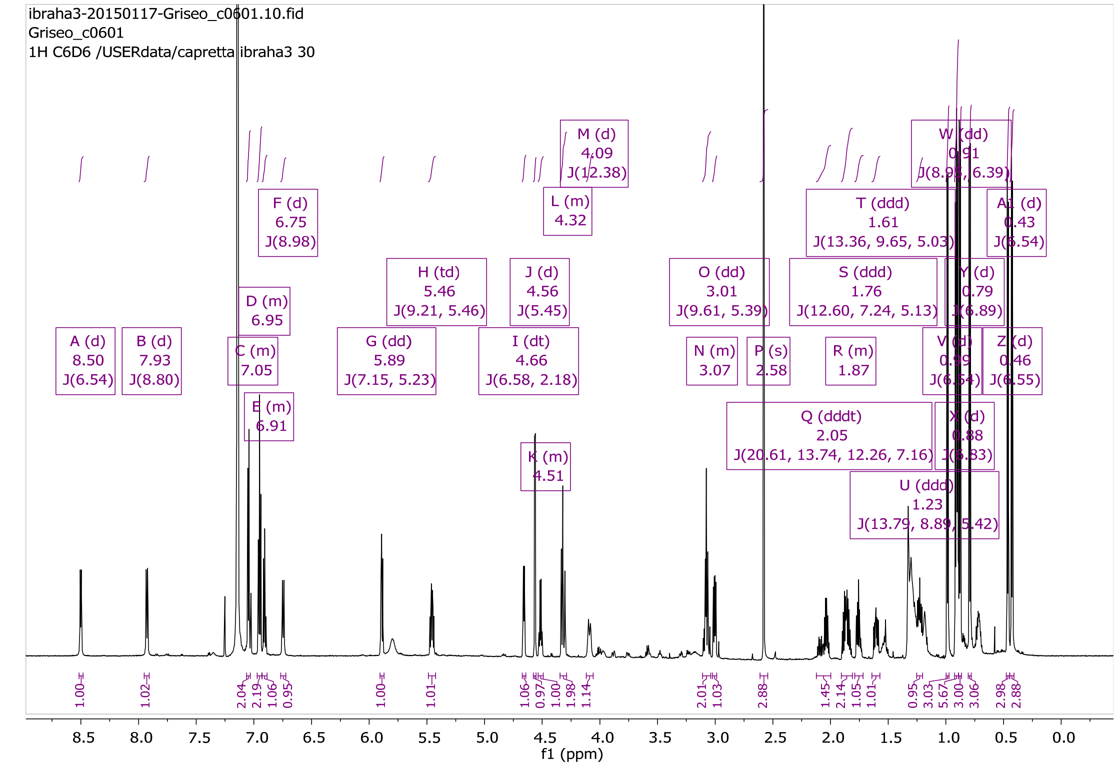

Figure 44S. ^{1}H spectrum of Hirsutatin A (700 MHz, C6D6).

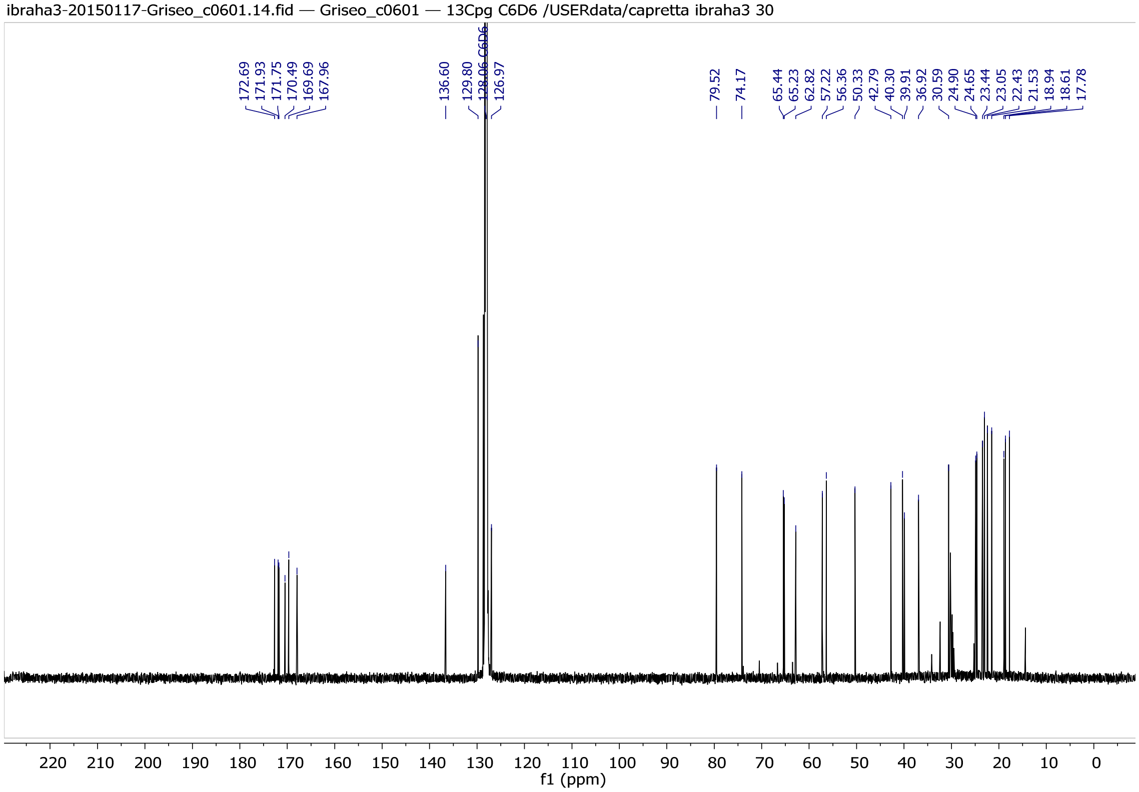

Figure 45S. ^{13}C spectrum of Hirsutatin A (176 MHz, C6D6).

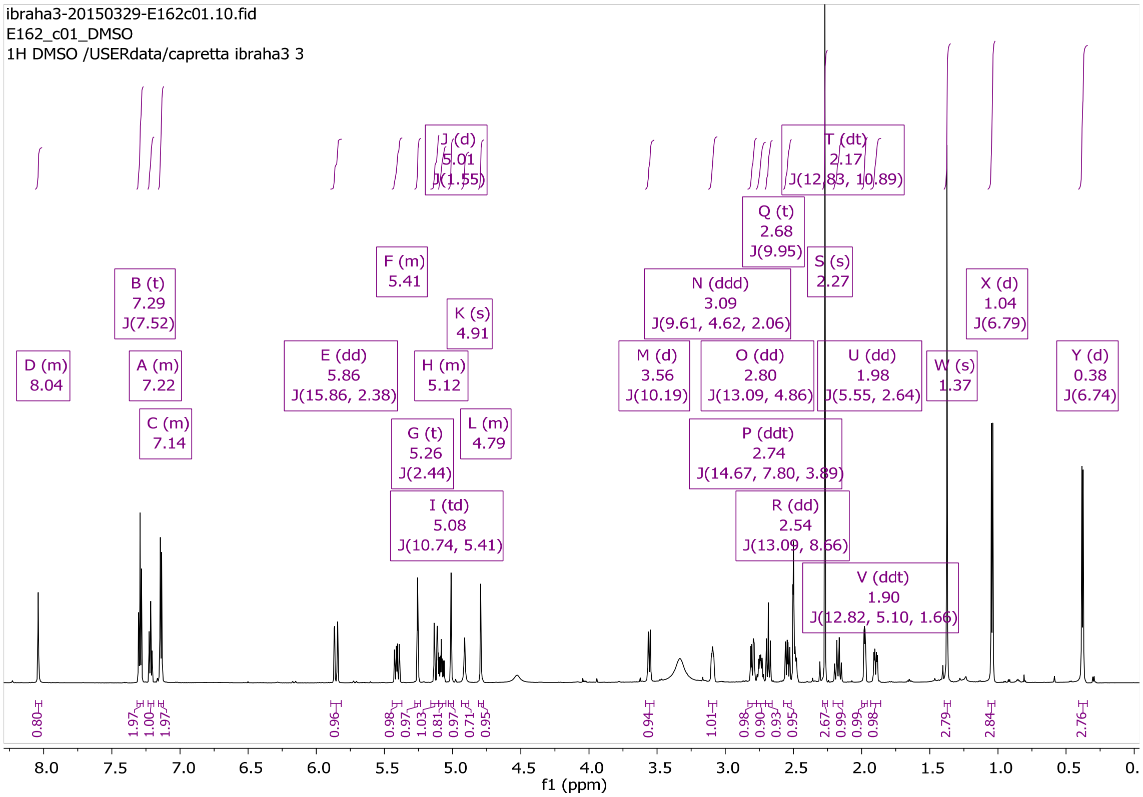

Figure 46S. ^{1}H spectrum of Cytochalasin D (700 MHz, $DMSO_{d6}$).

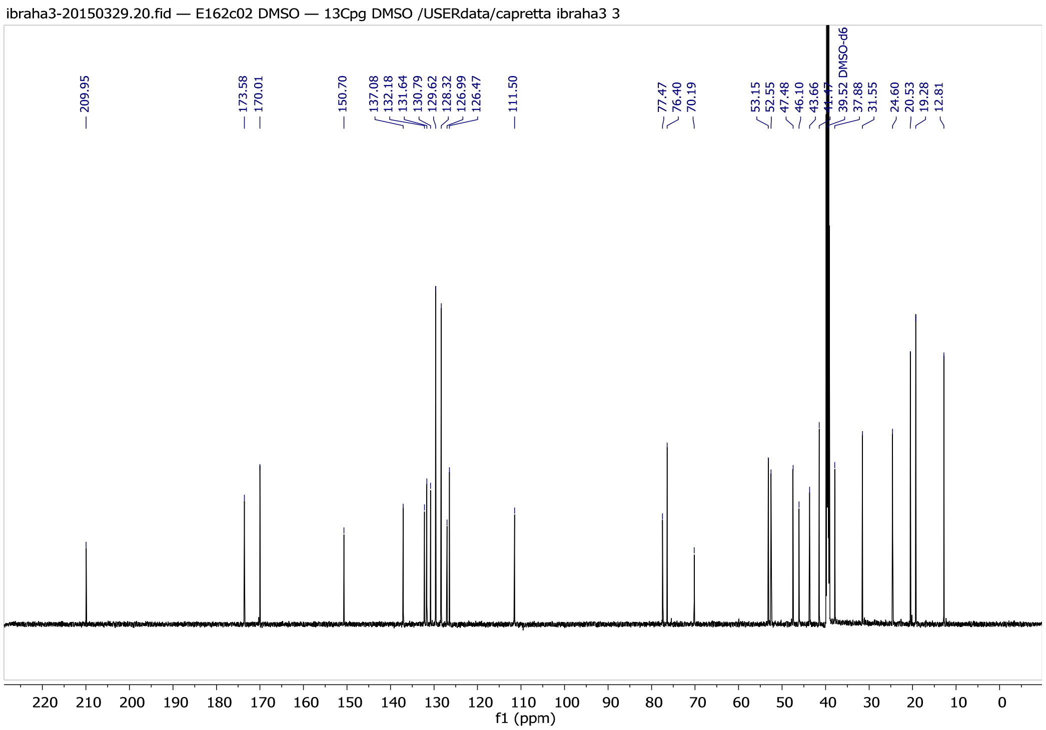

Figure 47S. ^{13}C spectrum of Cytochalasin D (176 MHz, $DMSO_{d6}$).

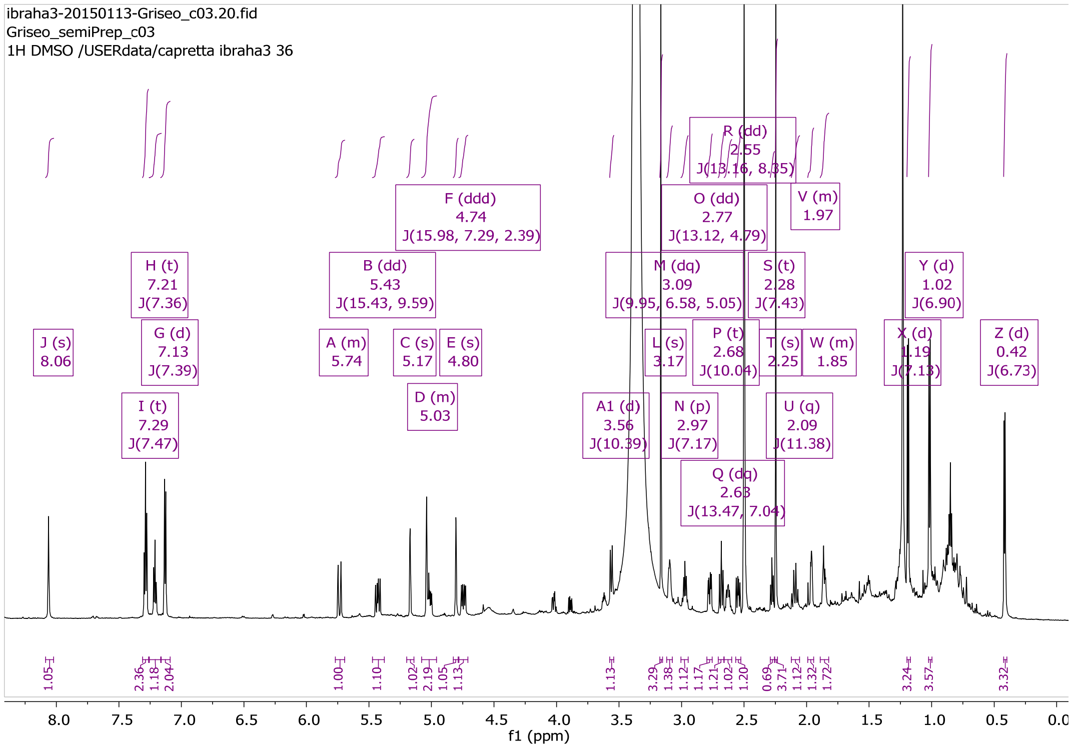

Figure 48S. ^{1}H spectrum of Zygosporin E (700 MHz, DMSO$_{d6}$).

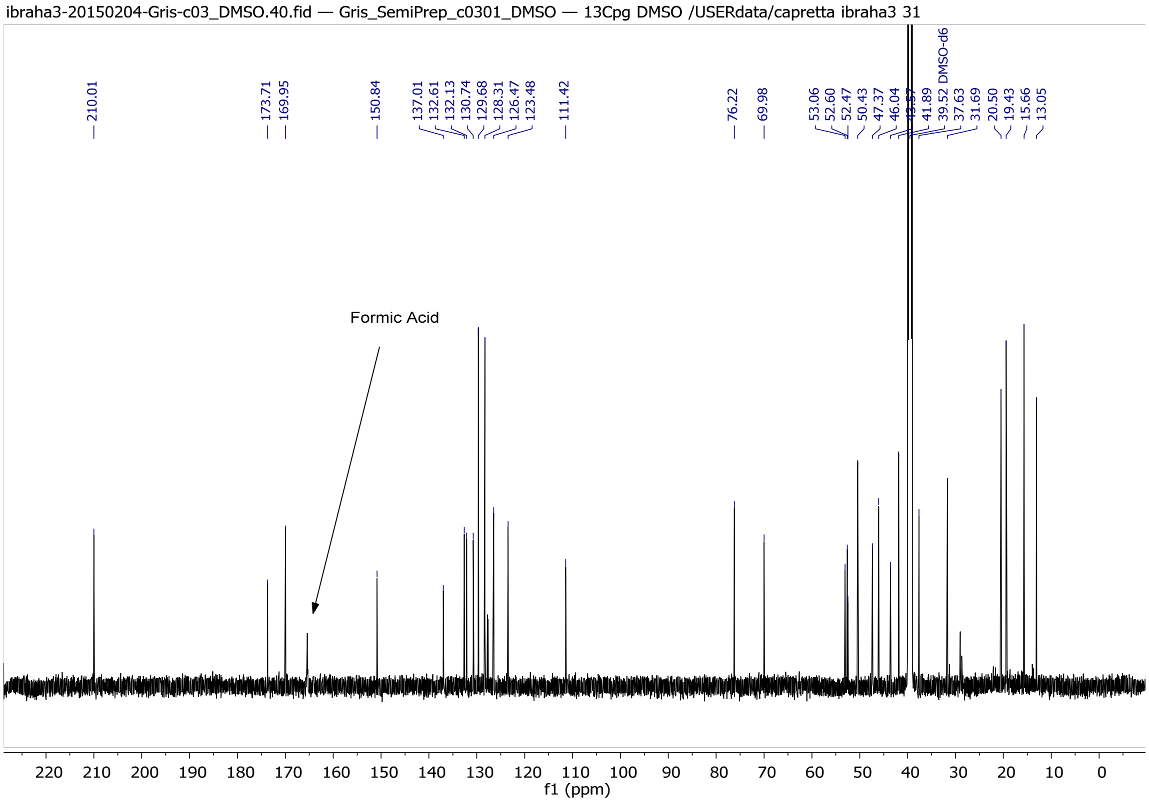

Figure 49S. ^{13}C spectrum of Zygosporin E (176 MHz, DMSO$_{d6}$).

Supplementary Table 13S. Sequences generated and used in the phylogenetic analyses in this study.

GenBank ID	Voucher No.	GenBank Accession No.			Host/substrate	Reference
		ITS	LSU	RPB2		
Amphirosellinia nigrospora	HAST:91092308[T]	—	—	GQ848340	Dead twigs	Hsieh et al. (2010)
Barrmaelia rhamnicola	CBS:142772[T]	—	—	MF488999	Dead branch of *Rhamnus alpina*	Voglmayr et al. (2018)
Fungal endophyte sp.	1988	EU687008	—	—	*Lithachne pauciflora* endophyte	Higgins et al. (2011)
Fungal endophyte sp.	Beach leaf mycelium 1	AB041994	—	—	*Fagus crenata* leaf litter	unpublished
Fungal endophyte sp.	ECD-2008 isolate 22	EU685974	—	—	*Metzgeria furcata* endophyte	Davis & Shaw (2008)
Fungal endophyte sp.	P1512A	EU977315	—	—	endophyte	Smith et al. (2008)
Fungal endophyte sp.	P1804A	EU977234	—	—	endophyte	Smith et al. (2008)
Fungal sp.	ARIZ:B225	FJ612962	—	—	*Cecropia insignis* seed endophyte	U'Ren et al. (2009)
Fungal sp.	ARIZ:B492	FJ613077	—	—	*Cecropia insignis* seed endophyte	U'Ren et al. (2009)
Fungal sp.	ARIZ:L192	FJ612722	—	—	*Cecropia insignis* seed endophyte	U'Ren et al. (2009)
Fungal sp.	FEX13	JN051363	—	—	*Mikania laevigata* endophyte	Ribeiro et al. (2012)
Nemania serpens	HAST:235	GU292820	—	GQ844773	Soil	Hsieh et al. (2010)
Stilbohypoxylon quisquiliarum	HAST:89091608	—	—	GQ853021	Bark	Hsieh et al. (2010)
Stilbohypoxylon quisquiliarum	JDR:172	—	—	GQ853020	Bark	Hsieh et al. (2010)
Xylaria acuminatilongissima	HAST:623[T]	—	—	GQ853028	Ground of bamboo plantation	Hsieh et al. (2010)
Xylaria allantoidea	HAST:94042903	—	—	GQ848356	Trunk	Hsieh et al. (2010)
Xylaria atrosphaerica	HAST:91111214	—	—	GQ848342	Bark of *Machilus thunbergii*	Hsieh et al. (2010)
Xylaria badia	HAST:95070101	—	—	GQ844833	Culm of bamboo	Hsieh et al. (2010)
Xylaria bambusicola	WSP:205[T]	—	—	GQ844802	Culms of *Bambusa oldhamii*	Hsieh et al. (2010)
Xylaria berteri	90112623	GU324749	—	—	bark	Hsieh et al. (2010)
Xylaria berteri	C28	JQ936291	—	—	*Glycine max* endophyte	de Souza Leite et al. (2013)
Xylaria berteri	C2P24A	JQ936297	—	—	*Glycine max* endophyte	de Souza Leite et al. (2013)
Xylaria berteri	C32	JQ936294	—	—	*Glycine max* endophyte	de Souza Leite et al. (2013)
Xylaria berteri	C80	JQ936295	—	—	*Glycine max* endophyte	de Souza Leite et al. (2013)
Xylaria berteri	HAST:90112623	—	—	GQ848362	Bark	Hsieh et al. (2010)
Xylaria berteri	JDR:256	—	—	GQ848363	Bark	Hsieh et al. (2010)
Xylaria berteri	R10	KP133344	—	—	—	Thomas et al. (2016)
Xylaria brunneovinosa	HAST:720[T]	—	—	GQ853023	Ground of bamboo plantation	Hsieh et al. (2010)
Xylaria castorea	DAOM:86019	MN219587	—	—	—	This study

GenBank ID	Voucher No.	GenBank Accession No.			Host/substrate	Reference
		ITS	LSU	RPB2		
Xylaria castorea	ICMP:18798	JN225908	—	—	*Nothofagus solandri* endophyte	Johnston et al. (2012)
Xylaria castorea	PDD:600	GU324751	—	GQ853018	undetermined tree	Hsieh et al. (2010)
Xylaria cf. *castorea*	HAST:91092303	—	—	GQ853019	Bark	Hsieh et al. (2010)
Xylaria cf. *heliscus*	ARIZ:FL0217	JQ760015	—	—	*Selaginella arenicola* endolichen	U'Ren et al. (2012)
Xylaria cf. *heliscus*	ARIZ:FL0414	JQ760150	—	—	*Herpothallon rubrocinctum* endolichen	U'Ren et al. (2012)
Xylaria cf. *heliscus*	ARIZ:FL0505	JQ760224	—	—	*Usnea mutabilis* endolichen	U'Ren et al. (2012)
Xylaria cf. *heliscus*	ARIZ:FL1070	JQ760693	—	—	*Usnea mutabilis* endolichen	U'Ren et al. (2012)
Xylaria cf. *heliscus*	ARIZ:FL1274	JQ760889	—	—	*Cladonia leporina* endolichen	U'Ren et al. (2012)
Xylaria cf. *heliscus*	ARIZ:FL1295	JQ760910	—	—	*Cladonia leporina* endolichen	U'Ren et al. (2012)
Xylaria cf. *heliscus*	ARIZ:FL1754	KU683894	—	—	dead *Quercus inopina* leaf	U'Ren et al. (2016)
Xylaria cf. *heliscus*	ARIZ:NC0263	JQ761303	—	—	*Xanthoparmelia conspersa* endolichen	U'Ren et al. (2012)
Xylaria cf. *heliscus*	ARIZ:NC0265	JQ761304	—	—	*Sticta beauvoisii* endolichen	U'Ren et al. (2012)
Xylaria cf. *heliscus*	ARIZ:NC0277	JQ761315	—	—	*Hypnum* sp. endophyte	U'Ren et al. (2012)
Xylaria cf. *heliscus*	ARIZ:NC0364	JQ761396	—	—	*Peltigera praetextata* endolichen	U'Ren et al. (2012)
Xylaria cf. *heliscus*	ARIZ:NC0385	JQ761415	—	—	*Flavoparmelia caperata* endolichen	U'Ren et al. (2012)
Xylaria cf. *heliscus*	ARIZ:NC0390	JQ761420	—	—	*Lecanora oreinoides* endolichen	U'Ren et al. (2012)
Xylaria cf. *heliscus*	ARIZ:NC0572	JQ761561	—	—	*Hypnum* sp. endophyte	U'Ren et al. (2012)
Xylaria cf. *heliscus*	ARIZ:NC0607	JQ761595	—	—	*Lasallia pennsylvanica* endolichen	U'Ren et al. (2012)
Xylaria cf. *heliscus*	ARIZ:NC1096	JQ761742	—	—	*Diploschistes scruposus* endolichen	U'Ren et al. (2012)
Xylaria cf. *heliscus*	ARIZ:NC1163	JQ761807	—	—	*Tsuga canadensis* endophyte	U'Ren et al. (2012)
Xylaria cf. *heliscus*	ARIZ:NC1223	JQ761861	—	—	*Tsuga canadensis* endophyte	U'Ren et al. (2012)
Xylaria cf. *heliscus*	HAST:88113010	—	—	GQ848355	Bark	Hsieh et al. (2010)
Xylaria corniformis	DAOM:145381	MN219588	—	—	*Acer* sp. wood	This study
Xylaria corniformis	DAOM:159095	MN219591	—	—	*Acer* sp. wood	This study
Xylaria corniformis	DAOM:159097	MN219590	—	—	*Acer* sp. wood	This study
Xylaria corniformis	DAOM:159115	MN219589	—	—	*Acer* sp. wood	This study
Xylaria crozonensis	HAST:398	—	—	GQ848361	Bark of *Quercus* sp.	Hsieh et al. (2010)
Xylaria cubensis	GENT:159	—	—	GQ853017	dead decorticated wood	Hsieh et al. (2010)
Xylaria cubensis	HAST:477	—	—	GQ848364	Log	Hsieh et al. (2010)
Xylaria cubensis	HAST:515	—	—	GQ848366	Bark	Hsieh et al. (2010)
Xylaria cubensis	JDR:860	—	—	GQ848365	Wood	Hsieh et al. (2010)
Xylaria curta	HAST:494	—	—	GQ844831	Dead wood	Hsieh et al. (2010)

GenBank ID	Voucher No.	GenBank Accession No.			Host/substrate	Reference
		ITS	LSU	RPB2		
Xylaria curta	HAST:92092022	—	—	GQ844830	Bark	Hsieh et al. (2010)
Xylaria digitata	HAST:919	GU322456	—	GQ848338	Wood	Hsieh et al. (2010)
Xylaria discolor	HAST:131023[T]	—	—	JQ087411	On wood of *Ocotea foetens*	Ju et al. (2012)
Xylaria ellisii	DAOM:242568	KJ504358	—	—	*Pinus strobus* endophyte	Richardson et al. (2014)
Xylaria ellisii	DAOMC: 252031[T]	MN218820	MN218817	MN216186	Stromata on decaying *Acer saccharum* branch	This study
Xylaria ellisii	NB-437-5E	MN218821	MN218816	MN216183	*Picea rubens* endophyte	This study
Xylaria ellisii	NB-487-5B	MN219584	—	—	*Picea rubens* endophyte	This study
Xylaria ellisii	NB-487-5C	MN219586	—	—	*Picea rubens* endophyte	This study
Xylaria ellisii	NB-488-6L	MN218822	MN218818	MN216184	*Picea rubens* endophyte	This study
Xylaria ellisii	NB-505-4D	MN219585	—	—	*Picea rubens* endophyte	This study
Xylaria ellisii	NB-505-5H	MN218823	MN218819	MN216185	*Picea rubens* endophyte	This study
Xylaria ellisii	NB-746	MN219731	—	—	Stromata on decaying *Acer saccharum* branch	This study
Xylaria ellisii	RLG 6407	KT289568	—	—	*Umbilicaria mammulata* endolichen	Arnold et al. (2009)
Xylaria enterogena	HAST:785	—	—	GQ848349	Wood	Hsieh et al. (2010)
Xylaria frustulosa	HAST:771	—	—	GQ844837	Bark	Hsieh et al. (2010)
Xylaria frustulosa	HAST:92092010	—	—	GQ844838	Bark	Hsieh et al. (2010)
Xylaria globosa	HAST:775	—	—	GQ848348	Bark	Hsieh et al. (2010)
Xylaria haemorrhoidalis	HAST:89041207	—	—	GQ848347	Bark	Hsieh et al. (2010)
Xylaria hypoxylon	CBS:122620[T]	—	—	KY624231	Old tree stump of *Sorbus aucuparia*	Wendt et al. (2018)
Xylaria intracolorata	HAST:90080402	—	—	GQ848354	Bark	Hsieh et al. (2010)
Xylaria laevis	95072910	GU324747	—	—	bark	Hsieh et al. (2010)
Xylaria laevis	CEQCA-M1296	KC771495	—	—	*Piper* sp. endophyte	unpublished
Xylaria laevis	HAST:419	GU324746	—	GQ848359	Dead wood	Hsieh et al. (2010)
Xylaria laevis	HAST:95072910	—	—	GQ848360	Bark	Hsieh et al. (2010)
Xylaria laevis	INBio:1215B	KU204446	—	—	*Weinmannia burserifolia* endophyte	unpublished
Xylaria longipes	DAOM:214176	MN219592	—	—	—	This study
Xylaria luteostromata var. *macrospora*	HAST:508	—	—	GQ848352	Dead wood	Hsieh et al. (2010)
Xylaria montagnei	HAST:495	—	—	GQ848337	Dead wood	Hsieh et al. (2010)
Xylaria ophiopoda	HAST:93082805	—	—	GQ848344	Bark	Hsieh et al. (2010)
Xylaria plebeja	HAST:91122401	GU324740	—	GQ848353	Trunk of *Machilus zuihoensis*	Hsieh et al. (2010)
Xylaria polymorpha	HAST:1012	—	—	GQ848343	Wood	Hsieh et al. (2010)

GenBank ID	Voucher No.	GenBank Accession No.			Host/substrate	Reference
		ITS	LSU	RPB2		
Xylaria polymorpha	MUCL:49884[T]	—	—	KY624288	Wood	Wendt et al. (2018)
Xylaria polymorpha	NBRC 30369	AB274817	—	—	termite nest	Okane et al. (2007)
Xylaria primorskensis	478	FJ707473	—	—	log	Ju et al. (2009)
Xylaria primorskensis	1-HL2-1	KT192367	—	—	*Platycladus orientalis* endophyte	Unpublished
Xylaria primorskensis	NF-G	JX160062	—	—	biofilm on lead sheeting	Rhee et al. (2014)
Xylaria regalis	HAST:92072001	—	—	GQ848357	Bark	Hsieh et al. (2010)
Xylaria schweinitzii	HAST:92092023	—	—	GQ848346	Bark	Hsieh et al. (2010)
Xylaria scruposa	HAST:497	—	—	GQ848341	Dead wood	Hsieh et al. (2010)
Xylaria sp.	CBSC II-A	AB743839	—	—	*Cinchona pubescens* endophyte	Shibuya et al. (2003)
Xylaria sp.	D2b1a	JQ341065	—	—	*Diospyros crassiflora* endophyte	Douanla-Meli & Langer (2012)
Xylaria sp.	D4b3b	JQ341069	—	—	*Diospyros crassiflora* endophyte	Douanla-Meli & Langer (2012)
Xylaria sp.	Davis CD368	AY315404	—	—	*Trichocolea tomentella* endophyte	Davis et al. (2003)
Xylaria sp.	E10214a	JN418792	—	—	*Euterpe precatoria* endophyte	Unpublished
Xylaria sp.	E7407c	HM855211	—	—	*Piper hispidum* endophyte	Unpublished
Xylaria sp.	Fungal endophyte isolate 1555	KR015035	—	—	*Gnetum gnemon* endophyte	Vincent et al. (2016)
Xylaria sp.	Fungal endophyte isolate 225	KR015187	—	—	*Ficus pungens* endophyte	Vincent et al. (2016)
Xylaria sp.	Fungal endophyte isolate 2475	KR015270	—	—	*Ficus variegata* endophyte	Vincent et al. (2016)
Xylaria sp.	Fungal endophyte isolate 2511	KR015293	—	—	*Ficus variegata* endophyte	Vincent et al. (2016)
Xylaria sp.	Fungal endophyte isolate 2759	KR015336	—	—	*Macaranga punctata* endophyte	Vincent et al. (2016)
Xylaria sp.	Fungal endophyte isolate 2831	KR015343	—	—	*Ficus variegata* endophyte	Vincent et al. (2016)
Xylaria sp.	Fungal endophyte isolate 2837	KR015345	—	—	*Macaranga punctata* endophyte	Vincent et al. (2016)
Xylaria sp.	Fungal endophyte isolate 3189	KR015401	—	—	*Syzygium longipes* endophyte	Vincent et al. (2016)
Xylaria sp.	Fungal endophyte isolate 3930	KR015675	—	—	*Ficus variegata* endophyte	Vincent et al. (2016)
Xylaria sp.	Fungal endophyte isolate 3993	KR015691	—	—	*Macaranga punctata* endophyte	Vincent et al. (2016)
Xylaria sp.	Fungal endophyte isolate 4005	KR015698	—	—	*Macaranga punctata* endophyte	Vincent et al. (2016)
Xylaria sp.	Fungal endophyte isolate 4024	KR015709	—	—	*Macaranga punctata* endophyte	Vincent et al. (2016)
Xylaria sp.	Fungal endophyte isolate 4151	KR015729	—	—	*Macaranga punctata* endophyte	Vincent et al. (2016)
Xylaria sp.	Fungal endophyte isolate 4251	KR015747	—	—	*Ficus pungens* endophyte	Vincent et al. (2016)
Xylaria sp.	Fungal endophyte isolate 4267	KR015753	—	—	*Ficus pungens* endophyte	Vincent et al. (2016)
Xylaria sp.	Fungal endophyte isolate 4282	KR015759	—	—	*Ficus variegata* endophyte	Vincent et al. (2016)
Xylaria sp.	Fungal endophyte isolate 4365	KR015775	—	—	*Ficus variegata* endophyte	Vincent et al. (2016)
Xylaria sp.	Fungal endophyte isolate 4366	KR015776	—	—	*Ficus variegata* endophyte	Vincent et al. (2016)

GenBank ID	Voucher No.	GenBank Accession No.			Host/substrate	Reference
		ITS	LSU	RPB2		
Xylaria sp.	Fungal endophyte isolate 4367	KR015777	—	—	*Ficus variegata* endophyte	Vincent et al. (2016)
Xylaria sp.	Fungal endophyte isolate 4493	KR015800	—	—	*Psychotria leptothyrsa* endophyte	Vincent et al. (2016)
Xylaria sp.	Fungal endophyte isolate 4496	KR015801	—	—	*Psychotria leptothyrsa* endophyte	Vincent et al. (2016)
Xylaria sp.	Fungal endophyte isolate 4969	KR015926	—	—	*Macaranga aleuritoides* endophyte	Vincent et al. (2016)
Xylaria sp.	Fungal endophyte isolate 5216	KR015983	—	—	*Macaranga bifoveata* endophyte	Vincent et al. (2016)
Xylaria sp.	Fungal endophyte isolate 5772	KR016089	—	—	*Macaranga bifoveata* endophyte	Vincent et al. (2016)
Xylaria sp.	Fungal endophyte isolate 6096	KR016247	—	—	*Macaranga aleuritoides* endophyte	Vincent et al. (2016)
Xylaria sp.	Fungal endophyte isolate 6131	KR016265	—	—	*Macaranga aleuritoides* endophyte	Vincent et al. (2016)
Xylaria sp.	Fungal endophyte isolate 6199	KR016313	—	—	*Ficus hahliana* endophyte	Vincent et al. (2016)
Xylaria sp.	Fungal endophyte isolate 6204	KR016317	—	—	*Macaranga bifoveata* endophyte	Vincent et al. (2016)
Xylaria sp.	Fungal endophyte isolate 6580	KR016488	—	—	*Psychotria micrococca* endophyte	Vincent et al. (2016)
Xylaria sp.	Fungal endophyte isolate 6677	KR016542	—	—	*Psychotria micrococca* endophyte	Vincent et al. (2016)
Xylaria sp.	Fungal endophyte isolate 6853	KR016613	—	—	*Macaranga fallacina* endophyte	Vincent et al. (2016)
Xylaria sp.	Fungal endophyte isolate 6956	KR016657	—	—	*Ficus pungens* endophyte	Vincent et al. (2016)
Xylaria sp.	Fungal endophyte isolate 6980	KR016676	—	—	*Ficus pungens* endophyte	Vincent et al. (2016)
Xylaria sp.	Fungal endophyte isolate 6997	KR016686	—	—	*Syzygium longipes* endophyte	Vincent et al. (2016)
Xylaria sp.	Fungal endophyte isolate 6999	KR016688	—	—	*Syzygium longipes* endophyte	Vincent et al. (2016)
Xylaria sp.	Fungal endophyte isolate 7055	KR016722	—	—	*Ficus hahliana* endophyte	Vincent et al. (2016)
Xylaria sp.	Fungal endophyte isolate 7097	KR016743	—	—	*Psychotria micrococca* endophyte	Vincent et al. (2016)
Xylaria sp.	G30	JQ623492	—	—	*Garcinia hombroniana* endophyte	Unpublished
Xylaria sp.	IP-29	DQ780446	—	—	endophyte	Promputtha et al. (2007)
Xylaria sp.	IP-93	DQ780445	—	—	endophyte	Promputtha et al. (2007)
Xylaria sp.	NR-2006-A59	DQ480344	—	—	*Garciniaviridis* endophyte	Phongpaichit et al. (2006)
Xylaria sp.	PMA P055	EF423534	—	—	*Inga* sp.	Gilbert & Webb (2007)
Xylaria sp.	RQ-NB3-1	AB465207	—	—	*Quercus crispula* leaf litter	Unpublished
Xylaria sp.	XF13	HQ435669	—	—	endophyte	Govinda Rajulu et al. (2013)
Xylaria telfairii	HAST:421	—	—	GQ848350	Dead wood	Hsieh et al. (2010)
Xylaria telfairii	HAST:90081901	—	—	GQ848351	Bark	Hsieh et al. (2010)
Xylariaceae sp.	CHTAE14	JF773597	—	—	*Taxus globosa* endophyte	Unpublished
Xylariaceae sp.	Vega190	EU009987	—	—	*Coffea arabica* peduncle endophyte	Vega et al. (2010)

T denotes sequence from ex-type culture

Arnold, A. E., Miadlikowska, J., Higgins, K. L., Sarvate, S. D., Gugger, P., Way, A., ... & Lutzoni, F. (2009). A phylogenetic estimation of trophic transition networks for ascomycetous fungi: are lichens cradles of symbiotrophic fungal diversification?. Systematic biology, 58(3), 283-297.

Davis, E. C., & Shaw, A. J. (2008). Biogeographic and phylogenetic patterns in diversity of liverwort-associated endophytes. *American Journal of Botany* , *95* (8), 914-924.

Davis, E. C., Franklin, J. B., Shaw, A. J., & Vilgalys, R. (2003). Endophytic Xylaria (Xylariaceae) among liverworts and angiosperms: phylogenetics, distribution, and symbiosis. *American Journal of Botany* , *90* (11), 1661-1667.

de Souza Leite, T., Cnossen-Fassoni, A., Pereira, O. L., Mizubuti, E. S. G., de Araújo, E. F., & de Queiroz, M. V. (2013). Novel and highly diverse fungal endophytes in soybean revealed by the consortium of two different techniques. Journal of Microbiology, 51(1), 56-69.

Douanla-Meli, C., & Langer, E. (2012). Diversity and molecular phylogeny of fungal endophytes associated with Diospyros crassiflora. *Mycology* , *3* (3), 175-187.

Gilbert, G. S., & Webb, C. O. (2007). Phylogenetic signal in plant pathogen–host range. *Proceedings of the National Academy of Sciences* , *104* (12), 4979-4983.

Govinda Rajulu, M. B., Thirunavukkarasu, N., Babu, A. G., Aggarwal, A., Suryanarayanan, T. S., & Reddy, M. S. (2013). Endophytic Xylariaceae from the forests of Western Ghats, southern India: distribution and biological activities. *Mycology* , *4* (1), 29-37.

Higgins, K. L., Coley, P. D., Kursar, T. A., & Arnold, A. E. (2011). Culturing and direct PCR suggest prevalent host generalism among diverse fungal endophytes of tropical forest grasses. *Mycologia* , *103* (2), 247-260.

Hsieh, H. M., Lin, C. R., Fang, M. J., Rogers, J. D., Fournier, J., Lechat, C., & Ju, Y. M. (2010). Phylogenetic status of Xylaria subgenus Pseudoxylaria among taxa of the subfamily Xylarioideae (Xylariaceae) and phylogeny of the taxa involved in the subfamily. Molecular Phylogenetics and Evolution, 54(3), 957-969.

Johnston, P. R., Johansen, R. B., Williams, A. F., Wikie, J. P., & Park, D. (2012). Patterns of fungal diversity in New Zealand Nothofagus forests. Fungal biology, 116(3), 401-412.

Ju, Y. M., Hsieh, H. M., Rogers, J. D., Fournier, J., Jaklitsch, W. M., & Courtecuisse, R. (2012). New and interesting penzigioid Xylaria species with small, soft stromata. Mycologia, 104(3), 766-776.

Ju, Y. M., Hsieh, H. M., Vasilyeva, L., & Akulov, A. (2009). Three new Xylaria species from Russian Far East. *Mycologia* , *101* (4), 548-553.

Okane, I., & Nakagiri, A. (2007). Taxonomy of an anamorphic xylariaceous fungus from a termite nest found together with Xylaria angulosa. *Mycoscience* , *48* (4), 240-249.

Phongpaichit, S., Rungjindamai, N., Rukachaisirikul, V., & Sakayaroj, J. (2006). Antimicrobial activity in cultures of endophytic fungi isolated from Garcinia species. *FEMS Immunology & Medical Microbiology* , *48* (3), 367-372.

Promputtha, I., Lumyong, S., Dhanasekaran, V., McKenzie, E. H. C., Hyde, K. D., & Jeewon, R. (2007). A phylogenetic evaluation of whether endophytes become saprotrophs at host senescence. *Microbial Ecology* , *53* (4), 579-590.

Rhee, Y. J., Hillier, S., Pendlowski, H., & Gadd, G. M. (2014). Pyromorphite formation in a fungal biofilm community growing on lead metal. *Environmental Microbiology* , *16* (5), 1441-1451.

Richardson, S. N., Walker, A. K., Nsiama, T. K., McFarlane, J., Sumarah, M. W., Ibrahim, A., & Miller, J. D. (2014). Griseofulvin-producing Xylaria endophytes of Pinus strobus and Vaccinium angustifolium: evidence for a conifer-understory species endophyte ecology. *Fungal Ecology* , *11* , 107-113.

Shibuya, H., Kitamura, C., Maehara, S., Nagahata, M., Winarno, H., Simanjuntak, P., ... & Ohashi, K. (2003). Transformation of Cinchona alkaloids into 1-N-oxide derivatives by endophytic Xylaria sp. isolated from Cinchona pubescens. *Chemical and pharmaceutical bulletin* , *51* (1), 71-74.

Smith, S. A., Tank, D. C., Boulanger, L. A., Bascom-Slack, C. A., Eisenman, K., Kingery, D., ... & Keehner, J. (2008). Bioactive endophytes warrant intensified exploration and conservation. *PLoS One* , *3* (8), e3052.

Thomas, D. C., Vandegrift, R., Ludden, A., Carroll, G. C., & Roy, B. A. (2016). Spatial ecology of the fungal genus Xylaria in a tropical cloud forest. *Biotropica* , *48* (3), 381-393.

U'Ren, J. M., Miadlikowska, J., Zimmerman, N. B., Lutzoni, F., Stajich, J. E., & Arnold, A. E. (2016). Contributions of North American endophytes to the phylogeny, ecology, and taxonomy of Xylariaceae (Sordariomycetes, Ascomycota). *Molecular Phylogenetics and Evolution* , *98* , 210-232.

U'Ren, J. M., Dalling, J. W., Gallery, R. E., Maddison, D. R., Davis, E. C., Gibson, C. M., & Arnold, A. E. (2009). Diversity and evolutionary origins of fungi associated with seeds of a neotropical pioneer tree: a case study for analysing fungal environmental samples. Mycological Research, 113(4), 432-449.

U'Ren, J. M., Lutzoni, F., Miadlikowska, J., Laetsch, A. D., & Arnold, A. E. (2012). Host and geographic structure of endophytic and endolichenic fungi at a continental scale. American Journal of Botany, 99(5), 898-914.

Vega, F. E., Simpkins, A., Aime, M. C., Posada, F., Peterson, S. W., Rehner, S. A., ... & Arnold, A. E. (2010). Fungal endophyte diversity in coffee plants from Colombia, Hawai'i, Mexico and Puerto Rico. *fungal ecology* , *3* (3), 122-138.

Vincent, J. B., Weiblen, G. D., & May, G. (2016). Host associations and beta diversity of fungal endophyte communities in New Guinea rainforest trees. *Molecular ecology* , *25* (3), 825-841.

Voglmayr, H., Friebes, G., Gardiennet, A., & Jaklitsch, W. M. (2018). Barrmaelia and Entosordaria in Barrmaeliaceae (fam. nov., Xylariales) and critical notes on Anthostomella-like genera based on multigene phylogenies. *Mycological progress* , *17* (1-2), 155-177.

Wendt, L., Sir, E. B., Kuhnert, E., Heitkämper, S., Lambert, C., Hladki, A. I., ... & Stadler, M. (2018). Resurrection and emendation of the Hypoxylaceae, recognised from a multigene phylogeny of the Xylariales. *Mycological Progress* , *17* (1-2), 115-154.
